# Antimicrobial susceptibility profiles of invasive bacterial infections among children from low- and middle-income countries in the Western Pacific Region (WPRO) – a systematic review and meta-analysis

**DOI:** 10.1016/j.lanwpc.2024.101177

**Published:** 2024-08-31

**Authors:** Nerida Moore, Elizabeth A. Ashley, Benjamin F.R. Dickson, Anousone Douangnouvong, Pathana Panyaviseth, Paul Turner, Phoebe C.M. Williams

**Affiliations:** a Royal Darwin Hospital, 105 Rocklands Dr, Tiwi, NT, 0810, Australia; b Lao-Oxford-Mahosot Hospital-Wellcome Trust Research Unit, Microbiology Laboratory, Mahosot Hospital, Vientiane, Lao PDR; c Centre for Tropical Medicine and Global Health, Nuffield Department of Medicine, University of Oxford, Oxford, UK; d Faculty of Medicine, School of Public Health, The University of Sydney, Sydney, NSW, Australia; e Sydney Institute of Infectious Diseases (Sydney ID), Sydney, NSW, Australia; f University of Health Sciences of Lao PDR (UHS-Laos) - Faculty of Medicine; g Cambodia Oxford Medical Research Unit, Angkor Hospital for Children, Siem Reap, Cambodia; h Department of Infectious Diseases, Sydney Children's Hospital Network, Sydney, NSW, Australia

**Keywords:** Antimicrobial, Susceptible, Resistant, Paediatric, Neonate, Child, Infant, Western Pacific Region

## Abstract

**Background:**

Antimicrobial resistance increasingly impacts paediatric mortality, particularly in resource-constrained settings. We aimed to evaluate the susceptibility profiles of bacteria causing infections in children from the Western Pacific region.

**Methods:**

We conducted a systematic review and meta-analysis of bacteria responsible for common infections in children. We included studies published from January 2011 to December 2023 (PROSPERO CRD42021248722). Pooled susceptibilities were evaluated against empiric antibiotics recommended to treat common clinical syndromes.

**Findings:**

Fifty-one papers met inclusion criteria, incorporating 18,330 bacterial isolates. Of available published data, only six countries from the region were represented. *Escherichia coli* revealed a pooled susceptibility to ampicillin of 17% (95% CI 12–23%, *n* = 3292), gentamicin 63% (95% CI 59–67%, *n* = 3956), and third-generation cephalosporins 59% (95% CI 49–69%, *n* = 3585). Susceptibility of *Klebsiella* spp. to gentamicin was 71% (95% CI 61–80%, *n* = 2323), third-generation cephalosporins 35% (95% CI 22–49%, *n* = 2076), and carbapenems 89% (95% CI 78–97%, *n* = 2080). Pooled susceptibility of *Staphylococcus aureus* to flucloxacillin was 72% (95% CI 58–83%, *n* = 1666), and susceptibility of *Streptococcus pneumoniae* meningitis isolates to ampicillin was 26% (95% CI 11–44%, *n* = 375), and 63% (95% CI 40–84%, *n* = 246) to third-generation cephalosporins.

**Interpretation:**

The burden of antimicrobial resistance among bacteria responsible for common infections in children across the Western Pacific region is significant, and the currently recommended World Health Organization antibiotics to treat these infections may be inefficacious. Strategies to improve the availability of high-quality data to understand the burden of antimicrobial resistance in the region are necessary.

**Funding:**

The study was supported by an 10.13039/100015539Australian Government10.13039/501100000925National Health and Medical Research Council Investigator Grant. This research was funded in part by the 10.13039/100010269Wellcome Trust [220211/Z/20/Z]. For the purpose of Open Access, the author has applied a CC BY public copyright licence to any Author Accepted Manuscript version arising from this submission.

**Research in context**

**Evidence before this study**

Resistance to antimicrobials threatens to undermine advances made in child survival, particularly in resource-constrained settings. Previous research has evaluated the burden of antimicrobial resistance in children from geographic settings across Asia and Africa, but there is inadequate evidence of the evaluation of paediatric data in the WHO-defined Western Pacific Region.

**Added value of this study**

Data were pooled from 51 studies examining invasive bacterial infections in paediatric patients across six low- or middle-income countries from the region. A total of 18,330 isolates met the predefined inclusion criteria; of which 15,214 were included in a meta-analysis. We report susceptibility to WHO-recommended antibiotic regimens for important gram-positive and gram-negative bacteria.

**Implications of all the available evidence**

This review revealed alarming antibiotic resistance to empirical WHO-recommended regimens used to treat common infectious syndromes in children from the Western Pacific region. It also highlighted a distinct lack of high-quality published paediatric antimicrobial resistance data from the region. Our findings suggest that current antibiotic recommendations and prescribing practices may require reconsideration in many resource-constrained settings within the region. Enhanced efforts to strengthen antimicrobial surveillance and stewardship remains critically important.

## Introduction

Resistance to antimicrobials threatens to undermine advances made in child survival in low- or middle-income countries (LMICs) of the World Health Organization (WHO)- defined Western Pacific Region (WPRO).^1^ In 2016, 5.6 million children under the age of five died in the WPRO region, with most deaths attributed to preventable and treatable infectious diseases such as pneumonia, diarrhoeal illness, and neonatal sepsis.^1^ The rise of antimicrobial resistance (AMR) within the region may stagnate progress in child health outcomes, as common infections may no longer be curable with currently available antibiotics.^2^

AMR is surging globally and has disproportionately affected children.^3^ In 2019, one in five deaths attributable to AMR occurred in young children, largely secondary to previously treatable infections.^3^ Neonates are a cohort of particular concern, with multidrug-resistant (MDR) pathogens accounting for 214,000 neonatal sepsis deaths globally each year.^4^ In the WPRO, region AMR is an increasingly urgent threat.^1^^,^^2^ While efforts to address the drivers of AMR are underway, there has been varied success across the region.^5^ In October 2014, WHO Member States endorsed the Action Agenda for Antimicrobial Resistance in WPRO; whilst promising, most progress enacting these plans has been made in high-income countries.^6^ As of December 2023 only six of the 18 WPRO LMICs have enrolled in the WHO-led Global Antimicrobial Resistance and Use Surveillance System (GLASS); Cambodia, Lao People's Democratic Republic, Malaysia, Papua New Guinea, Philippines and Viet Nam.^7^ Subsequently, only a small amount of routine AMR data from high-burden LMICs in the region are available.^4^

To enhance global antimicrobial stewardship, the WHO Essential Medicines List for Children classifies antibiotics into three categories (Access, Watch, and Reserve).^8^ While the use of Reserve antibiotics remains low in children, an alarming increase in the empiric use of Watch antibiotics has been reported in various countries, including within the WPRO region.^9^ This may reflect an absence of antibiotic stewardship strategies or signal the ineffectiveness of antibiotics in the ‘Access’ group.^10^ To address current AMR knowledge gaps in children within the WPRO region, this review aimed to examine the susceptibility profiles of key bacterial pathogens responsible for common invasive bacterial infections among children from LMICs of the Western Pacific region, and to evaluate their susceptibility against current WHO-recommended empiric antibiotics, and (commonly-prescribed) carbapenems.

## Methods

### Search strategy and selection criteria

**Fig. 1:**
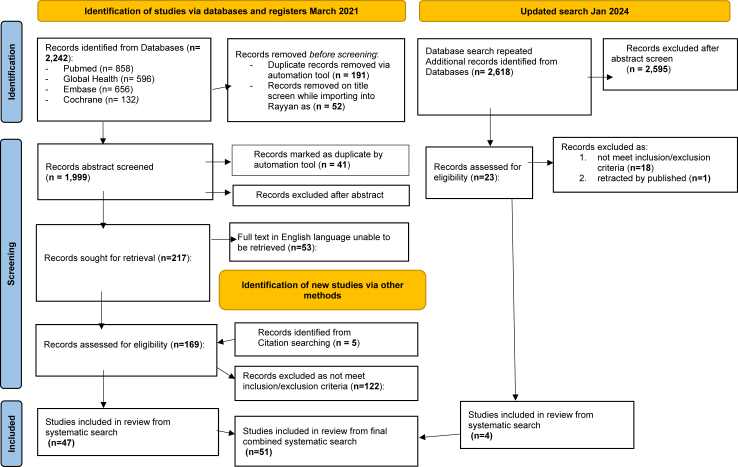
Prisma diagram.^11^

A systematic literature review was performed in accordance with a pre-defined study protocol (PROSPERO registration CRD42021248722). The first search was conducted on 26 March 2021, and updated on 15 January 2024, to include papers published between 1 January 2011 and 31 December 2023. Fig. 1 depicts the search process in a PRISMA flow diagram. We searched Embase (Ovid), Global Health (EBSCO), PubMed, and the Cochrane Database of Systematic Reviews (Cochrane Library) to identify studies that reported bacterial infections in children from LMICs within the WPRO region. Search terms are described in supplementary data (Supplement 1). A citation search was conducted to identify additional studies (grey literature) by searching reference lists of publications eligible for full-text review.

All observational epidemiological studies published in English within peer-reviewed journals were included. Studies were deemed relevant if they met the following inclusion criteria: (i) research on bacterial infections (incidence, prevalence, aetiology or clinical infection); (ii) isolates from sterile sites only (plus urine if reporting clinical urinary tract infections, and stool if *Shigella* spp. or *Salmonella* spp. clinical infections); (iii) specified paediatric data (age up to and including 18 years); (iv) antimicrobial susceptibility testing (AST) methods documented and in line with Clinical and Laboratory Standards Institute (CLSI)/European Committee on Antimicrobial Susceptibility Testing (EUCAST) recommendations, and; (v) research specifically examining bacterial infections caused by organisms found on the Global Antimicrobial Resistance and Use Surveillance System (GLASS) 2020 list of organisms (i.e. *Escherichia coli*, *Klebsiella* spp., *Acinetobacter* spp., *Staphylococcus aureus*, *Streptococcus pneumoniae*, *Salmonella* spp., *Shigella* spp., *Neisseria gonorrhoeae*, and *Pseudomonas aeruginosa*), plus other important pathogens causing invasive infections in children (i.e. *Streptococcus agalactiae*, *Streptococcus pyogenes*, *Haemophilus influenzae*, and *Neisseria meningitidis*).

Countries were limited to those within the WHO-defined WPRO region, excluding high-income countries as defined by the World Bank.^12^^,^^13^ The following 18 countries were included in the search: Cambodia, China, Fiji, Kiribati, Lao People's Democratic Republic, Malaysia, Marshall Islands, Micronesia, Mongolia, Niue, Papua New Guinea, Philippines, Samoa, Solomon Islands, Tonga, Tuvalu, Vanuatu, and Viet Nam.

Abstracts and titles were compiled, and duplicates removed. NM reviewed all citations to determine eligibility; EAA, AD, and PP provided the independent second review. Disagreement over inclusion was resolved by PCMW. All eligible articles were retrieved in full text. Studies were excluded if they presented data aggregated with regions outside the pre-defined region, or if published data were aggregated with adult data. Studies were also excluded if they reported only isolates from carriage or colonisation studies, if they were case reports or small case series involving fewer than ten participants, or if they were studies focussed on high-risk populations only (i.e. children living with HIV, profoundly immunosuppressed populations, children with severe acute malnutrition).

### Data extraction

Data from included studies were independently extracted by two investigators (NM and BFRD) (Supplement 2). Extracted data included the publication year, location, setting, population, age, study design, infectious syndrome, organism, microbiological methods of bacterial isolation, and antimicrobial susceptibility testing (AST) results.

### Outcome measurement

The primary outcome of this review was the weighted, pooled susceptibility estimates of pre-defined key bacterial pathogens, evaluated against antimicrobials recommended in WHO treatment guidelines for children in limited resource settings.^14^ Susceptibility against carbapenems, frequently prescribed as an empirical therapy in the context of rising global AMR, were also evaluated.

### Quality assessment

The Microbiology Investigation Criteria for Reporting Objectively (MICRO) framework was used to assess the quality of published microbiology data.^15^ The Grades of Recommendation, Assessment, Development and Evaluation Working Group (GRADE) method was used to summarise the quality of evidence for each study by assessment of study type, quality, limitations, inconsistency, and risk of bias.^16^ MICRO and GRADE reviews were undertaken independently by three authors (NM, BFRD and PCMW); discrepancies were resolved via consensus. The results of these quality assessments are summarised in Supplement 2.

### Statistical analysis

Meta-analyses for a single proportion were undertaken in Stata version 18 (Stata Corporation, TX) using the in-built *meta* command. Effect sizes were determined with a Freeman-Tukey-transformed proportion. Summary estimates were provided, along with 95% confidence intervals (95% CIs). A random-effects model was used to account for the expected heterogeneity in susceptibilities between study populations with study weighting done using the Restricted Maximum Likelihood (REML) estimation.^17^ The I^2^ and the τ^2^ statistics were used to evaluate heterogeneity between studies and subgroups.^18^ Egger's test and funnel-plots were used to assess for potential publication bias (small-study effects).^19^ Possible sources of heterogeneity between studies were investigated through subgroup analyses (year of publication, country of origin, and clinical syndrome). Meta-regression for each outcome was performed to assess for evidence of a linear relationship between susceptibility and year. A value of p < 0.05 was considered significant in all analyses.

### Role of the funding source

PCMW is supported by an NHMRC Investigator Grant (119735). NHMRC had no involvement in the design or conduct of the research. This research was funded in part, by the Wellcome Trust [220211/Z/20/Z]. For the purpose of Open Access, the author has applied a CC BY public copyright licence to any Author Accepted Manuscript version arising from this submission. Wellcome Trust had no involvement in the design or conduct of the research.

## Results

### Characteristics of the included studies

The combined search identified 4860 relevant papers published between 1 January 2011 and 31 December 2023 (Fig. 1). Abstract reviews excluded 4620 papers. Of the 240 papers sought for full-text review, 53 were unable to be retrieved in full-text English language and were almost exclusively Chinese studies (one Malaysian study). The remaining 187 papers, plus an additional five identified from grey literature screening, underwent full-text review. This resulted in a total of 51 papers for inclusion.^20^, ^21^, ^22^, ^23^, ^24^, ^25^, ^26^, ^27^, ^28^, ^29^, ^30^, ^31^, ^32^, ^33^, ^34^, ^35^, ^36^, ^37^, ^38^, ^39^, ^40^, ^41^, ^42^, ^43^, ^44^, ^45^, ^46^, ^47^, ^48^, ^49^, ^50^, ^51^, ^52^, ^53^, ^54^, ^55^, ^56^, ^57^, ^58^, ^59^, ^60^, ^61^, ^62^, ^63^, ^64^, ^65^, ^66^, ^67^, ^68^, ^69^, ^70^

**Fig. 2:**
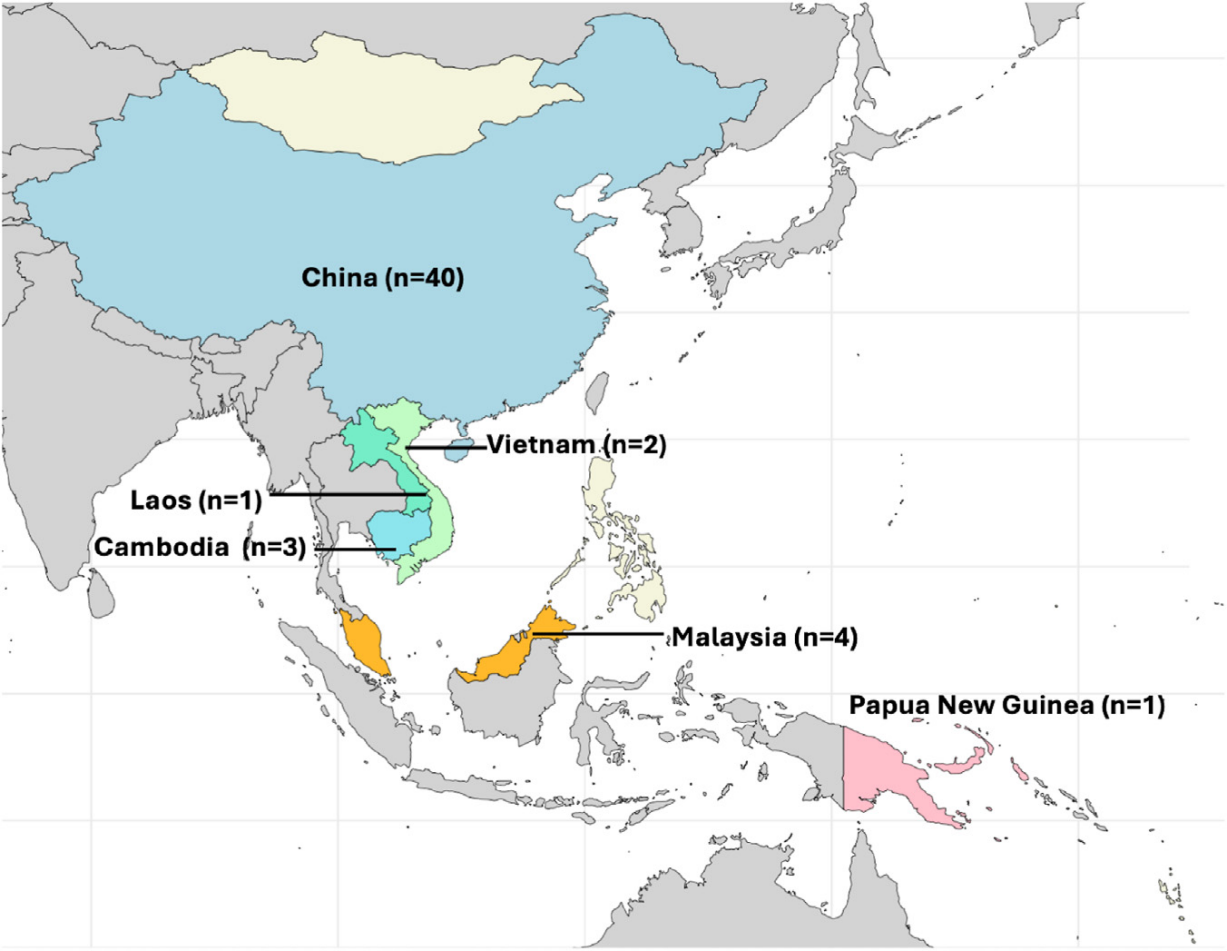
**Number of papers by country**.

Of the 51 included papers, six of the 18 WPRO LMICs were represented (Fig. 2). Most papers were from China (*n* = 40 papers), followed by Malaysia (*n* = 4), Cambodia (*n* = 3), Vietnam (*n* = 2), Papua New Guinea (*n* = 1) and Laos (*n* = 1). Twenty-seven studies included all children up to adolescence (maximum upper age limit 18 years),^20^, ^21^, ^22^, ^23^, ^24^, ^25^, ^26^, ^27^, ^28^, ^29^, ^30^, ^31^, ^32^, ^33^, ^34^, ^35^, ^36^, ^37^, ^38^, ^39^, ^40^, ^41^, ^42^, ^43^, ^44^^,^^69^^,^^70^ ten included neonates less than one month of age,^45^, ^46^, ^47^, ^48^, ^49^, ^50^, ^51^, ^52^^,^^67^^,^^68^ three focussed on infants zero to 12 months,^53^, ^54^, ^55^ and four on children zero to five years.^56^, ^57^, ^58^, ^59^ Seven studies did not specify the age range of their paediatric population.^60^, ^61^, ^62^, ^63^, ^64^, ^65^, ^66^

Six studies specifically examined community-acquired infections^20^^,^^21^^,^^25^^,^^41^^,^^54^^,^^59^; three nosocomial infections^23^^,^^45^^,^^52^; and three studies examined both.^50^^,^^63^^,^^67^ The remaining 39 studies (76%) did not identify whether infections were community or hospital-acquired. One study was conducted in a rural setting,^20^ while the remainder of the studies were conducted in urban settings (almost exclusively tertiary health facilities).

The study design of 49 studies was a case series; ten prospective,^20^^,^^24^^,^^25^^,^^28^^,^^30^^,^^37^^,^^40^^,^^54^^,^^57^^,^^64^ 33 retrospective,^21^, ^22^, ^23^^,^^26^^,^^27^^,^^29^^,^^31^^,^^33^, ^34^, ^35^^,^^39^^,^^41^, ^42^, ^43^, ^44^, ^45^, ^46^, ^47^, ^48^, ^49^, ^50^, ^51^, ^52^^,^^55^^,^^56^^,^^60^, ^61^, ^62^, ^63^^,^^66^^,^^67^^,^^69^^,^^70^ and six not specified.^32^^,^^36^^,^^38^^,^^53^^,^^58^^,^^65^ There was one case–control study,^59^ and one cross-sectional study.^68^ All studies reported cumulative incidence data. There were no cohort studies identified.

Most studies (34 of 51) reported data from one bacterial clinical syndrome. These included ten studies examining neonatal sepsis/meningitis^45^^,^^47^, ^48^, ^49^, ^50^, ^51^, ^52^^,^^54^^,^^67^^,^^68^; five evaluating paediatric sepsis^21^^,^^23^^,^^25^^,^^43^^,^^63^; five paediatric meningitis^26^^,^^28^^,^^34^^,^^39^^,^^64^; nine studies focussing on acute infectious diarrhoea^32^^,^^37^^,^^44^^,^^56^^,^^58^, ^59^, ^60^^,^^65^^,^^66^; two assessing urinary tract infections^31^^,^^61^; and two on bone and joint infections^20^^,^^27^ while one focussed on enteric fever.^35^ Sixteen studies reported only one bacterial pathogen; the majority of these (*n* = 8) focussed on invasive *S. pneumoniae* infections.^22^^,^^24^^,^^30^^,^^33^^,^^36^^,^^38^^,^^40^^,^^57^

Across the included studies, a total of 38,314 bacterial isolates were reported. AST information was available for 29,236 isolates, of which 18,330 met the pre-defined inclusion criteria for relevant pathogens. Of these, 15,214 were tested against at least one antimicrobial recommended in WHO empirical treatment guidelines,^14^ or carbapenems. Not all isolates were tested against the complete set of WHO-recommended empiric antibiotics. Across the studies, only 57% (29/51) reported denominator data (i.e. the total number of cultures collected from patients over their study period which summated to 496,492 cultures for those reported).

### Quality of the evidence

Only one study was defined as moderate-quality evidence (GRADE level B),^23^ while 23 studies were low-quality (GRADE level C),^20^^,^^22^^,^^24^, ^25^, ^26^^,^^29^^,^^30^^,^^32^^,^^37^^,^^40^^,^^42^, ^43^, ^44^^,^^47^^,^^48^^,^^54^^,^^57^^,^^63^^,^^64^^,^^67^, ^68^, ^69^, ^70^ and the remaining 27 very low quality (GRADE level D).^21^^,^^27^^,^^28^^,^^31^^,^^33^, ^34^, ^35^, ^36^^,^^38^^,^^39^^,^^41^^,^^45^^,^^46^^,^^49^, ^50^, ^51^, ^52^, ^53^^,^^55^^,^^56^^,^^58^, ^59^, ^60^, ^61^, ^62^^,^^65^^,^^66^ GRADE C scores were assigned where studies were single site, retrospective, observational studies, with risk of information, selection or publication bias. In addition to GRADE C features, very low-quality (GRADE D) publications also had small sample sizes, missing data, and errors in reporting.

Thirteen studies had overt identification and/or AST errors either detected or suspected (MICRO grade E).^28^^,^^29^^,^^32^, ^33^, ^34^^,^^36^^,^^39^^,^^42^^,^^47^^,^^48^^,^^50^^,^^52^^,^^66^ Thirty nine studies (76%) provided information about the susceptibility testing methods used (agar diffusion *n* = 28, CLSI broth microdilution *n* = 1, VITEK 2 *n* = 8, Phoenix 100 *n* = 1, MicroScan *n* = 1). The criteria to define susceptible and resistant categories was provided for 45 studies (88%), where the Clinical Laboratory and Standards Institute guidelines (CLSI) was used in all of these. Eighteen studies (35%) described use of internal quality controls. None of the studies included details of external quality assurance (EQA) programme participation.

### Meta-analysis

Fifty-one studies reporting 15,214 relevant bacterial isolates were included in the meta-analysis; 9863 were gram-negative, and 5351 gram-positive bacteria. Susceptibility profiles of 34 bacteria against commonly prescribed antibiotic combinations underwent meta-analysis (Fig. 3, Fig. 4, Fig. 5, Fig. 6, Fig. 7, and Supplementary Data 1, Figures S1–S5).

**Fig. 3:**
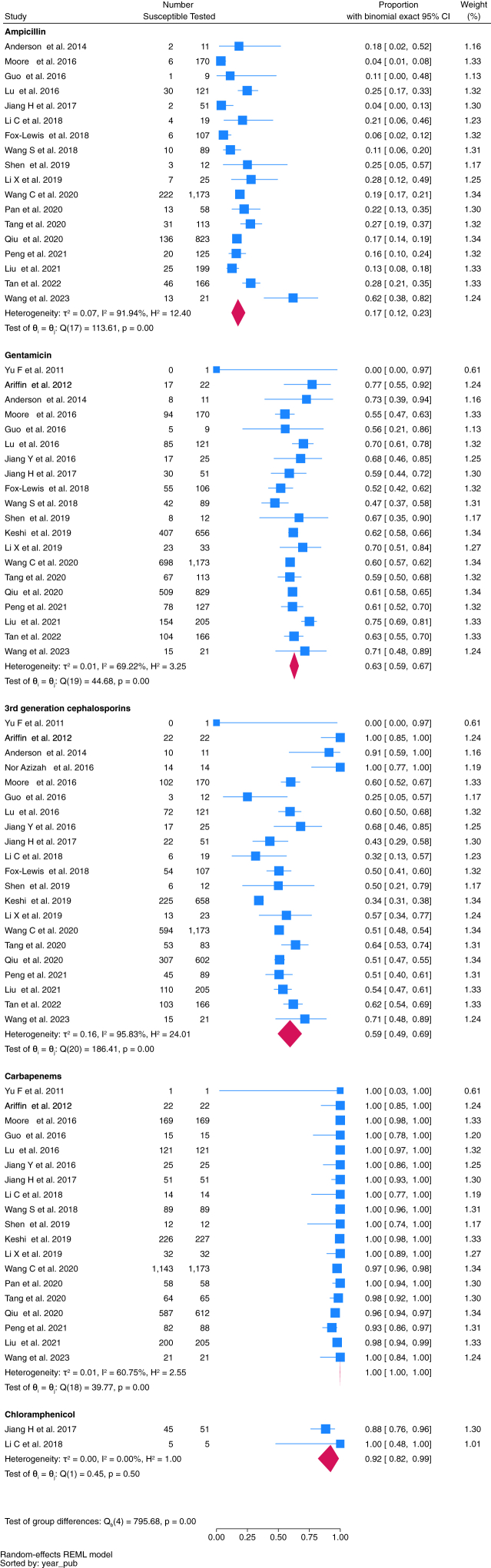
Proportion of *E. coli* isolates susceptible to key antimicrobials.

**Fig. 4:**
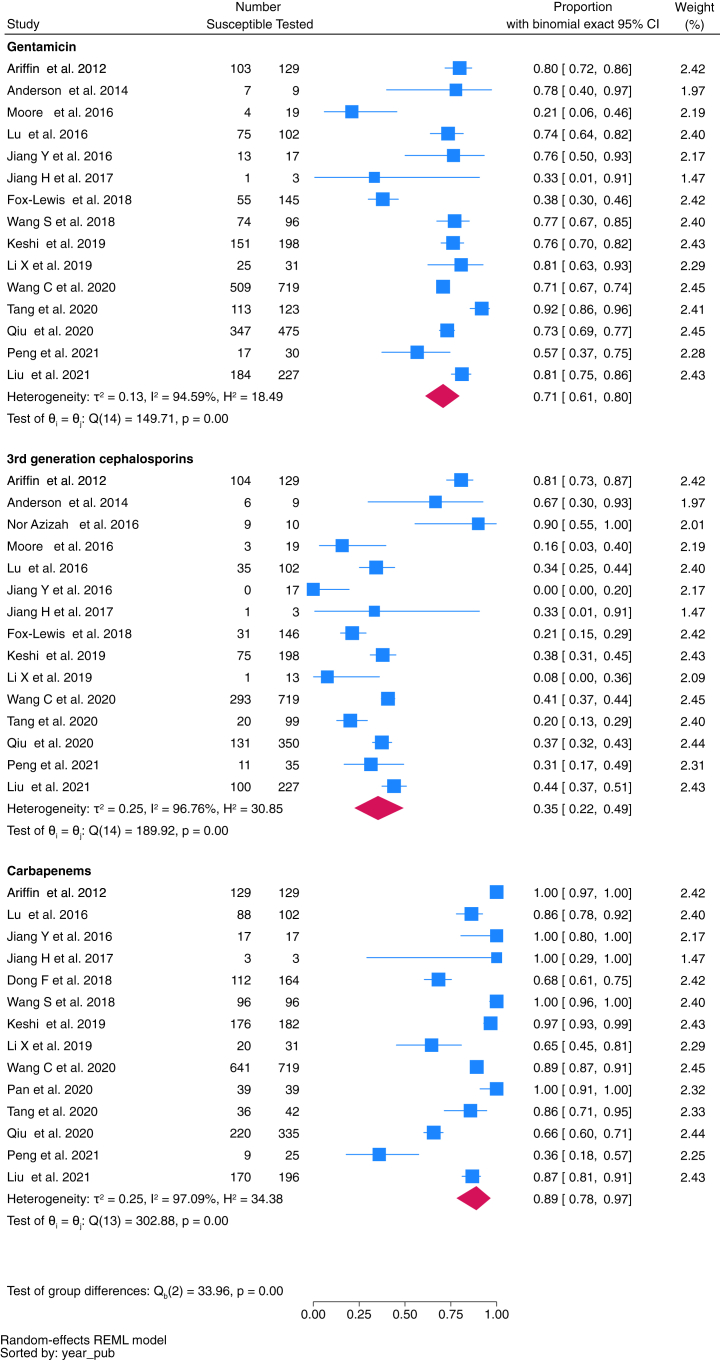
Proportion of *Klebsiella* spp. isolates susceptibile to key antimicrobials.

**Fig. 5:**
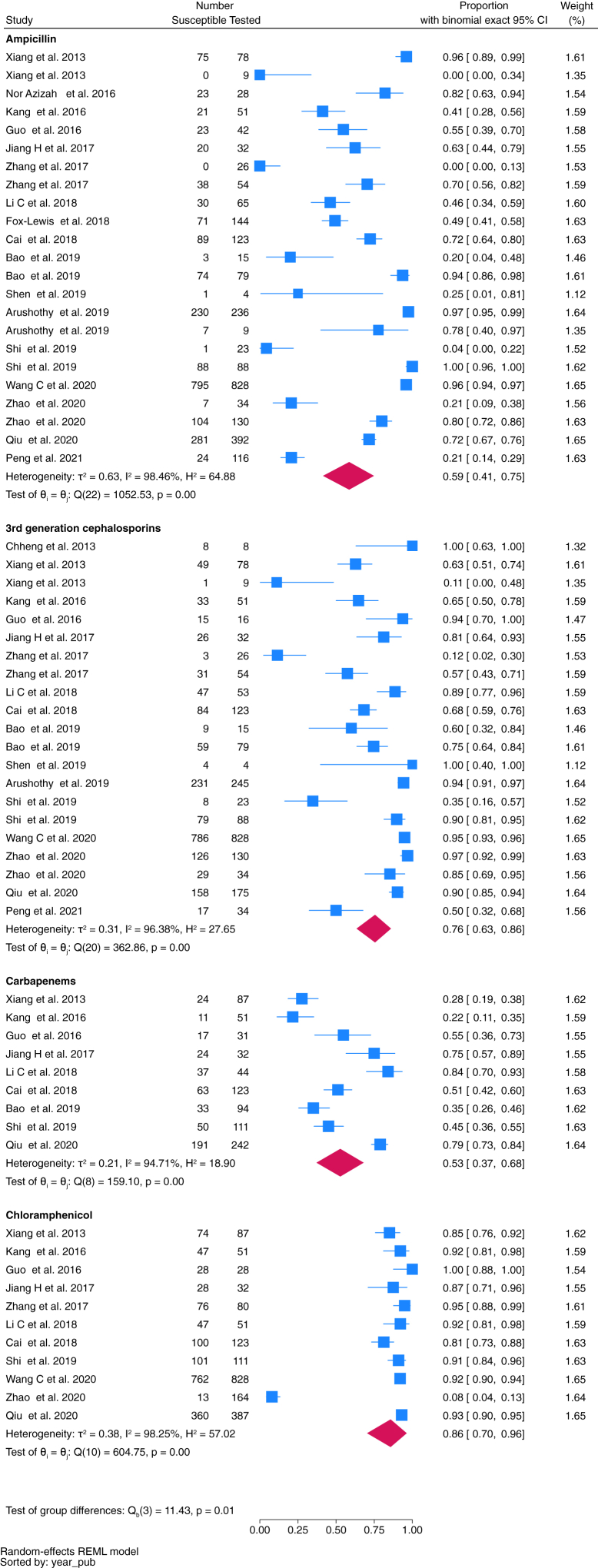
Proportion of *S. pneumoniae* isolates susceptibile to key antimicrobials.

**Fig. 6:**
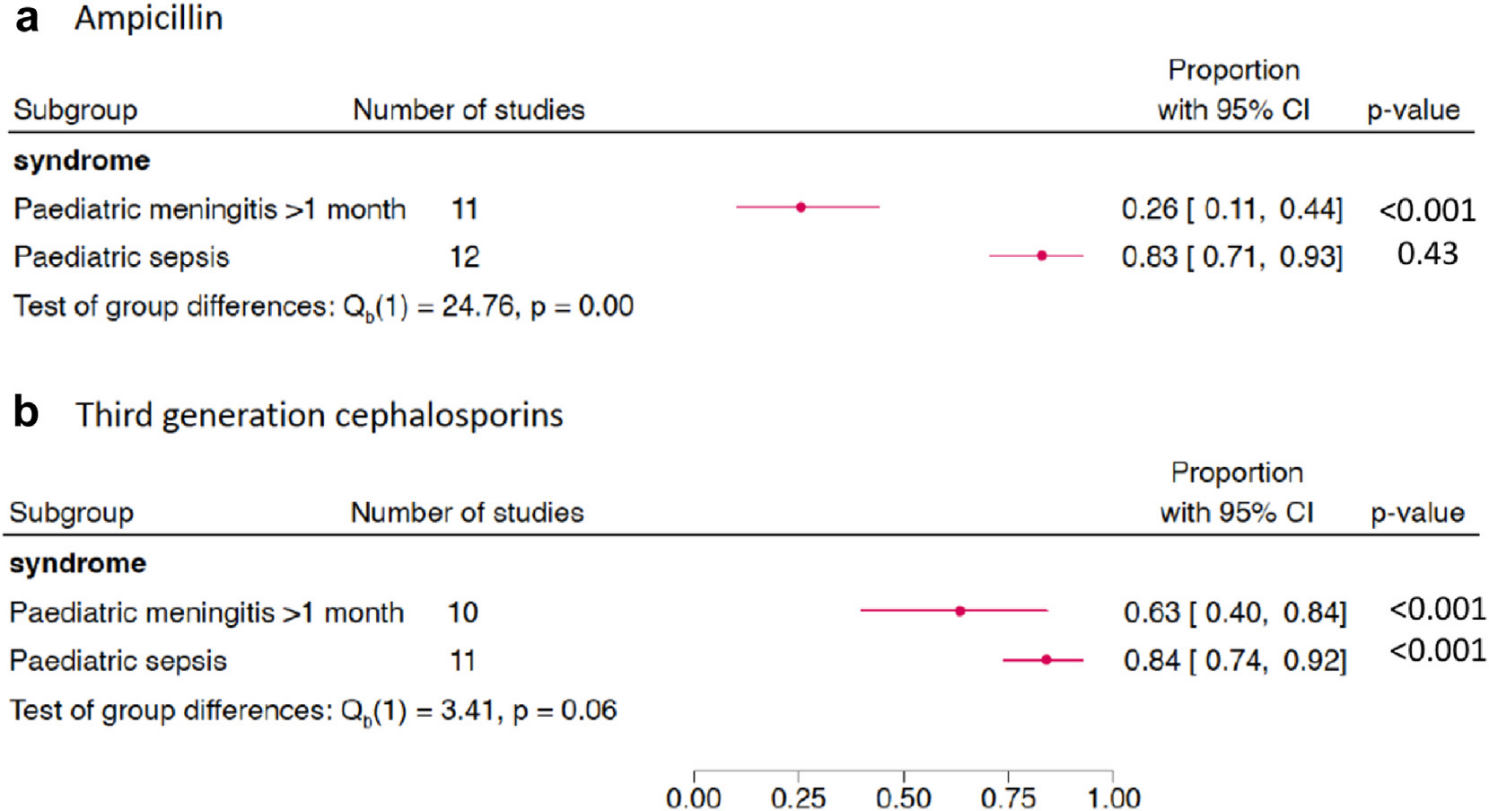
Proportion of *S. pneumoniae* isolates susceptibile to ampicillin and third generation cephalosporins by subgroup paediatric meningitis and sepsis.

**Fig. 7:**
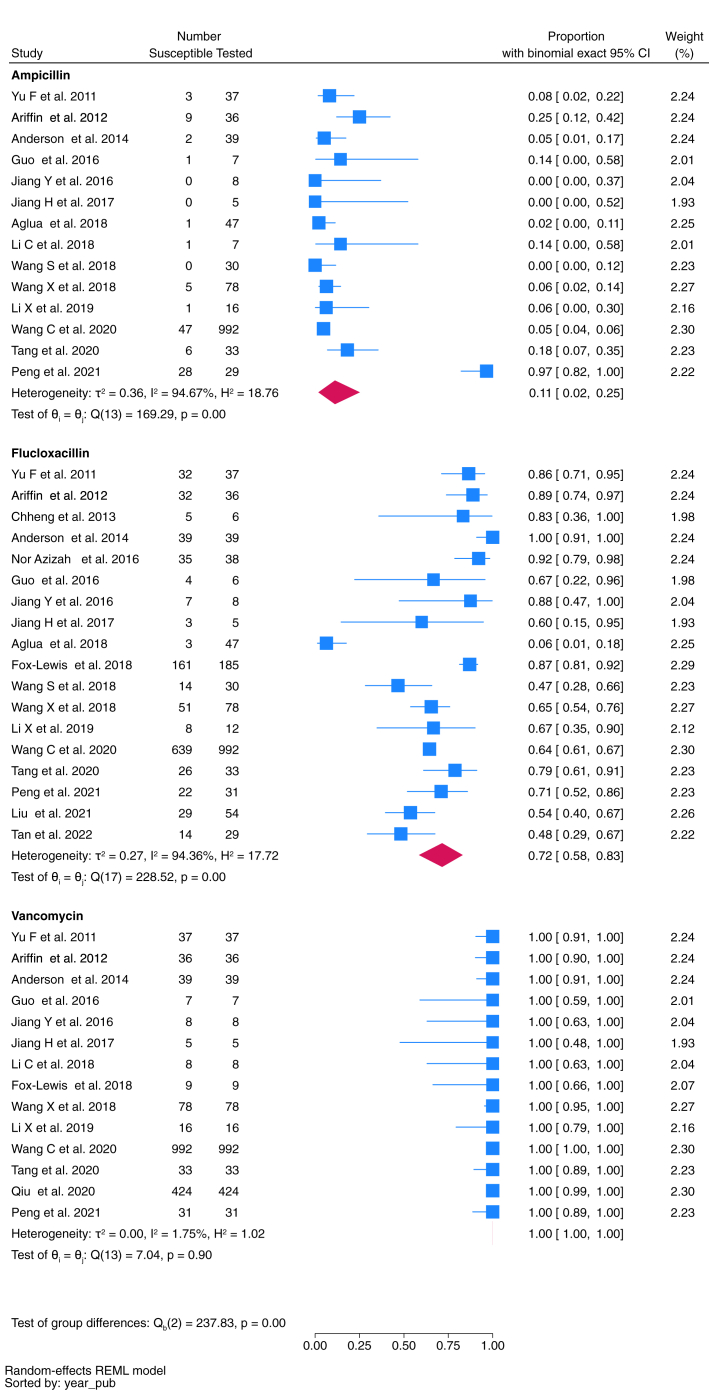
Proportion of *S. aureus* isolates susceptible to key antimicrobials.

### Susceptibility patterns of gram-negative pathogens

*E. coli* was the most frequently isolated gram-negative pathogen (4294 isolates). Twenty-four studies assessed susceptibility patterns of *E. coli*. When considering key antimicrobials included in WHO paediatric treatment guidelines,^14^ plus carbapenems, the pooled susceptibility for ampicillin was 17% (95% CI 12–23%, *n* = 3292 isolates tested), gentamicin 63% (95% CI 59–67%, *n* = 3956), ceftriaxone or cefotaxime (hereafter referred to as third-generation cephalosporins) 59% (95% CI 49–69%, *n* = 3585), and carbapenems 100% (95% CI 100 to 100%, *n* = 3000) (Fig. 3).

There were nineteen studies assessing susceptibility patterns of *Klebsiella* spp. incorporating 2680 isolates. Pooled susceptibility of *Klebsiella* spp. to gentamicin was 71% (95% CI 61–80%, *n* = 2323 isolates tested), third-generation cephalosporins 35% (95% CI 22–49%, *n* = 2076), and carbapenems 89% (95% CI 78–97%, *n* = 2080) (Fig. 4).

*Salmonella* spp. were frequently isolated gram-negative pathogens responsible for paediatric sepsis and acute infectious diarrhoea (2851 isolates across 19 studies). Non-typhoidal serovars were most common. Pooled susceptibility of *Salmonella* spp. to ampicillin was 38% (95% CI 29–49%, *n* = 2591 isolates tested), third-generation cephalosporins 85% (95% CI 77–92%, *n* = 2456), and carbapenems 100% (95% CI 99–100%, *n* = 952) (Supplementary Data 1, Figure S1).

Nine studies examined susceptibility patterns of *P. aeruginosa*, revealing a pooled susceptibility to gentamicin of 82% (95% CI 46–100%, *n* = 306 isolates tested), and carbapenems of 86% (95% CI 72–96%, *n* = 157) (Supplementary data 1, Figure S2). Seven studies reported *Acinetobacter* spp. susceptibility patterns noting pooled susceptibility to gentamicin of 81% (95% CI 66–93%, *n* = 223), and carbapenems 72% (95% CI 63–80%, *n* = 176) (Supplementary data 1, Figure S3).

Four studies evaluated invasive *H. influenzae* isolates. Across these studies, pooled susceptibility to ampicillin was 65% (95% CI 12–100%, *n* = 85), third-generation cephalosporins 97% (95% CI 90–100%, *n* = 80), chloramphenicol 100% (95% CI 99–100%, *n* = 18), and carbapenems 100% (95% CI 93–100%, *n* = 25) (Supplementary Data 1, Figure S4).

One paper meeting the inclusion criteria assessed *Shigella* spp. in children hospitalised with diarrhoea^59^ Susceptibility to third-generation cephalosporins was 27% (*n* = 62 isolates tested) and ciprofloxacin 98% (*n* = 57).^59^

### Susceptibility patterns of gram-positive pathogens

*S. pneumoniae* was the most frequently reported gram-positive pathogen (4067 isolates), with 18 studies assessing susceptibility patterns of *S. pneumoniae* (Fig. 5). All studies used CLSI breakpoints for *S. pneumoniae* susceptibility testing. Fifteen of the 18 included studies differentiated meningitis and non-meningitis isolates,^21^, ^22^, ^23^, ^24^, ^25^, ^26^^,^^28^^,^^34^^,^^36^^,^^38^, ^39^, ^40^^,^^43^^,^^57^^,^^64^ and of these six explicitly reported the CLSI meningitis and non-meningitis breakpoints.^22^^,^^24^^,^^36^^,^^38^^,^^40^^,^^57^ Pooled susceptibility of paediatric meningitis isolates to ampicillin was 26% (95% CI 11–44%, *n* = 375), and third-generation cephalosporins 63% (95% CI 40–84%, *n* = 246) (Fig. 6a and b). Pooled susceptibility of all non-meningitis isolates to ampicillin was 83% (95% CI 71–93%, *n* = 2231), and third-generation cephalosporins 84% (95% CI 74–92%, *n* = 1859) (Fig. 6a and b). Pooled susceptibility of all isolates (across 12 studies) to chloramphenicol was 86% (95% CI 70–96%, *n* = 1942) (Fig. 5). Nine studies reported susceptibility to carbapenems, with a pooled susceptibility of 53% reported (95% CI 37–68%, *n* = 815) (Fig. 5). Of note, carbapenem susceptibility was reported as less than ampicillin susceptibility in two studies,^30^^,^^33^ and less than third-generation cephalosporin susceptibility in each of the nine studies, an anomaly not accounted for in any of the studies.

*S. aureus* was reported from 2169 isolates across 20 studies. Pooled susceptibility to flucloxacillin was 72% (95% CI 58–83%, *n* = 1666 isolates tested), and vancomycin 100% (95% CI 100–100%, *n* = 1723) (Fig. 7).

Twelve of the included studies assessed susceptibility patterns of *S. agalactiae* (634 isolates). Pooled susceptibility of *S. agalactiae* to ampicillin was 100% (95% CI 100–100%, *n* = 599 isolates tested), third-generation cephalosporins 100% (95% CI 100–100%, *n* = 201), vancomycin 100% (95% CI 100–100%, *n* = 341), and carbapenems 100% (95% CI 98–100%, *n* = 80) (Supplementary Data 1, Figure S5).

### Heterogeneity

Among the 34 bacteria vs antibiotic combinations included in the meta-analyses, median I^2^ was 94.29% (IQR 11.65–95.92%). The majority (53%, 18/34) of outcomes demonstrated considerable heterogeneity (I^2^ greater than 75%) among included studies. Five outcomes had an I^2^ between 50 and 75%; while only eight outcomes had an I^2^ of less than 50% (all of which were less than 25%). The eight outcomes with low levels of heterogeneity included: *S. agalactiae* against ampicillin, third-generation cephalosporins, carbapenems and vancomycin; *S. aureus* against vancomycin; *H. influenzae* against third-generation cephalosporins; *Salmonella* spp. against azithromycin; and *Acinetobacter* spp. against carbapenems.

#### Subgroup analyses and meta-regression

The results of the subgroup analyses for clinical syndrome, year group and country are presented in Supplemental Data 1, Figures S6–S45. The results of the meta-regression by year are shown in Supplemental Data 1, Figures S46 and S47.

#### Susceptibility patterns by clinical syndrome

A subgroup analysis by clinical syndrome was performed. A summary of susceptibility patterns by clinical syndrome are presented in Table 1. Eleven bug–drug combinations showed significant differences between syndromes, however there was significant heterogeneity within at least one of the categories of syndrome for seven (63%) of these. Across all clinical subgroup estimates, the median I^2^ was 69.14% (IQR 0–94.34%), and 89% (IQR 22.35–96.39%) when restricted to categories with at least three studies per outcome.

**Table 1:** Reported susceptibility proportions of the most likely causative organisms by relevant clinical syndrome to WHO-recommended empirical antibiotic regimens.

Organism	Number of studies (number of isolates)^ref^	Weighted, pooled prevalence (susceptibility) estimate% (95% CI)	Heterogeneity		
			p-value^a^	Iˆ2	Tauˆ2
**Neonatal Sepsis/Meningitis**					
***Streptococcus agalactiae***					
Ampicillin	5 (326)^46^^,^^50^^,^^53^^,^^55^^,^^68^	100% (95% CI 100–100%)	0.958	<0.001	<0.001
Gentamicin	–	–	–	–	–
Third-generation cephalosporins	3 (85)^46^^,^^50^^,^^53^	98% (95% CI 93–100%)	0.270	28.26	0.014
***Staphylococcus aureus***					
Flucloxacillin^c^	8 (241)^45^^,^^49^, ^50^, ^51^, ^52^^,^^54^^,^^67^^,^^68^	74% (95% CI 56–89%)	<0.001	87.97	0.246
Vancomycin	5 (132)^45^^,^^49^, ^50^, ^51^^,^^54^	100% (95% CI 99–100%)	0.989	0	<0.001
***Escherichia coli***					
Ampicillin	8 (782)^47^, ^48^, ^49^, ^50^^,^^52^^,^^54^^,^^67^^,^^68^	21% (95% CI 15–27%)	0.001	69.27	0.024
Gentamicin	9 (801)^45^^,^^47^^,^^49^, ^50^, ^51^, ^52^^,^^54^^,^^67^^,^^68^	66% (95% CI 59–73%)	0.001	68.93	0.027
Third-generation cephalosporins	8 (656)^45^^,^^47^^,^^49^, ^50^, ^51^^,^^54^^,^^67^^,^^68^	70% (95% CI 56–83%)	<0.001	91.35	0.143
***Klebsiella*spp.**					
Gentamicin	9 (764)^39^^,^^45^^,^^47^^,^^49^, ^50^, ^51^, ^52^^,^^54^^,^^67^	79% (95% CI 73–85%)	0.001	72.15	0.033
Third-generation cephalosporins	7 (596)^45^^,^^47^^,^^49^, ^50^, ^51^^,^^54^^,^^67^	33% (95% CI 12–59%)	<0.001	96.96	0.425
**Paediatric Sepsis**					
***Staphylococcus aureus***					
Flucloxacillin^c^	5 (1299)^21^^,^^23^^,^^25^^,^^41^^,^^63^	79% (95% CI 65–90%)	<0.001	92.09	0.091
Vancomycin	4 (1503)^13^^,^^23^^,^^41^^,^^43^	100% (95% CI 100–100%)	0.739	0.02	<0.001
***Streptococcus pneumoniae***					
Ampicillin^b^	12 (2231)^21^^,^^24^^,^^30^^,^^33^^,^^36^^,^^38^^,^^40^^,^^43^^,^^57^^,^^63^	83% (95% CI 71–93%)	<0.001	97.63	0.252
Gentamicin	–	–	–	–	–
Third-generation cephalosporins	11 (1859)^22^, ^23^, ^24^, ^25^^,^^30^^,^^33^^,^^36^^,^^38^^,^^40^^,^^43^^,^^57^	84% (95% CI 74–92%)	<0.001	95.56	0.152
***Escherichia coli***					
Ampicillin	4 (2124)^23^^,^^43^^,^^63^^,^^69^	22% (95% CI 5–46%)	<0.001	98.99	0.255
Gentamicin	4 (2129)^23^^,^^43^^,^^63^^,^^69^	60% (95% CI 58–62%)	0.195	0.01	<0.001
Third-generation cephalosporins	5 (1917)^21^^,^^23^^,^^43^^,^^63^^,^^69^	66% (95% CI 43–85%)	<0.001	98.35	0.237
***Klebsiella*spp.**					
Gentamicin	3 (1339)^23^^,^^43^^,^^63^	61% (95% CI 39–81%)	<0.001	98.35	0.155
Third-generation cephalosporins	4 (1225)^21^^,^^23^^,^^43^^,^^63^	44% (95% CI 19–71%)	<0.001	98.38	0.266
***Salmonella*spp.**					
Ampicillin	8 (1037)^21^^,^^23^^,^^29^^,^^35^^,^^42^^,^^43^^,^^63^^,^^70^	51% (95% CI 33–68%)	<0.001	96.39	0.229
Third-generation cephalosporins	8 (902)^21^^,^^23^^,^^29^^,^^35^^,^^42^^,^^43^^,^^63^^,^^70^	94% (95% CI 84–99%)	<0.001	94.34	0.164
***Haemophilus influenzae***					
Ampicillin	2 (64)^26^^,^^28^	78% (95% CI 14–100%)	<0.001	92.01	0.779
Third-generation cephalosporins	1 (57)^63^	–	–	–	–
**Paediatric Meningitis >1 month**					
***Streptococcus pneumoniae***					
Ampicillin^b^	11 (375)^22^^,^^24^^,^^26^^,^^28^^,^^34^^,^^36^^,^^38^, ^39^, ^40^^,^^57^^,^^64^	26% (95% CI 11–44%)	<0.001	91.11	0.322
Gentamicin	1 (32)^28^	–	–	–	–
Third-generation cephalosporins	10 (246)^22^^,^^24^^,^^26^^,^^28^^,^^34^^,^^36^^,^^38^, ^39^, ^40^^,^^64^	63% (95% CI 40–84%)	<0.001	91.32	0.434
Chloramphenicol	3 (241)^26^^,^^28^^,^^34^	95% (95% CI 85–100%)	0.073	63.44	0.048
***Haemophilus influenzae***					
Ampicillin	2 (21)^26^^,^^28^	51% (95% CI 0–100%)	<0.001	93.67	2.066
Third-generation cephalosporins	3 (23)^26^^,^^28^	97% (95% CI 83–100%)	0.679	<0.001	<0.001
Chloramphenicol	2 (18)^26^^,^^28^	100% (95% CI 100–100%)	0.520	<0.001	<0.001
***Escherichia coli***					
Ampicillin	5 (216)^26^^,^^28^^,^^34^^,^^39^^,^^64^	13% (95% CI 5–22%)	0.083	54.19	0.036
Gentamicin	4 (199)^28^^,^^34^^,^^39^^,^^64^	61% (95% CI 54–68%)	0.949	<0.001	<0.001
Third-generation cephalosporins	5 (183)^26^^,^^28^^,^^34^^,^^39^^,^^64^	44% (95% CI 35–52%)	0.353	13.59	0.005
Chloramphenicol	2 (56)^26^^,^^28^	92% (95% CI 82–99%)	0.500	<0.001	<0.001
***Klebsiella*spp.**					
Gentamicin	1 (33)^39^	–	–	–	–
Third-generation cephalosporins	2 (38)^28^^,^^39^	29% (95% CI 14–47%)	0.847	<0.001	<0.001
Chloramphenicol	1 (3)^28^	–	–	–	–
**Acute Infectious Diarrhoea**					
***Shigella*spp.**					
Ciprofloxacin	1 (57)^59^	–	–	–	–
Third-generation cephalosporins	1 (58)^59^	–	–	–	–
***Salmonella*spp.**					
Ciprofloxacin	11 (1634)^27^^,^^29^^,^^32^^,^^37^^,^^42^^,^^44^^,^^52^^,^^58^, ^59^, ^60^^,^^65^^,^^66^	82% (95% CI 68–94%)	<0.001	97.78	0.317
Third-generation cephalosporins	10 (1540)^29^^,^^32^^,^^37^^,^^42^^,^^44^^,^^56^^,^^58^, ^59^, ^60^^,^^65^	80% (95% CI 71–87%)	<0.001	92.51	0.087
Azithromycin	2 (482)^37^^,^^58^	83% (95% CI 79–86%)	0.835	0.00	<0.001
**Urinary Tract Infection**					
***Klebsiella*spp.**					
Co-trimoxazole	1 (19)^61^	–	–	–	–
Gentamicin	2 (826)^31^^,^^61^	50% (95% CI 4–96%)	<0.001	95.62	0.614
***Escherichia coli***					
Co-trimoxazole	1 (170)^61^	–	–	–	–
Ampicillin	1 (170)^61^	–	–	–	–
Gentamicin	2 (826)^31^^,^^61^	59% (95% CI 53–66%)	0.110	60.87	0.006
**Bone and Joint Infection**					
***Staphylococcus aureus***					
Flucloxacillin	2 (84)^20^^,^^27^	44% (95% CI 0–100%)^20^^,^^27^	<0.001	98.55	1.625
Vancomycin	1 (37)^56^	–	–	–	–

a p value < 0.05 suggests significant evidence of heterogeneity.

b Where ampicillin data not reported susceptibility inferred from penicillin. Ampicillin resistance was not reported for *Klebsiella* spp. as this bacteria is intrinsically resistant.

c Where flucloxacillin data was not reported oxacillin was used to predict methicillin susceptibility and infer flucloxacillin susceptibility.

*E. coli* and *Klebsiella* spp. were the most frequently isolated pathogens causing neonatal sepsis/meningitis in this review. Within this subgroup, *E. coli* isolates had a pooled susceptibility to ampicillin of 21% (95% CI 15–27%, *n* = 782 isolates tested), gentamicin 66% (95% CI 59–73%, *n* = 801) and third-generation cephalosporins 70% (95% CI 56–83% *n* = 656). Pooled susceptibility of *Klebsiella* spp. to gentamicin was 79% (95% CI 73–85%, *n* = 764), and third-generation cephalosporins 33% (95% CI 12–59%, *n* = 596).

For paediatric sepsis, the most frequently isolated gram-negative pathogens *E. coli* and *Klebsiella* spp. had pooled susceptibilities to third-generation cephalosporins of 66% (95% CI 43–85%, *n* = 1917 isolates tested), and 44% (95% CI 19–71%, *n* = 1225) respectively. *Salmonella* spp. had a pooled susceptibility to ampicillin in paediatric sepsis of 51% (95% CI 33–68%, *n* = 1037), and third-generation cephalosporins 94% (95% CI 84–99%, *n* = 902). *S. aureus* and *S. pneumoniae* were the most frequently reported gram-positive pathogens in paediatric sepsis. *S. aureus* had a pooled susceptibility to flucloxacillin of 79% (95% CI 65–90%, *n* = 1299 isolates tested). Pooled susceptibility of *S. pneumoniae* against ampicillin was 83%, (95% CI 71–93%, *n* = 2231) and third-generation cephalosporins 84% (95% CI 74–92%, *n* = 1859).

In paediatric meningitis, pooled susceptibility of *S. pneumoniae* against ampicillin was 26% (95% CI 11–44%, *n* = 375). The pooled susceptibility of *S. pneumoniae* to third-generation cephalosporins was 63% (95% CI 40–84%, *n* = 246). In this subgroup *E. coli* had a pooled susceptibility to ampicillin of 13% (95% CI 05–22%, *n* = 216), gentamicin 61% (95% CI 54–68%, *n* = 199) and third-generation cephalosporins 44% (95% CI 35–52%, *n* = 183).

#### Susceptibility patterns by country

Twenty-six of the 34 bacteria vs antibiotic combinations were included in subgroup analysis by country. Of these, 16 (62%) showed a significant difference by country, however 15 (94%) had significant heterogeneity within at least one of the country subgroups included. No consistent trend was observed in susceptibility proportions by country. Median heterogeneity when grouped by country was 58.33 (IQR 0–93.14), and 85.18 (IQR 49.23–93.59) when limited to at least three studies per country for the outcome.

#### Susceptibility patterns by year of publication

Meta-regression found a significant reduction in proportion of susceptible isolates by study year for *E. coli* against carbapenems (p = 0.001), and *S. aureus* against flucloxacillin (p = 0.025) (Supplemental Data 5). *E. coli* against 3 GC (p = 0.069), *Klebsiella* spp. against carbapenems (p = 0.051), and *Acinetobacter* spp. against carbapenems (p = 0.055) showed some evidence of a reduction in susceptibility with time but did not meet the significance threshold. Meanwhile *E. coli* vs ampicillin (p = 0.013) showed a significant increase in susceptibility with time.

#### Publication bias

Funnel plots and results of the Egger's regression for small-study effects are shown in Supplemental Data 1, Figures S48 and S49. Among the 29 outcomes with sufficient studies for an Egger's regression, only three outcomes showed potential risk of small study bias: *S. aureus* against vancomycin (p = 0.008); *S. agalactiae* against ampicillin; and *S. pneumoniae* against ampicillin (p = 0.006). For *S. pneumoniae*, there was no evidence of bias for meningitis (p = 0.885) and non-meningitis (p = 0.456) when considered separately.

## Discussion

This review demonstrates that AMR threatens child health in the WPRO region. It also highlights a need for more high-quality AMR surveillance data from the region, as only six of the 18 LMICs were represented by AMR data in this review, and more than two-thirds of the data was from China. Despite this limitation, this review reveals clear evidence of the non-susceptibility of important pathogens to WHO-recommended empiric antibiotics used to treat severe bacterial infections in children (see Table 1). For example, WHO recommends Access antibiotics ampicillin (or benzylpenicillin) and gentamicin as first-line empirical therapy in suspected neonatal sepsis and third-generation cephalosporins (Watch antibiotics) as second-line.^8^^,^^14^ In this review, the most common gram-negative isolates for neonatal sepsis had a susceptibility of less than 80% (and as low as 21%) to ampicillin, gentamicin, and third-generation cephalosporins, meaning current treatment regimens are unlikely to be curative for these common pathogens. A high prevalence of non-susceptibility to recommended empirical therapies has previously been described among invasive bacterial isolates throughout Africa and South Asia, with authors raising concerns about the continued use of these regimens given the low probability of treatment success.^13^^,^^71^, ^72^, ^73^, ^74^

When comparing the susceptibility profiles documented in this review to those in other parts of the globe, *E. coli* susceptibility to ampicillin was similar to previous reviews of Africa and South Asia.^13^^,^^72^^,^^74^ *E. coli* susceptibility to gentamicin was similar to isolates from Africa,^72^^,^^74^ but much higher in this study than isolates from South Asia.^13^^,^^74^ For *Klebsiella* spp., susceptibility to gentamicin was higher in this review than reported from South Asia,^13^^,^^74^ but similar to susceptibility reported from Africa.^72^^,^^74^ *E. coli* and *Klebsiella* spp. had susceptibilities to third-generation cephalosporins reported in this review that were lower than found in Africa,^72^^,^^74^ but higher than isolates identified in South Asia.^13^^,^^74^ Although *S. aureus* isolates in this review remained susceptible to vancomycin, similar to other regions,^75^ 28% of isolates were flucloxacillin resistant, comparable to resistance proportions reported in other LMICs.^75^

Due to increasing non-susceptibility to first- and second-line therapies, carbapenems are frequently prescribed as empiric therapy. In this review, half of the bacteria tested against carbapenems (4/8 combinations) had a pooled susceptibility of <90%; these were *Klebsiella* spp., *P. aeruginosa, Acinetobacter* spp., and *S. pneumoniae.* The level of reduced *S. pneumoniae* susceptibility to carbapenems was surprising. While plausible (given that it shares resistance mechanisms with other beta-lactams), the discordance with the *S. pneumoniae* susceptibility results reported for other beta-lactams highlights the data quality limitations in this review. In general, data were from low-quality evidence studies that were predominately retrospective, lacked rigorous reporting of microbiological methods and standard definitions, and used passive surveillance to identify cases. The interpretation of pooled susceptibility data in this study was also limited by observed heterogeneity in testing methods and inadequate quality control protocols. This raises concerns about the reliability and reproducibility of the reported susceptibility results, ultimately impacting the ability to draw conclusions from the pooled data.

This review had additional limitations. Only six of the 18 WPRO LMICs were represented, risking an underestimation of the burden of AMR in the region. More than two-thirds described AMR data from China. This disproportionate representation suggests selection, reporting, and geographical biases for the pooled data and impacts subsequent interpretation. Non-English language studies were excluded, which may result in further selection bias. The differentiation between community- and hospital-acquired infections was infrequently defined and therefore risks over-representing non-susceptibility proportions by biasing towards the inclusion of predominantly hospital-acquired infections, an issue consistently noted in the paediatric AMR literature.^76^ There was only one cross-sectional study, and no studies reported prevalent cases of bacterial infections for any of the organisms of interest. Finally, while the outcomes of this study provided robust susceptibility estimates with narrow confidence intervals, there was considerable heterogeneity within estimates which were unable to be reduced by subgroup stratification by year, country or clinical syndrome, highlighting the variability of susceptibilities across the studies and the difficulties in providing accurate generalisable regional estimates.

Nevertheless, this review has important implications for clinical practice and policy framework. Considering the region is home to approximately one-quarter of the world's children,^1^ the 29,236 isolates with AST information over this study period demonstrates the paucity of published data for such a substantial population at risk. This review highlights the urgent need for more rigorous and routinely collected surveillance data to improve the understanding of AMR in the region and help target treatment appropriately.^72^ Given the limited capacity of many LMIC laboratories to support clinical decision-making, there is a necessary reliance on empiric therapy based on regional and international guidelines. Unless these guidelines are revised regularly and are based on robust AMR data, clinicians are in danger of prescribing ineffective therapy that could jeopardise patient outcomes.^73^

Neonates and premature infants are particularly vulnerable amongst this cohort, as they are frequently exposed to MDR bacteria (such as *Klebsiella* spp. and *Acinetobacter* spp.) during prolonged hospital stays.^76^ While, in general, isolates included in this review were sensitive to carbapenems, the concerning proportion of bacteria that were non-susceptible to carbapenems highlights the threat of emerging resistance in the WPRO region. Few effective antibiotics have been adequately studied to treat MDR infections in the neonatal population, resulting in substantial prescribing risks of reduced efficacy or increased toxicity due to the potential for sub- or supra-therapeutic dosing.^76^ Considering the inadequate pace of development of new antimicrobials and the currently cumbersome drug regulatory framework to introduce new agents to the neonatal and paediatric population, optimising the dose, duration and formulation of currently available antimicrobials to maximise clinical efficacy–while minimising toxicity and unnecessary deaths due to AMR–is paramount.^77^ Given there are few antimicrobial resistance awareness and stewardship programmes across the region,^5^ enhanced efforts to promote antimicrobial stewardship are essential to guide rational antibiotic use and prevent the spread of AMR.

In summary, the burden of antimicrobial resistance among children within the Western Pacific region is alarming. This review suggests many WHO-recommended and commonly-prescribed antibiotic regimens are inefficacious, although the quality of laboratory data supporting this conclusion is sub-optimal and geographically biased. Enhanced efforts to strengthen local microbiological capacity, antimicrobial surveillance, and stewardship programs across the region remain vital to ensure robust data can guide improved treatment regimens and reduce the morbidity and mortality burden of infectious diseases in children across the region.

## Contributors

PCMW conceptualised the study. NM and PCMW performed the literature search. NM, EAA, BFRD, AD, and PP conducted title and abstract screen, and assessed studies for inclusion. In case of uncertainty consultation was sought with PCMW or EAA for a final decision. NM and BFRD performed data extraction. Grading was performed by NM and BFRD. PCMW performed a second direct assessment and verification of the underlying data, as well as second grading assessment. BFRD carried out meta-analysis. NM wrote the first draft of the paper. PCMW, EA, PT and BFRD reviewed and revised the final manuscript.

## Data sharing statement

Raw antimicrobial susceptibility data extracted from the included studies are available in Supplementary Data 2.

## Editor note

The Lancet Group takes a neutral position with respect to territorial claims in published maps and institutional affiliations.

## Declaration of interests

PW received support to attend conference via ECCMID to present on the topic of antimicrobial resistance in neonatal sepsis; requested testimony to report on a Serratia outbreak in a neonatal intensive care unit. Other coauthors have nothing to declare.
