# Supplementary material for: Antimicrobial susceptibility profiles of invasive bacterial infections among children from low- and middle-income countries in the Western Pacific Region (WPRO) – a systematic review and meta-analysis
Source: Lancet Reg Health West Pac. 2024 Aug 31;51:101177. doi: 10.1016/j.lanwpc.2024.101177 (PMC11402324; doi:10.1016/j.lanwpc.2024.101177)

Supplement 1. PICOS domains and Search Strategy

PICO:
P (population/problem): Children (≤18 years of age) with culture positive bacterial infection of otherwise sterile sites.
I (intervention): Antibiotic treatment (WHO-empiric regimes plus carbapenem)
C (comparison): Not applicable
O (outcome): Pathogens causing invasive bacterial disease in children and the antimicrobial susceptibility pattern
S (setting): LMIC in WPRO region

REGION - WPRO:
Included countries (excluding WB-defined HICs): Cambodia, China, Fiji, Kiribati , Laos PDR, Malaysia, Marshall Islands, Micronesia, Mongolia, Niue, PNG, Philippines, Solomon Islands, Samoa, Tuvalu, Tonga, Vanuatu, Vietnam.

Search strategy and selection criteria

References for the review were identified through searches of Embase, Global Health, Pubmed, and the Cochrane Database of Systematic Reviews, using search terms "antimicrobial", "antibiotic", "susceptible", "sensitive", "paediatric", "child", "neonate" and "infant", in combination with 18 low- or middle-income countries within the Western Pacific Region: "Cambodia"; "China"; "Fiji"; "Kiribati"; "Laos People's Democratic Republic"; "Malaysia"; "Marshall Islands"; "Micronesia"; "Mongolia"; "Niue"; "Papua New Guinea"; "Philippines"; "Samoa"; "Solomon Islands"; "Tonga"; "Tuvalu"; "Vanuatu"; and "Vietnam". The search was limited to studies published between 1 January 2011 and 26 March 2021. Only papers published in English were reviewed. The final reference list was generated on the basis of relevance to the pre-determined inclusion and exclusion criteria (Table 1).

Databases: Pubmed, Cochrane, Embase, Global Health + Grey literature search
Search Terms:
(((antimicrobial[Text Word] OR antibiotic, resistan*[Text Word]) AND (antimicrobial[Text Word] OR antibiotic, susceptib*[Text Word] OR sensitiv*[Text Word])) AND (pediatr*[Text Word] OR paediatr*[Text Word] OR child*[Text Word] OR neonat*[Text Word] OR infant*[Text Word])) AND (cambodia[MeSH] OR china[MeSH] OR fiji[MeSH] OR kiribati [MeSH] OR laos[MeSH] OR malaysia[MeSH] OR marshall islands [MeSH] OR micronesia[MeSH] OR mongolia[MeSH] OR nui[MeSH] OR papua new guinea[MeSH] OR philippines[MeSH] OR solomon islands [MeSH] OR samoa[MeSH] OR tuvalu [MeSH] OR tonga[MeSH] OR vanuatu[MeSH] OR vietnam[MeSH] OR cambodia[Text Word] OR china[Text Word] OR fiji[Text Word] OR kiribati[Text Word] OR laos[Text Word] OR lao pdr[Text Word] OR lao people's democratic republic[Text Word] OR malaysia[Text Word] OR marshall islands[Text Word] OR micronesia[Text Word] OR mongolia[Text Word] OR papua new guinea[Text Word] OR philippines[Text Word] OR philipines[Text Word] OR phillipines[Text Word] OR phillippines[Text Word] OR samoa[Text Word] OR solomon island[Text Word] OR solomon islands[Text Word] OR tonga[Text Word] OR tuvalu [Text Word] OR vanuatu[Text Word] OR vietnam[Text Word] OR viet nam[Text Word] cambodian[Text Word] OR cambodians[Text Word] OR chinese[Text Word] OR fijian[Text Word] OR fijians[Text Word] OR lao[Text Word] OR laotian[Text Word] OR laotians[Text

Word] OR malaysian[Text Word] OR malaysians[Text Word] OR marshallese[Text Word] OR micronesian[Text Word] OR micronesians[Text Word] OR mongolian[Text Word] OR mongolians[Text Word] OR mongol[Text Word] OR papua new guinean[Text Word] OR papua new guineans[Text Word] OR philippine[Text Word] OR philippines[Text Word] OR philipine[Text Word] OR philipines[Text Word] OR phillipine[Text Word] OR phillipines[Text Word] OR phillippine[Text Word] OR phillippines[Text Word] OR filipino[Text Word] OR filipinos[Text Word] OR filipina[Text Word] OR filipinas[Text Word] OR samoan[Text Word] OR samoans[Text Word] OR solomon islander[Text Word] OR solomon islanders[Text Word] OR tongan[Text Word] OR tongans[Text Word] OR tuvaluan[Text Word] OR tuvaluans[Text Word] OR vanuatu[Text Word] OR vanuatuan[Text Word] OR vanuatuans[Text Word] OR vietnamese[Text Word])

Limits:

- Last 10 years
- Humans only

Pre-defined inclusion criteria:

- Research pertaining to bacterial infections: incidence, prevalence, aetiology, clinical infections
- Isolates from sterile sites only, plus urine (if pertaining to clinical urinary tract infections) and stool (if pertaining to *Shigella* or *Salmonella* spp. clinical infections)
- Specified paediatric data (age up to and including 18 years)
- Antimicrobial testing methods documented and in line with CLSI/EUCAST recommendations
- Published within the last 10 years
- Bacterial GLASS 2020 organisms
    - *Escherichia coli*
    - *Klebsiella pneumoniae*
    - *Acinetobacter* spp.
    - *Staphylococcus aureus*
    - *Streptococcus pneumoniae*
    - *Salmonella* spp.
    - *Shigella* spp.
    - *N. gonorrhoeae*
    - *Pseudomonas aeruginosa*

    PLUS other bacterial pathogens particularly relevant in children:
    - *Streptococcus agalactiae*
    - *Streptococcus pyogenes*
    - *Haemophilus influenzae*
    - *Neisseria meningitidis*

Pre-defined exclusion criteria:

- Data aggregated with adult data
- Data aggregated with other regions outside the pre-defined geographic area
- Isolates pertaining to carriage or colonisation studies
- Small retrospective case series where n<10
- Literature focussed on high-risk populations only (children LWHIV, profoundly immunosuppressed populations, children with SAM)
- Poorly defined laboratory procedures, as defined by the MICRO framework

S1. Figure 1. Proportion of Salmonella spp. isolates susceptibile to key antimicrobials

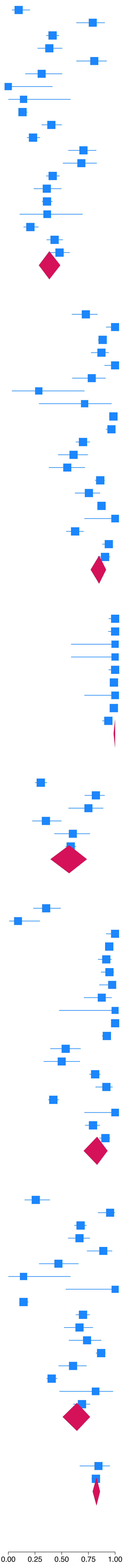

Study	Number Susceptible	Number Tested	Proportion with binomial exact 95% CI	Weight (%)
**Ampicillin**				
Yu F et al. 2011	6	62	0.10 [0.04, 0.20]	1.10
Mohan et al. 2013	34	43	0.79 [0.64, 0.90]	1.08
Li Y et al. 2014	128	309	0.41 [0.36, 0.47]	1.13
Thompson et al. 2015	30	78	0.38 [0.28, 0.50]	1.11
Nor Azizah et al. 2016	29	36	0.81 [0.64, 0.92]	1.07
Zhu et al. 2016	10	32	0.31 [0.16, 0.50]	1.07
Jiang H et al. 2017	0	7	0.00 [0.00, 0.41]	0.87
Li C et al. 2018	1	7	0.14 [0.00, 0.58]	0.87
Fox-Lewis et al. 2018	43	323	0.13 [0.10, 0.18]	1.13
Xu et al. 2018	48	119	0.40 [0.31, 0.50]	1.12
Liang et al. 2019	51	220	0.23 [0.18, 0.29]	1.13
Yang et al. 2019	38	54	0.70 [0.56, 0.82]	1.10
Yang et al. 2019	26	38	0.68 [0.51, 0.82]	1.08
Wang C et al. 2020	114	274	0.42 [0.36, 0.48]	1.13
Yue et al. 2020	22	61	0.36 [0.24, 0.49]	1.10
Duong et al. 2020	163	450	0.36 [0.32, 0.41]	1.14
Ke et al. 2020	4	11	0.36 [0.11, 0.69]	0.95
Ke et al. 2020	32	155	0.21 [0.15, 0.28]	1.13
Qiu et al. 2020	81	187	0.43 [0.36, 0.51]	1.13
Song et al. 2022	60	125	0.48 [0.39, 0.57]	1.12
Heterogeneity: τ^2 = 0.19, I^2 = 95.92%, H^2 = 24.49			0.38 [0.29, 0.49]	
Test of $\theta_i = \theta_j$: Q(19) = 288.27, p = 0.00				
**3rd generation cephalosporins**				
Yu F et al. 2011	45	62	0.73 [0.60, 0.83]	1.10
Mohan et al. 2013	43	43	1.00 [0.92, 1.00]	1.08
Li Y et al. 2014	273	309	0.88 [0.84, 0.92]	1.13
Thompson et al. 2015	68	78	0.87 [0.78, 0.94]	1.11
Nor Azizah et al. 2016	36	36	1.00 [0.90, 1.00]	1.07
Zhu et al. 2016	25	32	0.78 [0.60, 0.91]	1.07
Jiang H et al. 2017	2	7	0.29 [0.04, 0.71]	0.87
Li C et al. 2018	5	7	0.71 [0.29, 0.96]	0.87
Fox-Lewis et al. 2018	250	254	0.98 [0.96, 1.00]	1.13
Xu et al. 2018	115	119	0.97 [0.92, 0.99]	1.12
Liang et al. 2019	154	220	0.70 [0.63, 0.76]	1.13
Yang et al. 2019	33	54	0.61 [0.47, 0.74]	1.10
Yang et al. 2019	21	38	0.55 [0.38, 0.71]	1.08
Wang C et al. 2020	236	274	0.86 [0.81, 0.90]	1.13
Yue et al. 2020	46	61	0.75 [0.63, 0.86]	1.10
Duong et al. 2020	393	450	0.87 [0.84, 0.90]	1.14
Ke et al. 2020	11	11	1.00 [0.72, 1.00]	0.95
Ke et al. 2020	97	155	0.63 [0.54, 0.70]	1.13
Qiu et al. 2020	112	119	0.94 [0.88, 0.98]	1.12
Song et al. 2022	115	127	0.91 [0.84, 0.95]	1.12
Heterogeneity: τ^2 = 0.17, I^2 = 95.28%, H^2 = 21.20			0.85 [0.77, 0.92]	
Test of $\theta_i = \theta_j$: Q(19) = 270.19, p = 0.00				
**Carbapenems**				
Yu F et al. 2011	62	62	1.00 [0.94, 1.00]	1.10
Xiao et al. 2015	57	57	1.00 [0.94, 1.00]	1.10
Jiang H et al. 2017	7	7	1.00 [0.59, 1.00]	0.87
Li C et al. 2018	7	7	1.00 [0.59, 1.00]	0.87
Yue et al. 2020	61	61	1.00 [0.94, 1.00]	1.10
Duong et al. 2020	445	450	0.99 [0.97, 1.00]	1.14
Ke et al. 2020	11	11	1.00 [0.72, 1.00]	0.95
Ke et al. 2020	153	155	0.99 [0.95, 1.00]	1.13
Qiu et al. 2020	133	142	0.94 [0.88, 0.97]	1.12
Heterogeneity: τ^2 = 0.01, I^2 = 51.22%, H^2 = 2.05			1.00 [0.99, 1.00]	
Test of $\theta_i = \theta_j$: Q(8) = 13.71, p = 0.09				
**Nalidixic acid**				
Li Y et al. 2014	94	309	0.30 [0.25, 0.36]	1.13
Thompson et al. 2015	64	78	0.82 [0.72, 0.90]	1.11
Zhu et al. 2016	24	32	0.75 [0.57, 0.89]	1.07
Yang et al. 2019	19	54	0.35 [0.23, 0.49]	1.10
Yang et al. 2019	23	38	0.61 [0.43, 0.76]	1.08
Duong et al. 2020	263	450	0.58 [0.54, 0.63]	1.14
Heterogeneity: τ^2 = 0.17, I^2 = 95.69%, H^2 = 23.21			0.57 [0.39, 0.74]	
Test of $\theta_i = \theta_j$: Q(5) = 114.08, p = 0.00				
**Ciprofloxacin**				
Yu F et al. 2011	22	62	0.35 [0.24, 0.49]	1.10
Chheng et al. 2013	2	22	0.09 [0.01, 0.29]	1.03
Mohan et al. 2013	43	43	1.00 [0.92, 1.00]	1.08
Li Y et al. 2014	292	309	0.94 [0.91, 0.97]	1.13
Xiao et al. 2015	88	96	0.92 [0.84, 0.96]	1.12
Thompson et al. 2015	72	76	0.95 [0.87, 0.99]	1.11
Nor Azizah et al. 2016	35	36	0.97 [0.85, 1.00]	1.07
Zhu et al. 2016	28	32	0.87 [0.71, 0.96]	1.07
Li C et al. 2018	5	5	1.00 [0.48, 1.00]	0.80
Xu et al. 2018	119	119	1.00 [0.97, 1.00]	1.12
Liang et al. 2019	203	220	0.92 [0.88, 0.95]	1.13
Yang et al. 2019	29	54	0.54 [0.40, 0.67]	1.10
Yang et al. 2019	19	38	0.50 [0.33, 0.67]	1.08
Wang C et al. 2020	223	274	0.81 [0.76, 0.86]	1.13
Yue et al. 2020	56	61	0.92 [0.82, 0.97]	1.10
Duong et al. 2020	190	450	0.42 [0.38, 0.47]	1.14
Ke et al. 2020	11	11	1.00 [0.72, 1.00]	0.95
Ke et al. 2020	123	155	0.79 [0.72, 0.85]	1.13
Qiu et al. 2020	142	156	0.91 [0.85, 0.95]	1.13
Heterogeneity: τ^2 = 0.39, I^2 = 97.75%, H^2 = 44.47			0.83 [0.70, 0.93]	
Test of $\theta_i = \theta_j$: Q(18) = 693.39, p = 0.00				
**Cotrimoxazole**				
Yu F et al. 2011	16	62	0.26 [0.16, 0.38]	1.10
Mohan et al. 2013	41	43	0.95 [0.84, 0.99]	1.08
Li Y et al. 2014	209	309	0.68 [0.62, 0.73]	1.13
Xiao et al. 2015	64	96	0.67 [0.56, 0.76]	1.12
Nor Azizah et al. 2016	32	36	0.89 [0.74, 0.97]	1.07
Zhu et al. 2016	15	32	0.47 [0.29, 0.65]	1.07
Jiang H et al. 2017	1	7	0.14 [0.00, 0.58]	0.87
Li C et al. 2018	6	6	1.00 [0.54, 1.00]	0.84
Fox-Lewis et al. 2018	46	323	0.14 [0.11, 0.19]	1.13
Liang et al. 2019	154	220	0.70 [0.63, 0.76]	1.13
Yang et al. 2019	36	54	0.67 [0.53, 0.79]	1.10
Yang et al. 2019	28	38	0.74 [0.57, 0.87]	1.08
Wang C et al. 2020	238	274	0.87 [0.82, 0.91]	1.13
Yue et al. 2020	37	61	0.61 [0.47, 0.73]	1.10
Duong et al. 2020	183	450	0.41 [0.36, 0.45]	1.14
Ke et al. 2020	9	11	0.82 [0.48, 0.98]	0.95
Ke et al. 2020	107	155	0.69 [0.61, 0.76]	1.13
Heterogeneity: τ^2 = 0.28, I^2 = 97.07%, H^2 = 34.18			0.64 [0.51, 0.77]	
Test of $\theta_i = \theta_j$: Q(16) = 609.72, p = 0.00				
**Azithromycin**				
Zhu et al. 2016	27	32	0.84 [0.67, 0.95]	1.07
Duong et al. 2020	369	450	0.82 [0.78, 0.85]	1.14
Heterogeneity: τ^2 = 0.00, I^2 = 0.00%, H^2 = 1.00			0.83 [0.79, 0.86]	
Test of $\theta_i = \theta_j$: Q(1) = 0.04, p = 0.84				
Test of group differences: Q_b(6) = 212.70, p = 0.00				

Random-effects REML model
Sorted by: year_pub

S1. Figure 2. Proportion of P. aeruginosa isolates susceptibile to key antimicrobials

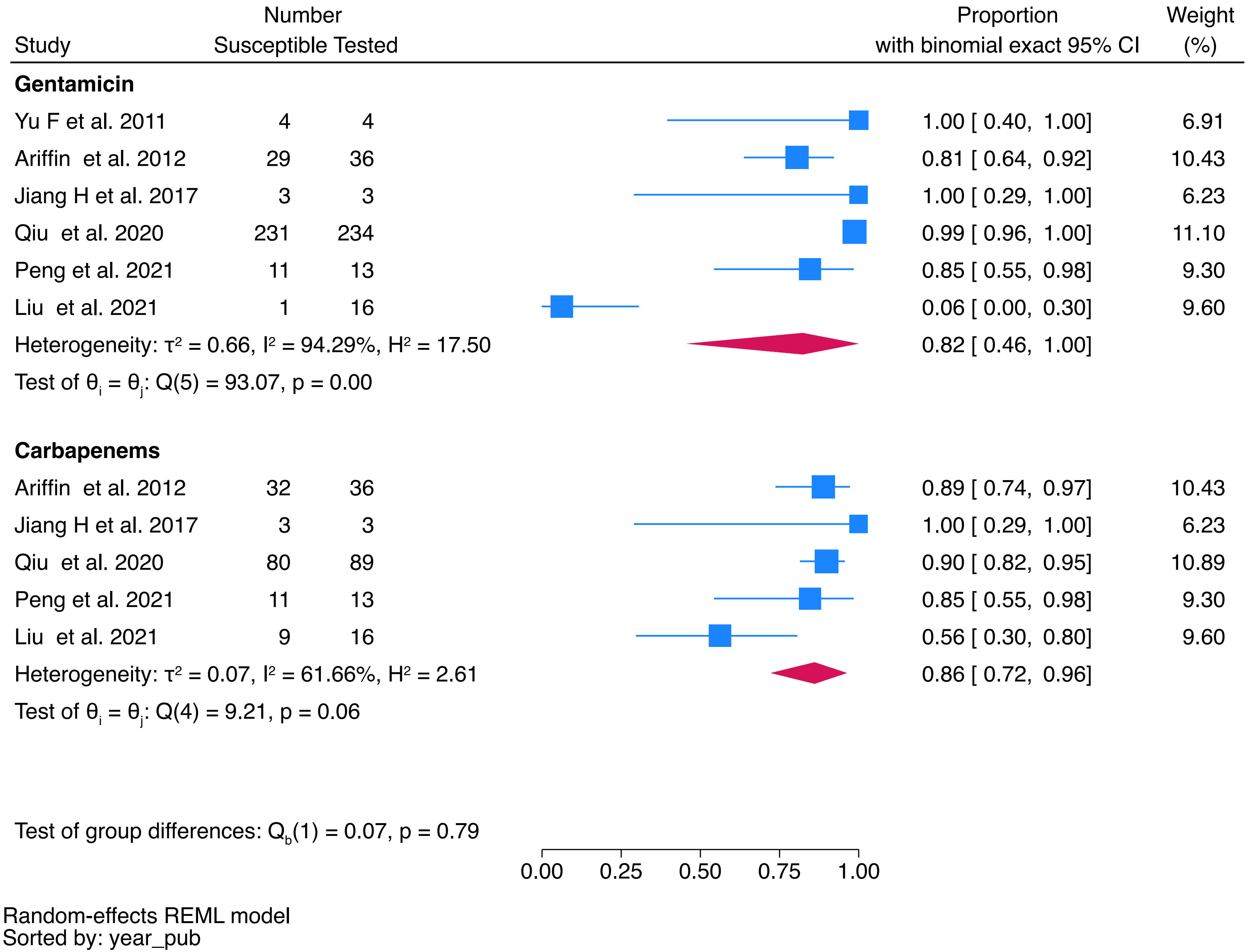

S1
Figure 3

Proportion of Acinetobacter spp. isolates susceptibile to key antimicrobials

Study	Number Susceptible	Tested	Proportion with binomial exact 95% CI	Weight (%)
**Gentamicin**				
Yu F et al. 2011	1	1	1.00 [0.03, 1.00]	1.00
Ariffin et al. 2012	26	29	0.90 [0.73, 0.98]	10.81
Jiang H et al. 2017	1	2	0.50 [0.01, 0.99]	1.62
Qiu et al. 2020	85	135	0.63 [0.54, 0.71]	18.32
Peng et al. 2021	14	19	0.74 [0.49, 0.91]	8.51
Liu et al. 2021	30	37	0.81 [0.65, 0.92]	12.17
Heterogeneity: $\tau^2 = 0.05$, $I^2 = 57.16\%$, $H^2 = 2.33$			0.81 [0.66, 0.93]	
Test of $\theta_i = \theta_j$: $Q(5) = 12.25$, $p = 0.03$				
**Carbapenems**				
Ariffin et al. 2012	24	29	0.83 [0.64, 0.94]	10.81
Jiang H et al. 2017	2	2	1.00 [0.16, 1.00]	1.62
Qiu et al. 2020	66	97	0.68 [0.58, 0.77]	17.04
Peng et al. 2021	5	11	0.45 [0.17, 0.77]	5.93
Liu et al. 2021	25	37	0.68 [0.50, 0.82]	12.17
Heterogeneity: $\tau^2 = 0.00$, $I^2 = 11.65\%$, $H^2 = 1.13$			0.72 [0.63, 0.80]	
Test of $\theta_i = \theta_j$: $Q(4) = 6.09$, $p = 0.19$				

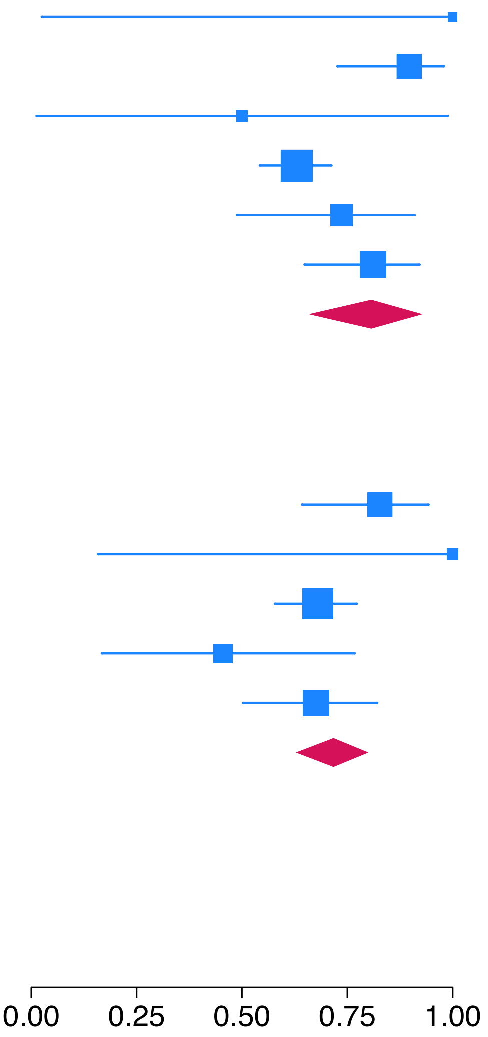

Test of group differences: $Q_b(1) = 0.62$, $p = 0.43$

Random-effects REML model
Sorted by: year_pub

S1. Figure 4. Proportion of H. influenzae isolates susceptibile to key antimicrobials

Study	Number Susceptible	Number Tested	Proportion with binomial exact 95% CI	Weight (%)
**Ampicillin**				
Nor Azizah et al. 2016	8	8	1.00 [0.63, 1.00]	8.98
Jiang H et al. 2017	1	17	0.06 [0.00, 0.29]	9.92
Li C et al. 2018	4	4	1.00 [0.40, 1.00]	7.71
Fox-Lewis et al. 2018	26	56	0.46 [0.33, 0.60]	10.65
Heterogeneity: $\tau^2 = 1.01$, $I^2 = 93.89\%$, $H^2 = 16.38$			0.65 [0.12, 1.00]	
Test of $\theta_i = \theta_j$: Q(3) = 35.53, p = 0.00				
**3rd generation cephalosporins**				
Jiang H et al. 2017	16	17	0.94 [0.71, 1.00]	9.92
Li C et al. 2018	6	6	1.00 [0.54, 1.00]	8.50
Fox-Lewis et al. 2018	54	57	0.95 [0.85, 0.99]	10.66
Heterogeneity: $\tau^2 = 0.00$, $I^2 = 0.00\%$, $H^2 = 1.00$			0.97 [0.90, 1.00]	
Test of $\theta_i = \theta_j$: Q(2) = 0.19, p = 0.91				
**Carbapenems**				
Jiang H et al. 2017	17	17	1.00 [0.80, 1.00]	9.92
Li C et al. 2018	8	8	1.00 [0.63, 1.00]	8.98
Heterogeneity: $\tau^2 = 0.00$, $I^2 = 0.00\%$, $H^2 = 1.00$			1.00 [0.93, 1.00]	
Test of $\theta_i = \theta_j$: Q(1) = 0.06, p = 0.81				
**Chloramphenicol**				
Jiang H et al. 2017	17	17	1.00 [0.80, 1.00]	9.92
Li C et al. 2018	1	1	1.00 [0.03, 1.00]	4.82
Heterogeneity: $\tau^2 = 0.00$, $I^2 = 0.00\%$, $H^2 = 1.00$			1.00 [0.99, 1.00]	
Test of $\theta_i = \theta_j$: Q(1) = 0.41, p = 0.52				

Test of group differences: $Q_b(3) = 4.20$, p = 0.24

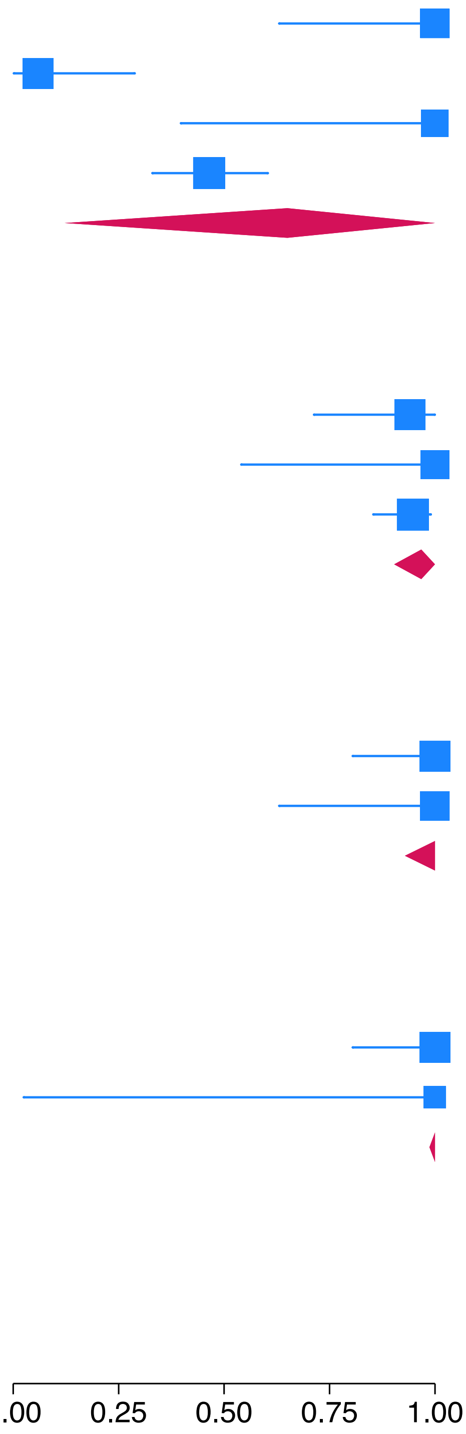

Random-effects REML model
Sorted by: year_pub

S1. Figure 5. Proportion of Group B Streptococcus isolates susceptibile to key antimicrobials

Study	Number Susceptible	Number Tested	Proportion with binomial exact 95% CI	Weight (%)
**Ampicillin**				
Wang P et al. 2015	40	40	1.00 [0.91, 1.00]	3.28
Nor Azizah et al. 2016	11	11	1.00 [0.72, 1.00]	0.93
Guo et al. 2016	14	14	1.00 [0.77, 1.00]	1.17
Dong Y et al. 2017	25	25	1.00 [0.86, 1.00]	2.06
Jiang H et al. 2017	13	13	1.00 [0.75, 1.00]	1.09
Li C et al. 2018	17	17	1.00 [0.80, 1.00]	1.42
Shen et al. 2019	9	9	1.00 [0.66, 1.00]	0.77
Li J et al. 2019	93	93	1.00 [0.96, 1.00]	7.56
Li X et al. 2019	20	20	1.00 [0.83, 1.00]	1.66
Qiu et al. 2020	182	182	1.00 [0.98, 1.00]	14.76
Peng et al. 2021	26	27	0.96 [0.81, 1.00]	2.22
Tan et al. 2022	148	148	1.00 [0.98, 1.00]	12.01
Heterogeneity: $\tau^2 = 0.00$, $I^2 = 0.00\%$, $H^2 = 1.00$			1.00 [1.00, 1.00]	
Test of $\theta_i = \theta_j$: Q(11) = 5.66, p = 0.90				
**3rd generation cephalosporins**				
Wang P et al. 2015	40	40	1.00 [0.91, 1.00]	3.28
Guo et al. 2016	10	10	1.00 [0.69, 1.00]	0.85
Dong Y et al. 2017	24	25	0.96 [0.80, 1.00]	2.06
Jiang H et al. 2017	13	13	1.00 [0.75, 1.00]	1.09
Li C et al. 2018	19	19	1.00 [0.82, 1.00]	1.58
Li X et al. 2019	19	20	0.95 [0.75, 1.00]	1.66
Qiu et al. 2020	74	74	1.00 [0.95, 1.00]	6.03
Heterogeneity: $\tau^2 = 0.00$, $I^2 = 4.02\%$, $H^2 = 1.04$			1.00 [0.98, 1.00]	
Test of $\theta_i = \theta_j$: Q(6) = 4.71, p = 0.58				
**Carbapenems**				
Dong Y et al. 2017	25	25	1.00 [0.86, 1.00]	2.06
Jiang H et al. 2017	13	13	1.00 [0.75, 1.00]	1.09
Li C et al. 2018	9	9	1.00 [0.66, 1.00]	0.77
Qiu et al. 2020	33	33	1.00 [0.89, 1.00]	2.71
Heterogeneity: $\tau^2 = 0.00$, $I^2 = 0.00\%$, $H^2 = 1.00$			1.00 [0.98, 1.00]	
Test of $\theta_i = \theta_j$: Q(3) = 0.22, p = 0.97				
**Vancomycin**				
Guo et al. 2016	14	14	1.00 [0.77, 1.00]	1.17
Dong Y et al. 2017	25	25	1.00 [0.86, 1.00]	2.06
Jiang H et al. 2017	13	13	1.00 [0.75, 1.00]	1.09
Li C et al. 2018	29	29	1.00 [0.88, 1.00]	2.39
Shen et al. 2019	9	9	1.00 [0.66, 1.00]	0.77
Li X et al. 2019	20	20	1.00 [0.83, 1.00]	1.66
Qiu et al. 2020	181	181	1.00 [0.98, 1.00]	14.68
Peng et al. 2021	50	50	1.00 [0.93, 1.00]	4.08
Heterogeneity: $\tau^2 = 0.00$, $I^2 = 0.00\%$, $H^2 = 1.00$			1.00 [1.00, 1.00]	
Test of $\theta_i = \theta_j$: Q(7) = 1.80, p = 0.97				

Test of group differences: $Q_b(3) = 2.23$, p = 0.53

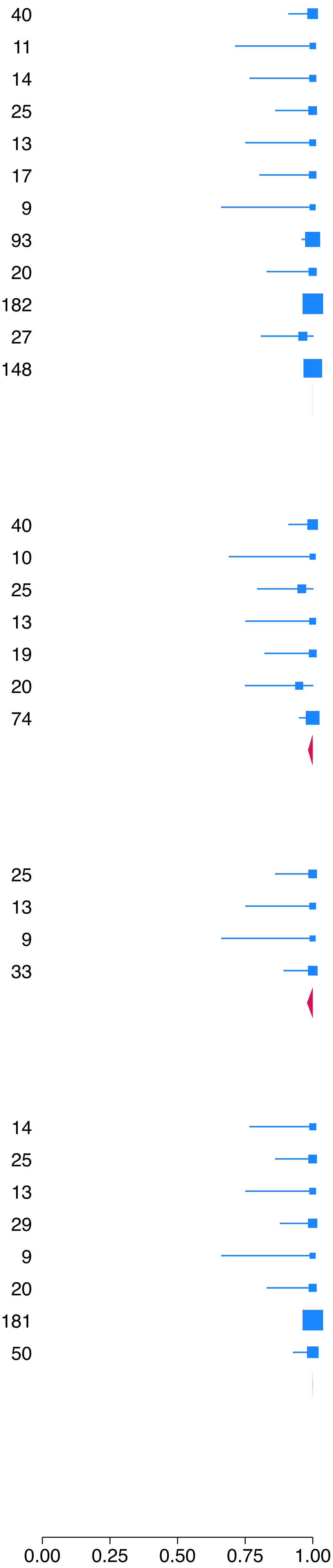

Random-effects REML model
Sorted by: year_pub

S1 Figure 6 Proportion of E. coli isolates susceptibile to Ampicillin by subgroup

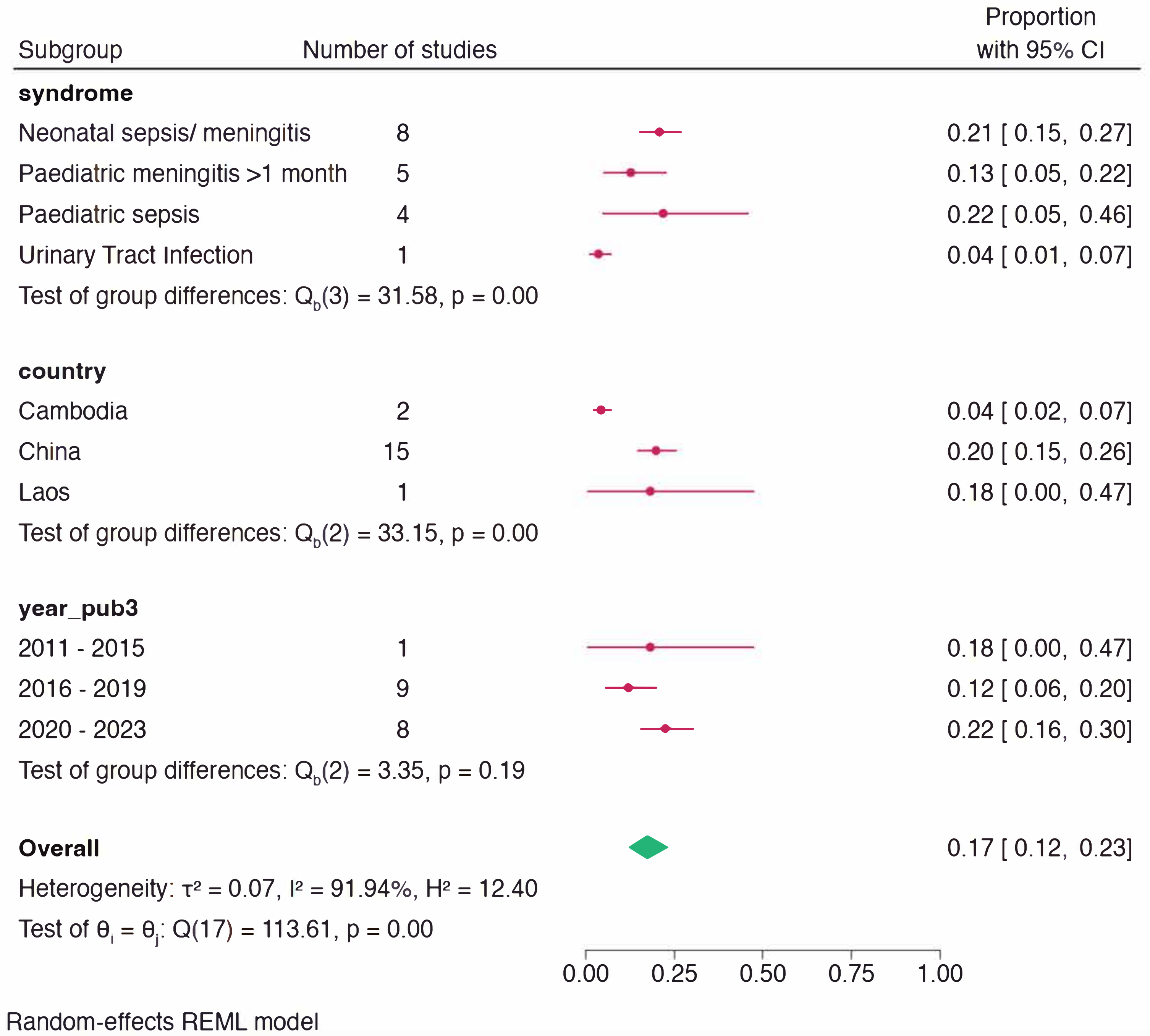

S1 Figure 7 Proportion of E. coli isolates susceptibile to Gentamicin by subgroup

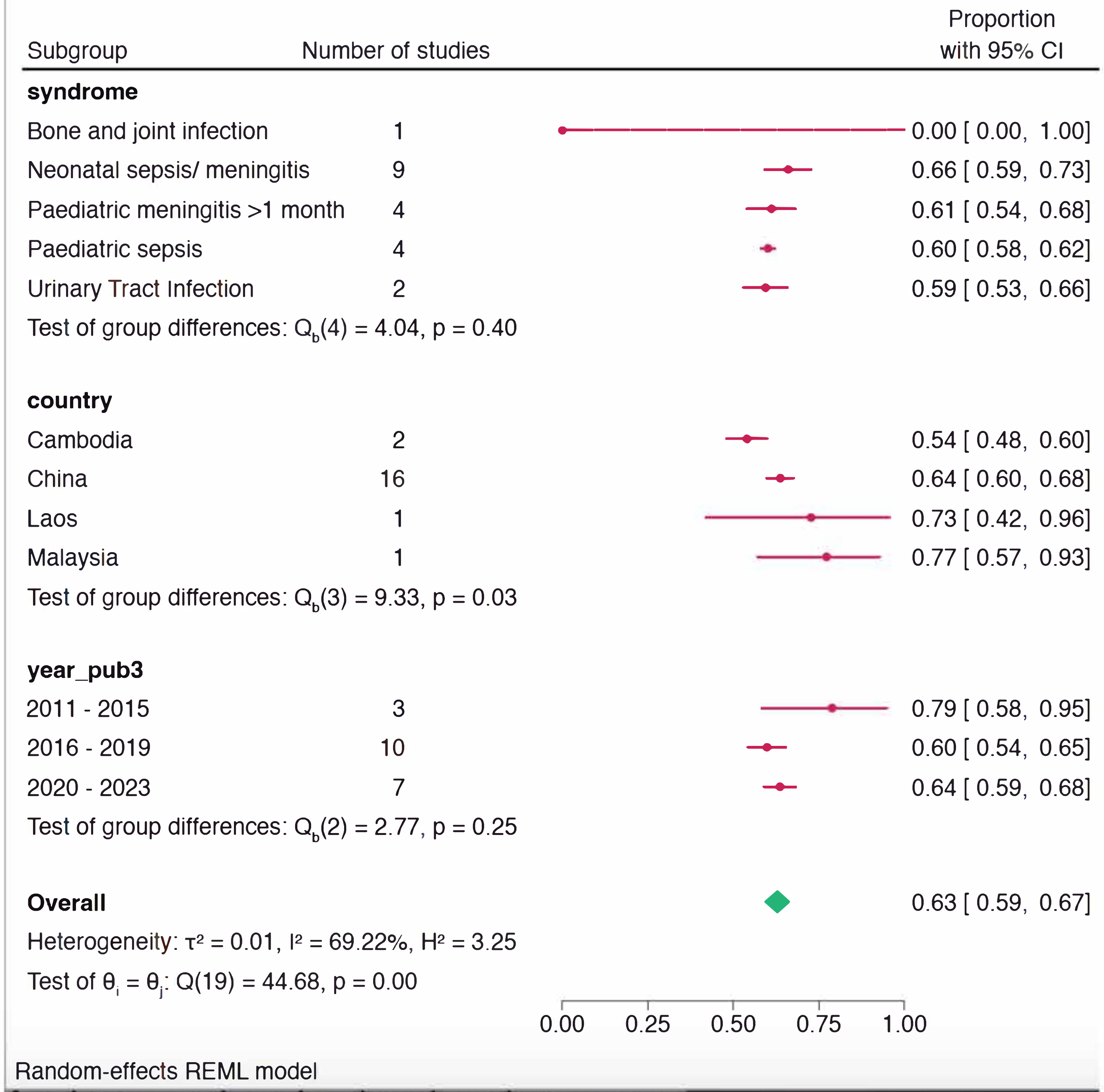

S1 Figure 8 Proportion of E. coli isolates susceptibile to 3rd Generation Cephalosporins by subgroup

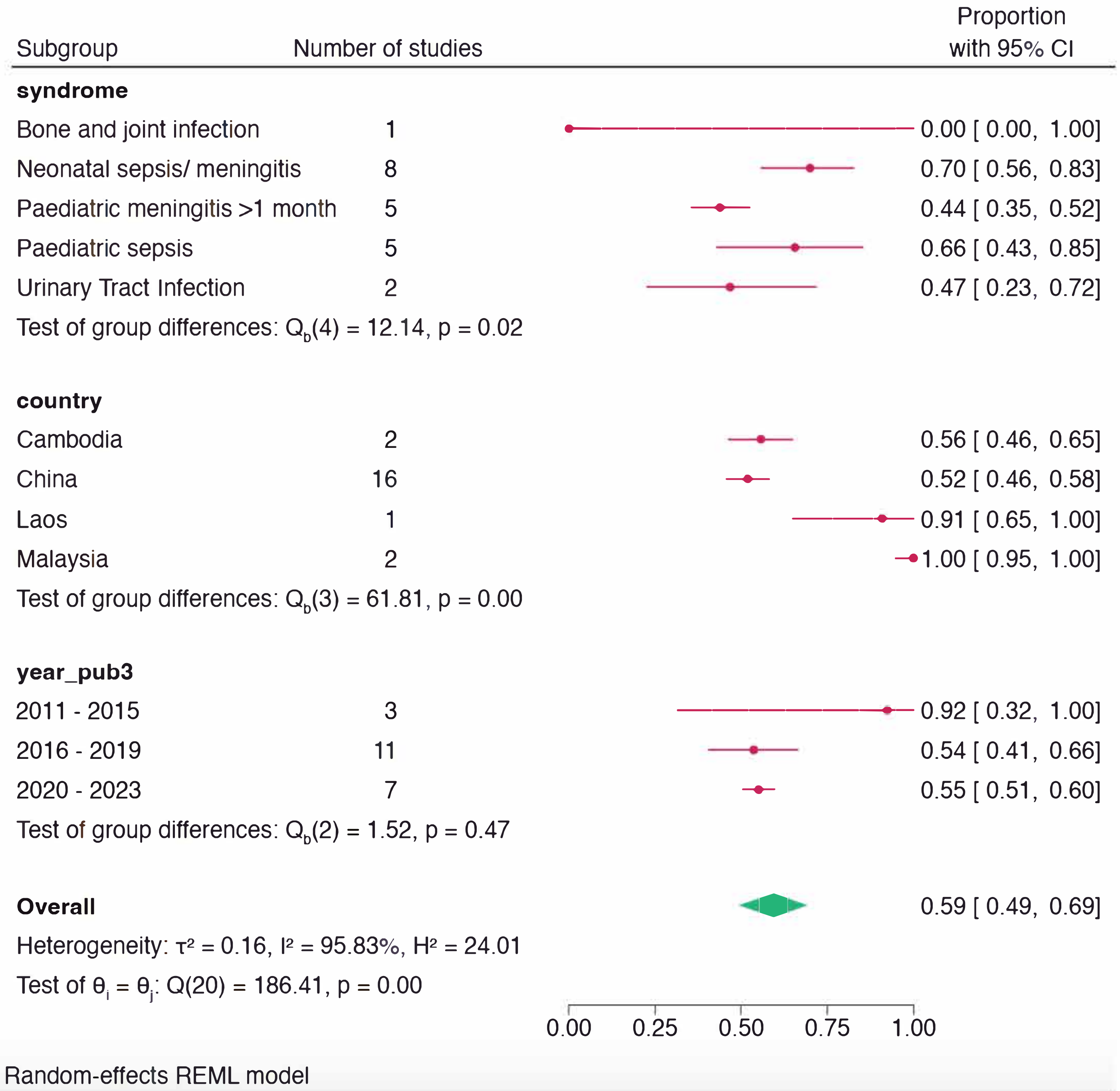

S1 Figure 9 Proportion of E. coli isolates susceptibile to Carbapenems by subgroup

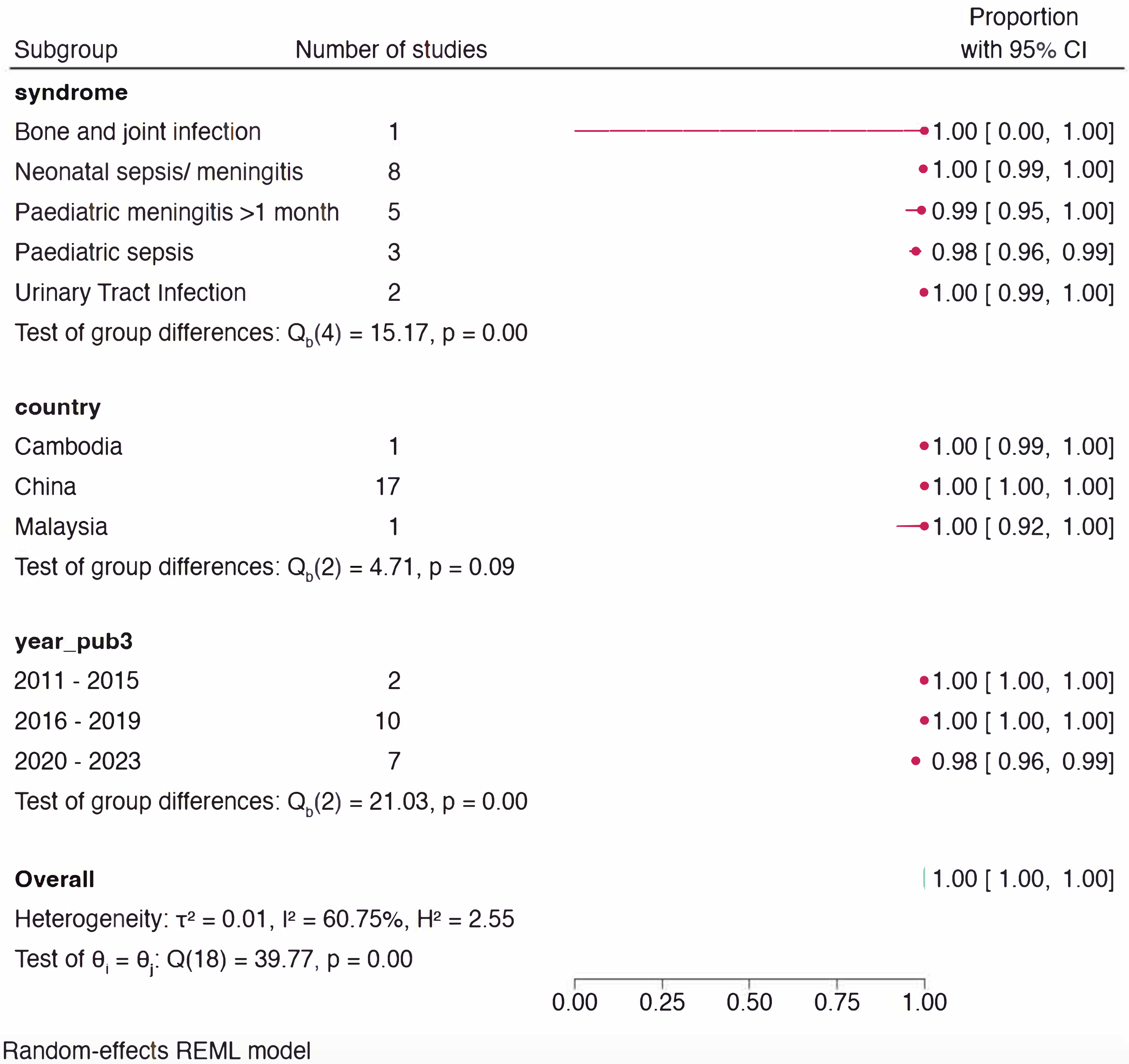

SI Figure 10

Org	Ab	subgroup	df	q	pvalue	tau	i2	h2	Overall_Tau2	Overall_I2
E. coli	Ampicillin	Cambodia	1	0.7	0.402	0	0	1	0.074	91.94
E. coli	Ampicillin	China	14	58.94	0	0.049	88.81	8.94	0.074	91.94
E. coli	Ampicillin	Laos	0	0	.	0	.	.	0.074	91.94
E. coli	Ampicillin	Neonatal sep	7	24.65	0.001	0.024	69.27	3.25	0.074	91.94
E. coli	Ampicillin	Paediatric m	4	8.26	0.083	0.036	54.19	2.18	0.074	91.94
E. coli	Ampicillin	Paediatric s-	3	35.59	0	0.255	98.99	99.08	0.074	91.94
E. coli	Ampicillin	Urinary Trac-	0	0	.	0	.	.	0.074	91.94
E. coli	Ampicillin	2011	0	0	.	0	.	.	0.074	91.94
E. coli	Ampicillin	2016	8	46.23	0	0.07	81.19	5.32	0.074	91.94
E. coli	Ampicillin	2020	7	39.77	0	0.054	93.49	15.37	0.074	91.94
E. coli	gent	Cambodia	1	0.3	0.582	0	0	1	0.013	69.22
E. coli	gent	China	15	35.14	0.002	0.011	68.7	3.19	0.013	69.22
E. coli	gent	Laos	0	0	.	0	.	.	0.013	69.22
E. coli	gent	Malaysia	0	0	.	0	.	.	0.013	69.22
E. coli	gent	Bone and joi	0	0	.	0	.	.	0.013	69.22
E. coli	gent	Neonatal sep	8	26.51	0.001	0.027	68.93	3.22	0.013	69.22
E. coli	gent	Paediatric m	3	0.36	0.949	0	0	1	0.013	69.22
E. coli	gent	Paediatric s-	3	4.7	0.195	0	0.01	1	0.013	69.22
E. coli	gent	Urinary Trac-	1	2.56	0.11	0.006	60.87	2.56	0.013	69.22
E. coli	gent	2011	2	2.52	0.284	0	0	1	0.013	69.22
E. coli	gent	2016	9	19.02	0.025	0.015	59.54	2.47	0.013	69.22
E. coli	gent	2020	6	20.49	0.002	0.011	76.89	4.33	0.013	69.22
E. coli	3gc	Cambodia	1	2.4	0.121	0.011	58.38	2.4	0.156	95.83
E. coli	3gc	China	15	102.57	0	0.034	85.18	6.75	0.156	95.83
E. coli	3gc	Laos	0	0	.	0	.	.	0.156	95.83
E. coli	3gc	Malaysia	1	0.02	0.879	0	0	1	0.156	95.83
E. coli	3gc	Bone and joi	0	0	.	0	.	.	0.156	95.83
E. coli	3gc	Neonatal sep	7	39.42	0	0.143	91.35	11.56	0.156	95.83
E. coli	3gc	Paediatric m	4	4.41	0.353	0.005	13.59	1.16	0.156	95.83
E. coli	3gc	Paediatric s-	4	27.5	0	0.237	98.35	60.59	0.156	95.83
E. coli	3gc	Urinary Trac-	1	36.8	0	0.132	97.28	36.8	0.156	95.83
E. coli	3gc	2011	2	7.54	0.023	0.599	83.94	6.22	0.156	95.83
E. coli	3gc	2016	10	97.26	0	0.149	92.35	13.07	0.156	95.83
E. coli	3gc	2020	6	15.84	0.015	0.008	68.08	3.13	0.156	95.83
E. coli	carb	Cambodia	0	0	.	0	.	.	0.012	60.75
E. coli	carb	China	16	31.1	0.013	0.01	58.28	2.4	0.012	60.75
E. coli	carb	Malaysia	0	0	.	0	.	.	0.012	60.75
E. coli	carb	Bone and joi	0	0	.	0	.	.	0.012	60.75
E. coli	carb	Neonatal sep	7	6.49	0.484	0.004	22.35	1.29	0.012	60.75
E. coli	carb	Paediatric m	4	6	0.199	0.022	40.24	1.67	0.012	60.75
E. coli	carb	Paediatric s-	2	3.46	0.178	0.002	48.39	1.94	0.012	60.75
E. coli	carb	Urinary Trac-	1	0.67	0.412	0	0.01	1	0.012	60.75
E. coli	carb	2011	1	0.47	0.495	0	0	1	0.012	60.75
E. coli	carb	2016	9	1.88	0.993	0	0	1	0.012	60.75
E. coli	carb	2020	6	9.99	0.125	0.002	31.91	1.47	0.012	60.75

S1 Figure 11 Proportion of Klebsiella spp. isolates susceptibile to gentamicin by subgroup

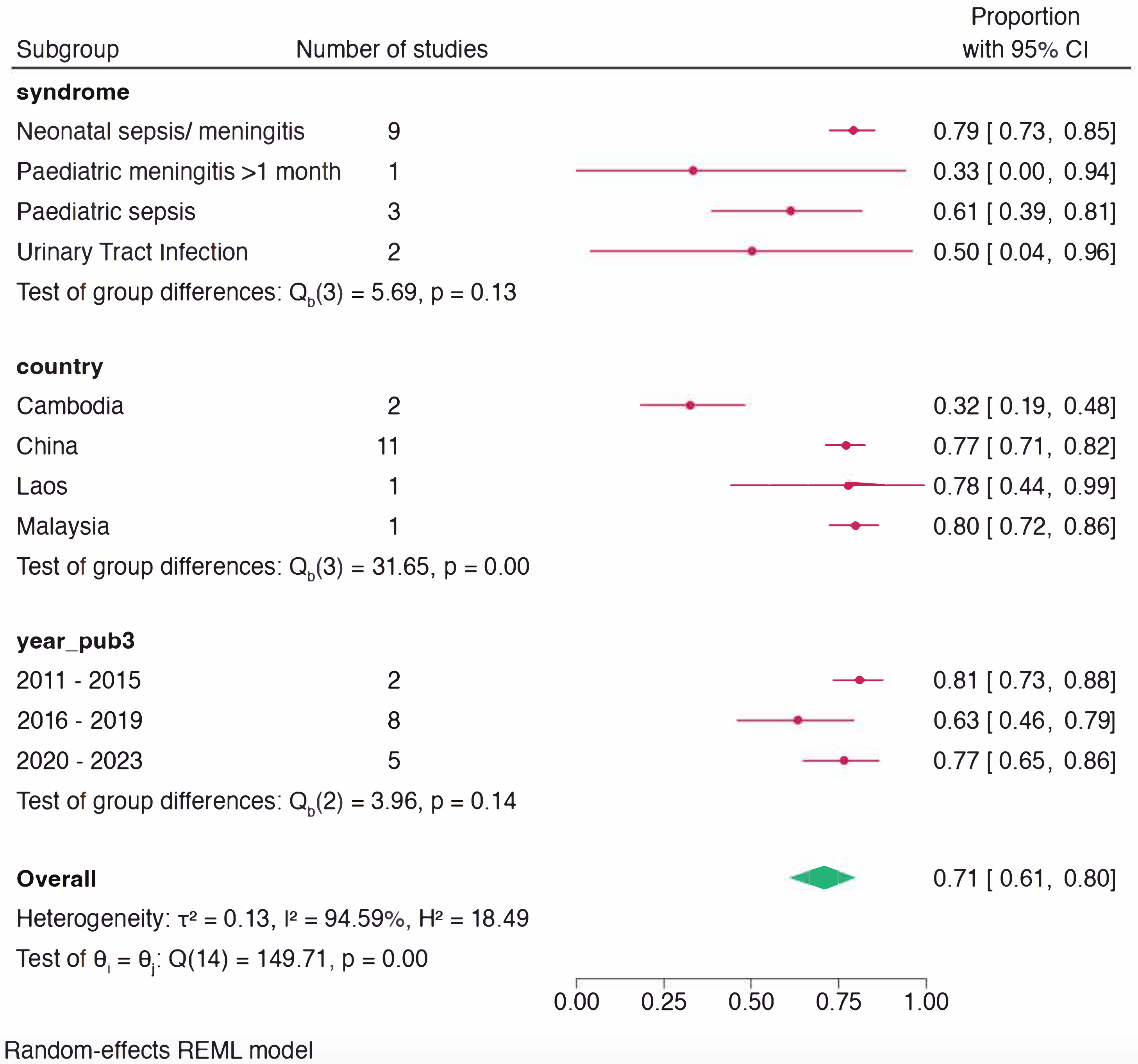

S1 Figure 12 Proportion of Klebsiella spp. isolates susceptibile to 3rd generation cephalosporins by subgroup

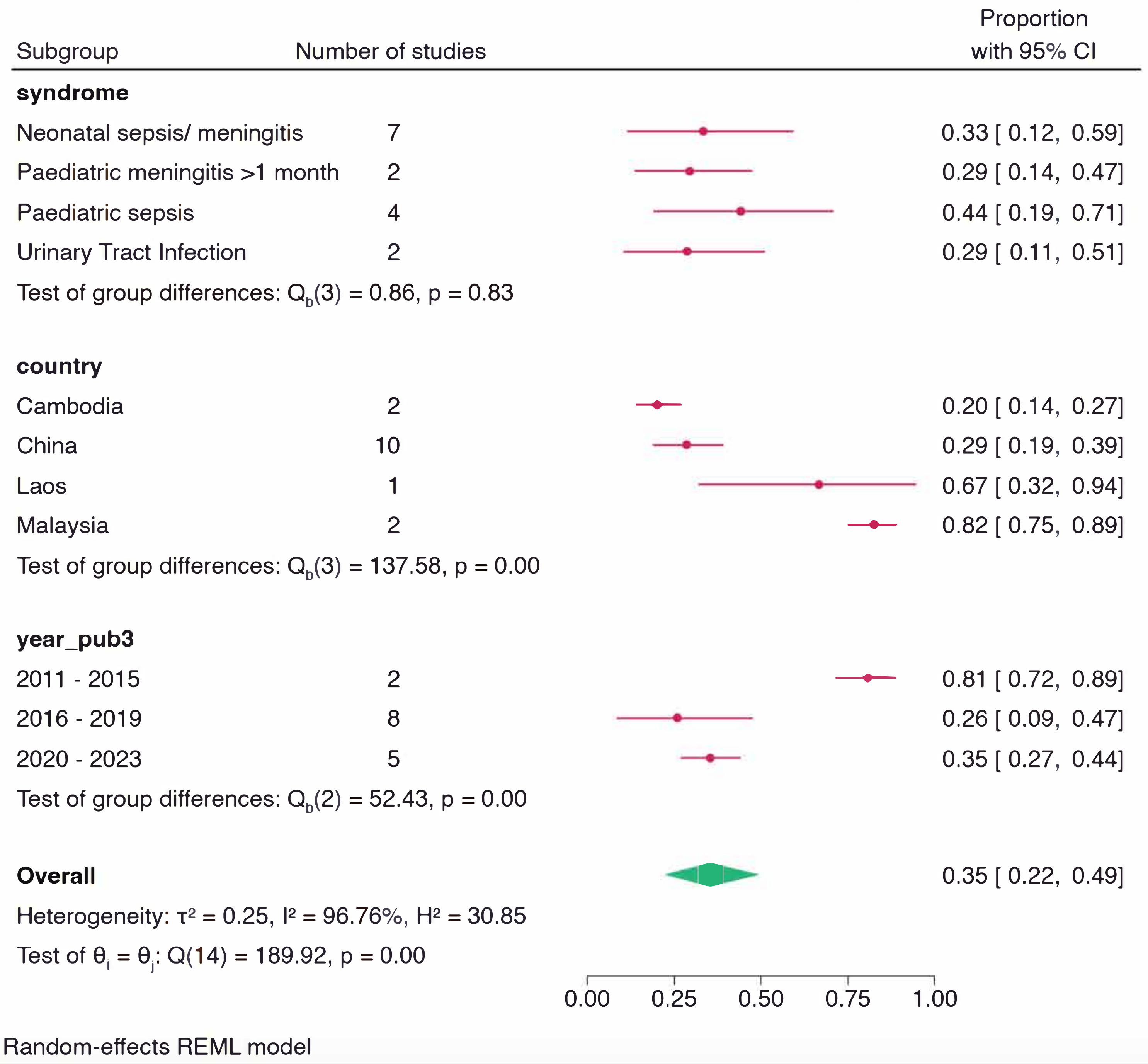

S1 Figure 13 Proportion of Klebsiella spp. isolates susceptibile to carbapenems by subgroup

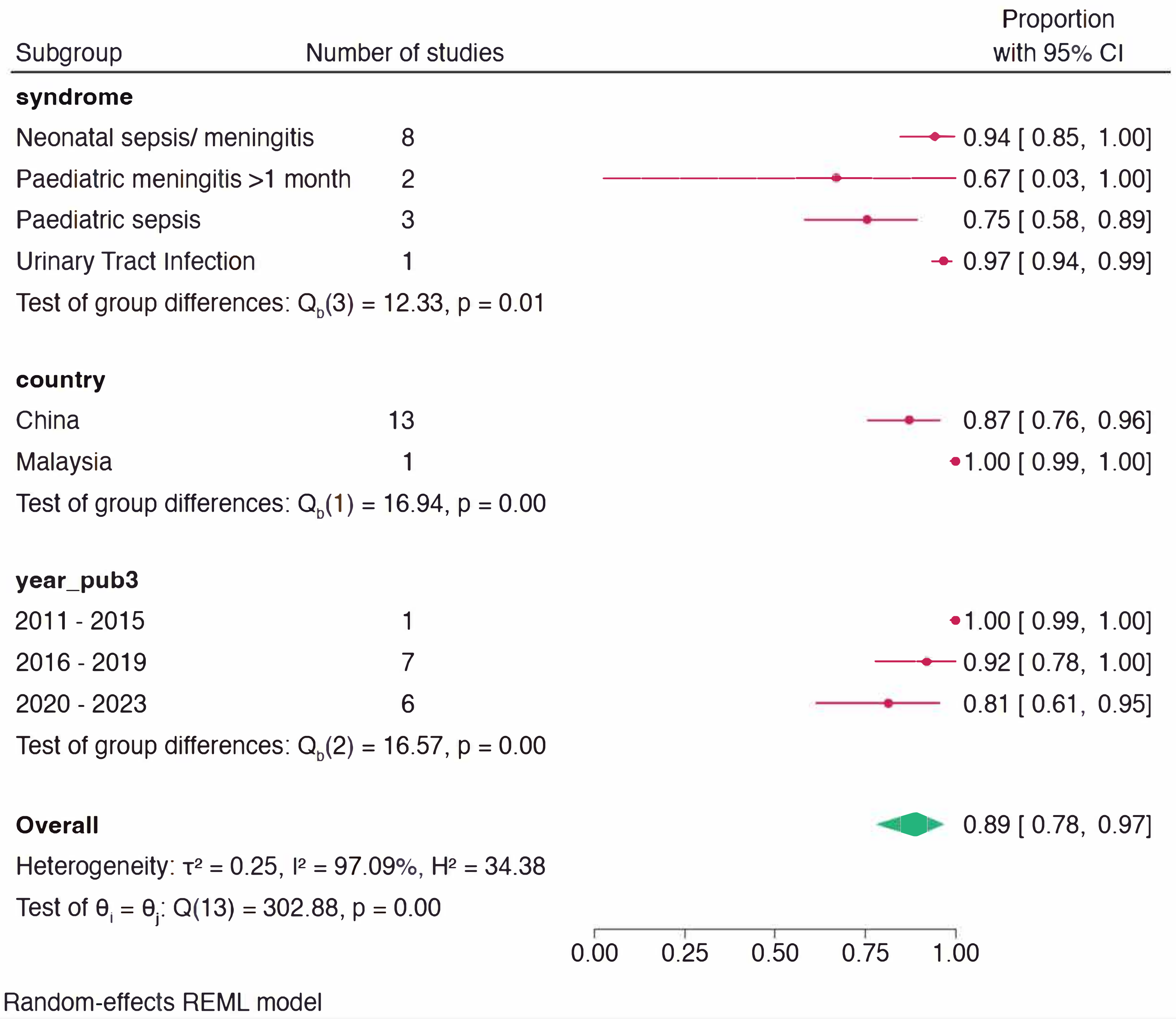

SI Figure I4

Org	Ab	subgroup	df	q	pvalue	tau	i2	h2	Overall_Tau2	Overall_I2
kleb	gent	Cambodia	1	2	0.157	0.029	50.12	2	0.126	94.59
kleb	gent	China	10	45.7	0	0.03	82.55	5.73	0.126	94.59
kleb	gent	Laos	0	0	.	0	.	.	0.126	94.59
kleb	gent	Malaysia	0	0	.	0	.	.	0.126	94.59
kleb	gent	Neonatal sep	8	25.74	0.001	0.033	72.15	3.59	0.126	94.59
kleb	gent	Paediatric m	0	0	.	0	.	.	0.126	94.59
kleb	gent	Paediatric s-	2	62.5	0	0.155	98.35	60.59	0.126	94.59
kleb	gent	Urinary Trac-	1	22.81	0	0.614	95.62	22.81	0.126	94.59
kleb	gent	2011	1	0.1	0.751	0	0	1	0.126	94.59
kleb	gent	2016	7	86.07	0	0.185	92.73	13.75	0.126	94.59
kleb	gent	2020	4	42.59	0	0.074	95.18	20.76	0.126	94.59
kleb	3gc	Cambodia	1	0.18	0.675	0	0	1	0.245	96.76
kleb	3gc	China	9	48.75	0	0.089	92.98	14.25	0.245	96.76
kleb	3gc	Laos	0	0	.	0	.	.	0.245	96.76
kleb	3gc	Malaysia	1	0.28	0.594	0	0	1	0.245	96.76
kleb	3gc	Neonatal sep	6	143.29	0	0.425	96.96	32.87	0.245	96.76
kleb	3gc	Paediatric m	1	0.04	0.847	0	0	1	0.245	96.76
kleb	3gc	Paediatric s-	3	33.87	0	0.266	98.38	61.89	0.245	96.76
kleb	3gc	Urinary Trac-	1	3.85	0.05	0.08	74.05	3.85	0.245	96.76
kleb	3gc	2011	1	1.06	0.302	0.004	6.03	1.06	0.245	96.76
kleb	3gc	2016	7	48.29	0	0.302	94.12	17.01	0.245	96.76
kleb	3gc	2020	4	21.11	0	0.032	88.36	8.59	0.245	96.76
kleb	carb	China	12	243.47	0	0.235	96.86	31.8	0.253	97.09
kleb	carb	Malaysia	0	0	.	0	.	.	0.253	97.09
kleb	carb	Neonatal sep	7	86.1	0	0.168	92.76	13.81	0.253	97.09
kleb	carb	Paediatric m	1	5.36	0.021	0.709	81.35	5.36	0.253	97.09
kleb	carb	Paediatric s-	2	93.52	0	0.101	97.18	35.52	0.253	97.09
kleb	carb	Urinary Trac-	0	0	.	0	.	.	0.253	97.09
kleb	carb	2011	0	0	.	0	.	.	0.253	97.09
kleb	carb	2016	6	108.88	0	0.208	94.14	17.06	0.253	97.09
kleb	carb	2020	5	122.92	0	0.286	98.02	50.43	0.253	97.09

S1 Figure 15 Proportion of Salmonella spp. isolates susceptibile to Ampicillin by subgroup

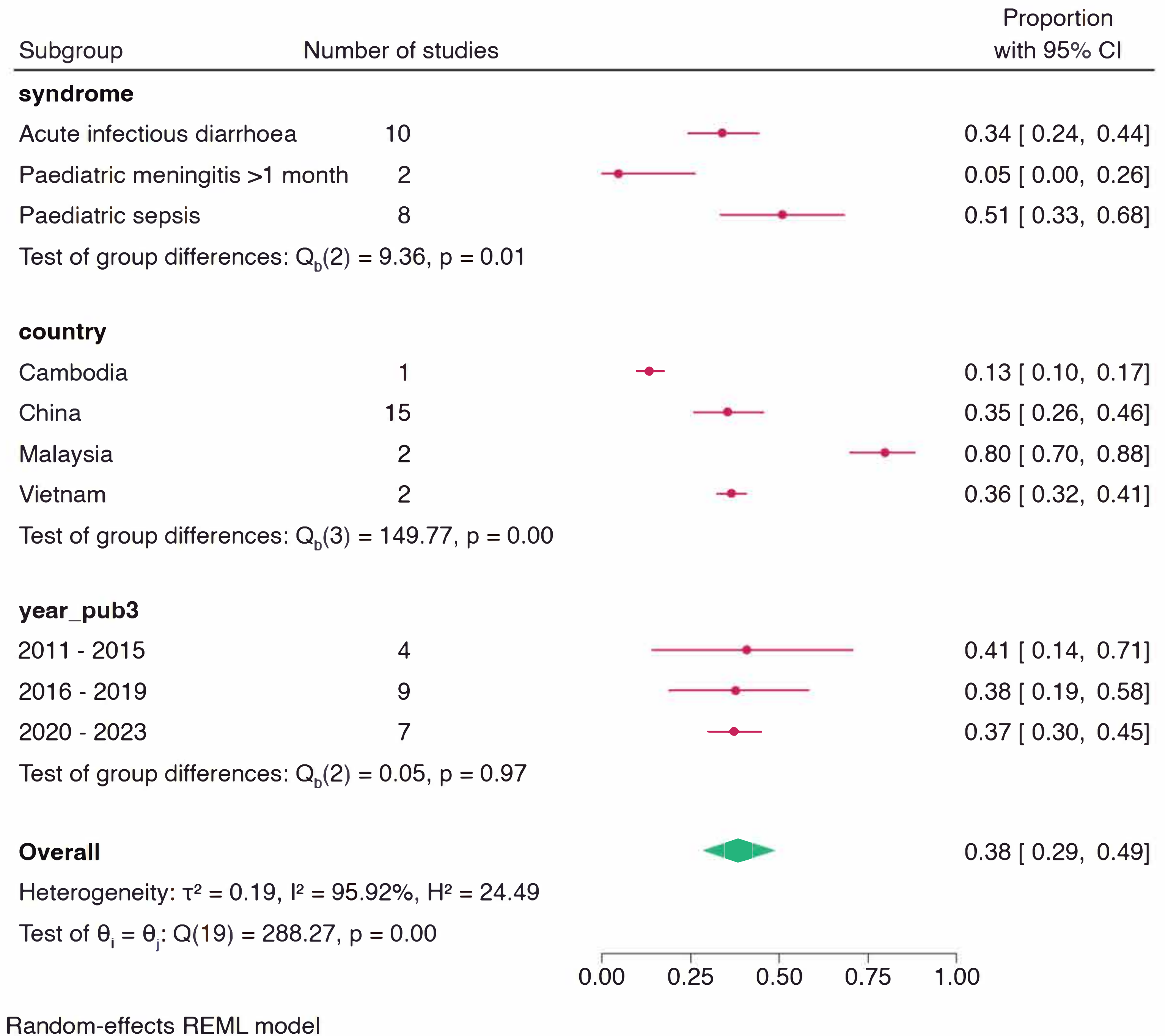

S1 Figure 16 Proportion of Salmonella spp. isolates susceptibile to Ampicillin by subgroup

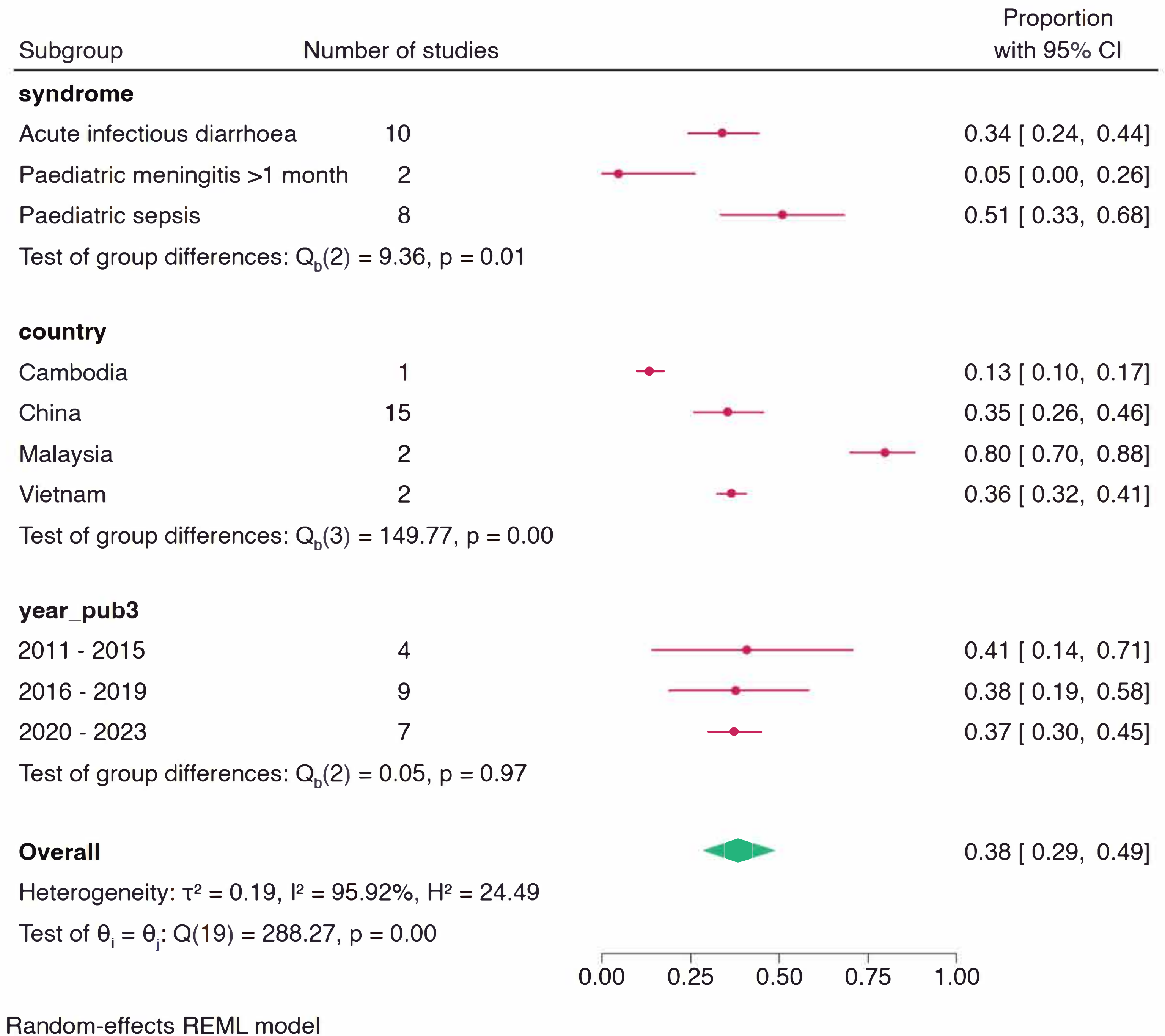

S1 Figure 17 Proportion of Salmonella spp. isolates susceptibile to Ciprofloxacin by subgroup

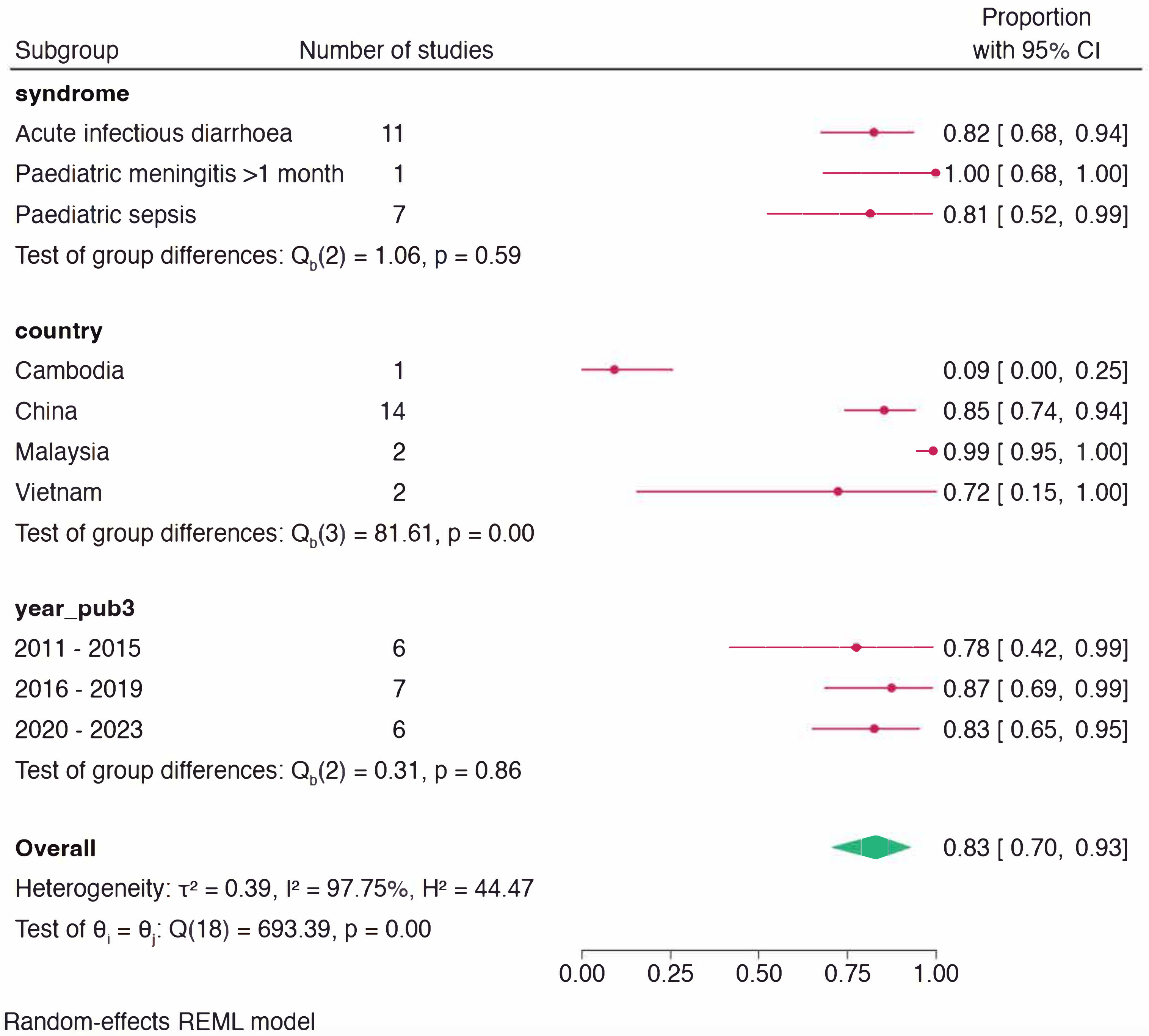

S1 Figure 18 Proportion of Salmonella spp. isolates susceptibile to Co-trimoxazole by subgroup

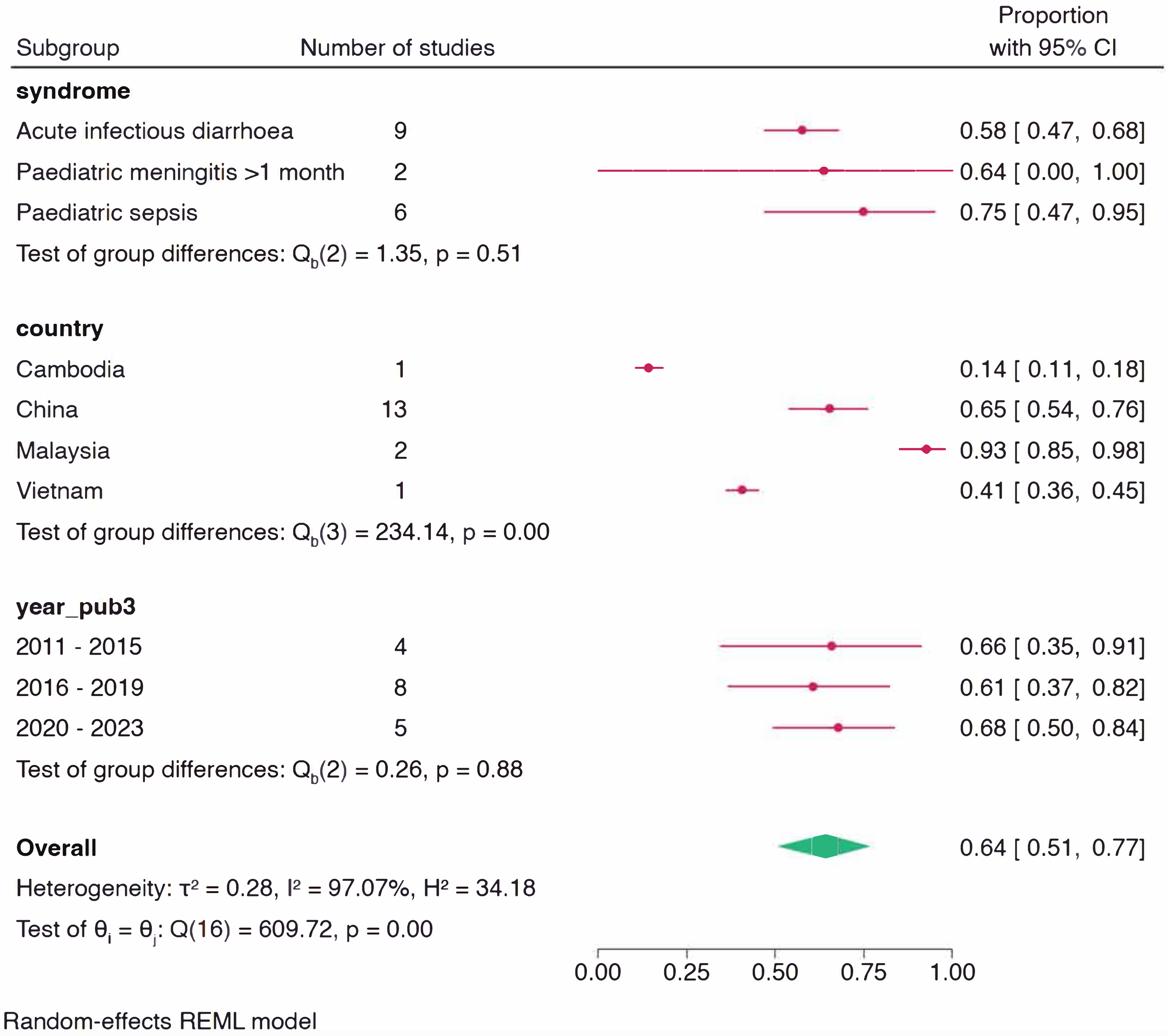

S1 Figure 19 Proportion of Salmonella spp. isolates susceptibile to Nalidixic acid by subgroup

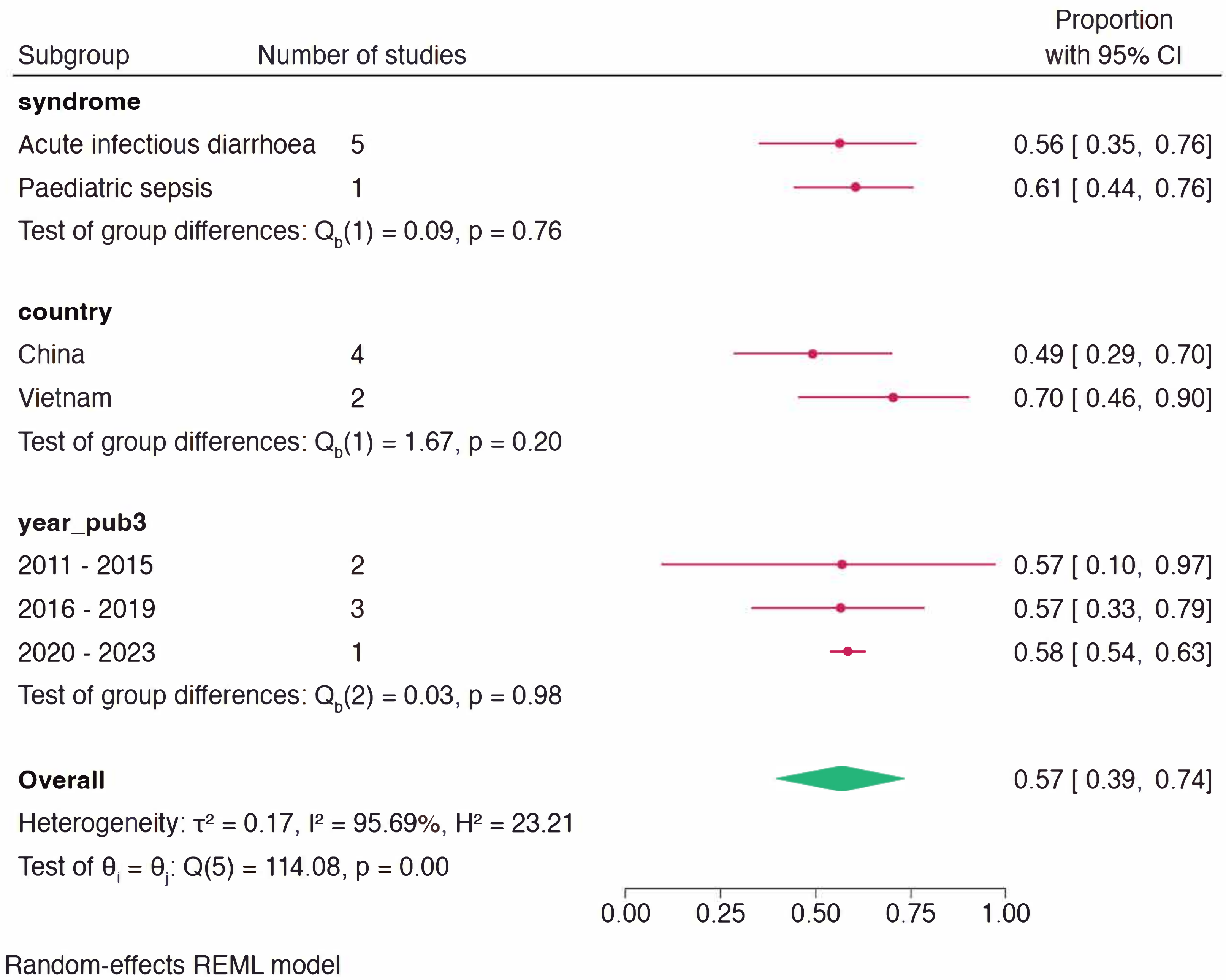

S1 Figure 20 Proportion of Salmonella spp. isolates susceptibile to 3rd Generation Cephalosporins by subgroup

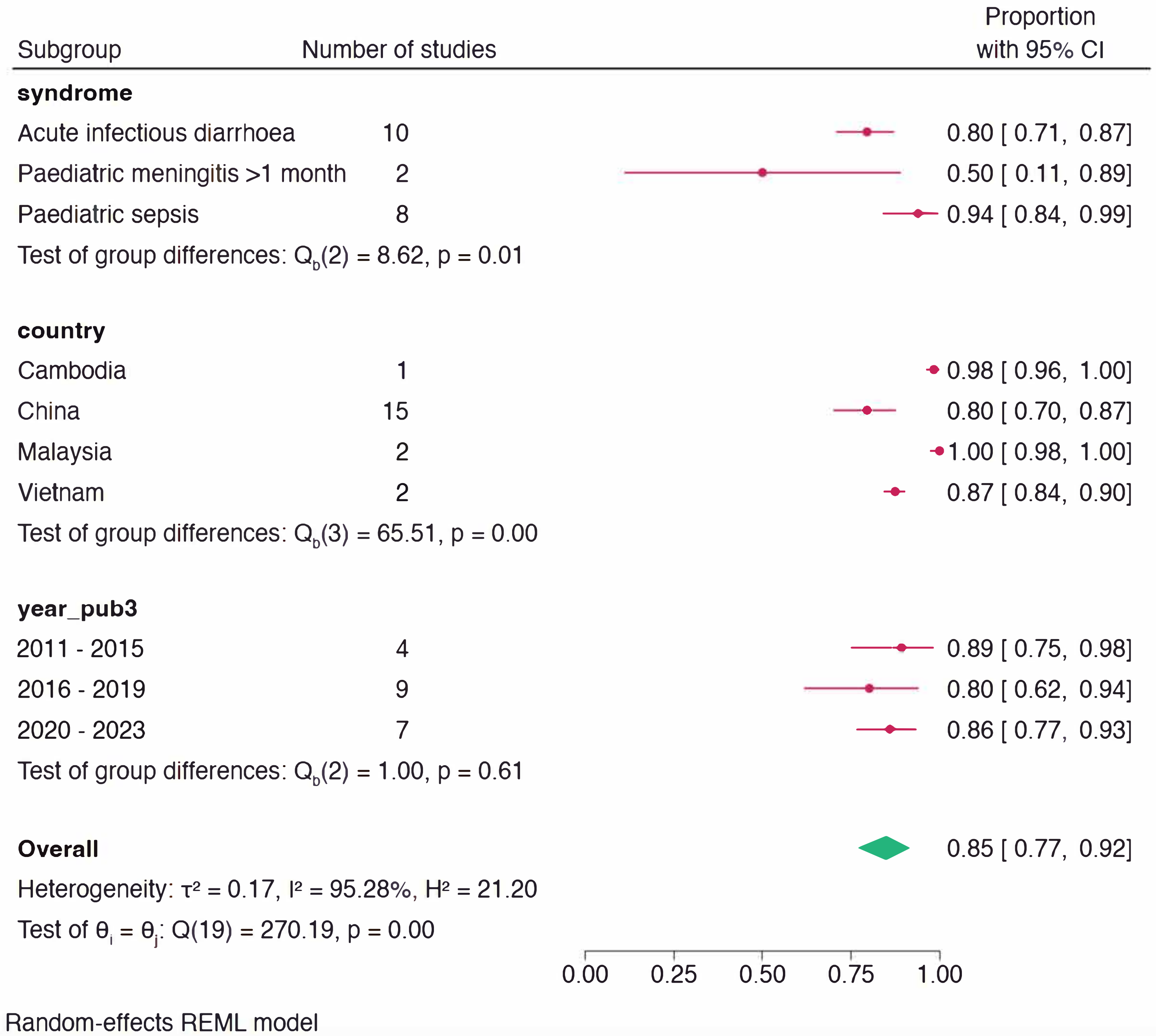

S1 Figure 21 Proportion of Salmonella spp. isolates susceptibile to Carbapenems by subgroup

Subgroup	Number of studies	Proportion with 95% CI
**syndrome**		
Acute infectious diarrhoea	5	0.99 [0.99, 1.00]
Paediatric meningitis >1 month	2	1.00 [0.86, 1.00]
Paediatric sepsis	2	0.96 [0.91, 0.99]
Test of group differences: $Q_b(2) = 11.62$, $p = 0.00$		
**country**		
China	8	1.00 [0.98, 1.00]
Vietnam	1	0.99 [0.98, 1.00]
Test of group differences: $Q_b(1) = 0.23$, $p = 0.63$		
**year_pub3**		
2011 - 2015	2	1.00 [0.98, 1.00]
2016 - 2019	2	1.00 [0.86, 1.00]
2020 - 2023	5	0.99 [0.96, 1.00]
Test of group differences: $Q_b(2) = 2.08$, $p = 0.35$		
**Overall**		1.00 [0.99, 1.00]
Heterogeneity: $\tau^2 = 0.01$, $I^2 = 51.22\%$, $H^2 = 2.05$		
Test of $\theta_i = \theta_j$: $Q(8) = 13.71$, $p = 0.09$		

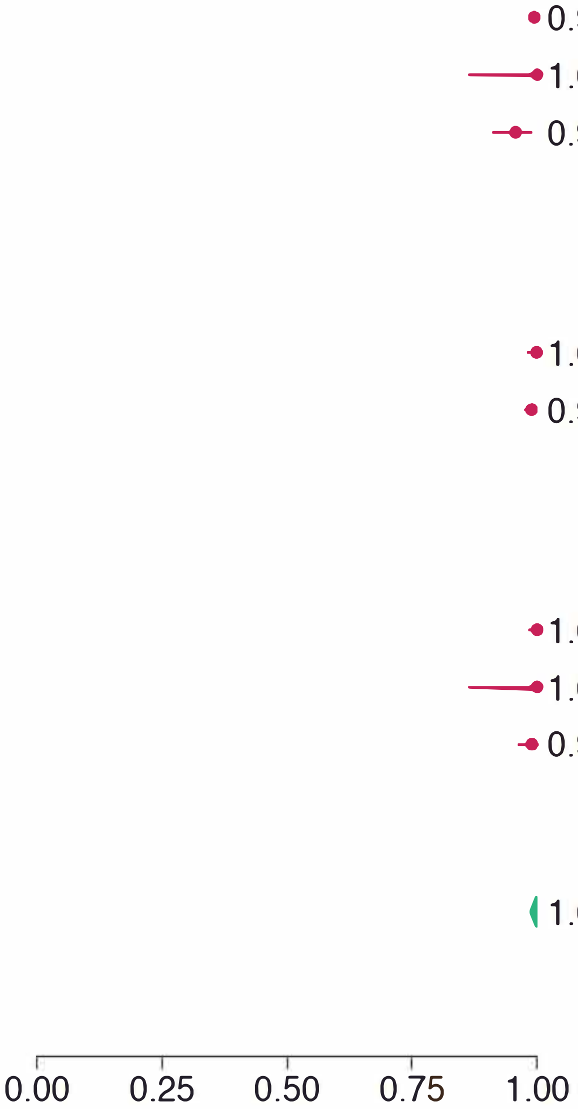

Random-effects REML model

SI Ffgure 22

Org	Ab	subgroup	df	q	pvalue	tau	i2	h2	Overall_Tau2	Overall_I2
salmonella	Ampicillin	China	14	130.37	0	0.13	93.18	14.67	0.19	95.92
salmonella	Ampicillin	Malaysia	1	0.02	0.884	0	0	1	0.19	95.92
salmonella	Ampicillin	Vietnam	1	0.16	0.691	0	0	1	0.19	95.92
salmonella	Ampicillin	Acute infect-	9	89.04	0	0.103	93.62	15.67	0.19	95.92
salmonella	Ampicillin	Paediatric m	1	1.03	0.311	0.004	2.73	1.03	0.19	95.92
salmonella	Ampicillin	Paediatric s-	7	190	0	0.229	96.39	27.67	0.19	95.92
salmonella	Ampicillin	2011	3	60.6	0	0.361	97.08	34.24	0.19	95.92
salmonella	Ampicillin	2016	8	169.39	0	0.332	96.33	27.25	0.19	95.92
salmonella	Ampicillin	2020	6	31.97	0	0.032	83.88	6.2	0.19	95.92
salmonella	naldixic	China	3	33.68	0	0.162	91.62	11.93	0.173	95.69
salmonella	naldixic	Vietnam	1	17.81	0	0.126	94.38	17.81	0.173	95.69
salmonella	naldixic	Acute infect-	4	112.6	0	0.215	96.96	32.85	0.173	95.69
salmonella	naldixic	Paediatric s-	0	0	.	0	.	.	0.173	95.69
salmonella	naldixic	2011	1	73.91	0	0.582	98.65	73.91	0.173	95.69
salmonella	naldixic	2016	2	14.18	0.001	0.142	85.29	6.8	0.173	95.69
salmonella	naldixic	2020	0	0	.	0	.	.	0.173	95.69
salmonella	cipro	Cambodia	0	0	.	0	.	.	0.392	97.75
salmonella	cipro	China	13	235.11	0	0.239	96.29	26.94	0.392	97.75
salmonella	cipro	Malaysia	1	1.23	0.268	0.006	18.38	1.23	0.392	97.75
salmonella	cipro	Vietnam	1	100.39	0	0.76	99	100.39	0.392	97.75
salmonella	cipro	Acute infect-	10	564.89	0	0.317	97.78	45.03	0.392	97.75
salmonella	cipro	Paediatric m	0	0	.	0	.	.	0.392	97.75
salmonella	cipro	Paediatric s-	6	120.18	0	0.645	97.75	44.35	0.392	97.75
salmonella	cipro	2011	5	187.36	0	0.823	98.57	69.96	0.392	97.75
salmonella	cipro	2016	6	113.27	0	0.309	95.01	20.03	0.392	97.75
salmonella	cipro	2020	5	244.23	0	0.219	97.27	36.64	0.392	97.75
salmonella	cotri	Cambodia	0	0	.	0	.	.	0.276	97.07
salmonella	cotri	China	12	123.59	0	0.14	92.96	14.21	0.276	97.07
salmonella	cotri	Malaysia	1	1.07	0.3	0.002	6.74	1.07	0.276	97.07
salmonella	cotri	Vietnam	0	0	.	0	.	.	0.276	97.07
salmonella	cotri	Acute infect-	8	121.4	0	0.09	92.95	14.18	0.276	97.07
salmonella	cotri	Paediatric m	1	12.16	0	1.603	91.78	12.16	0.276	97.07
salmonella	cotri	Paediatric s-	5	475.26	0	0.466	97.79	45.17	0.276	97.07
salmonella	cotri	2011	3	68.04	0	0.396	97.5	39.97	0.276	97.07
salmonella	cotri	2016	7	282.11	0	0.394	96.55	28.98	0.276	97.07
salmonella	cotri	2020	4	181.35	0	0.153	96.04	25.22	0.276	97.07
salmonella	3GC	Cambodia	0	0	.	0	.	.	0.172	95.28
salmonella	3GC	China	14	158.12	0	0.134	93.11	14.51	0.172	95.28
salmonella	3GC	Malaysia	1	0	0.951	0	0	1	0.172	95.28
salmonella	3GC	Vietnam	1	0.02	0.896	0	0.01	1	0.172	95.28
salmonella	3GC	Acute infect-	9	115.05	0	0.087	92.51	13.36	0.172	95.28
salmonella	3GC	Paediatric m	1	2.26	0.133	0.168	55.75	2.26	0.172	95.28
salmonella	3GC	Paediatric s-	7	80.1	0	0.164	94.34	17.67	0.172	95.28
salmonella	3GC	2011	3	23.71	0	0.13	92.26	12.92	0.172	95.28
salmonella	3GC	2016	8	178.4	0	0.311	95.87	24.2	0.172	95.28
salmonella	3GC	2020	6	64.75	0	0.086	92.93	14.15	0.172	95.28

S1 Figure 22 cont

Org	Ab	subgroup	df	q	pvalue	tau	i2	h2	Overall_Tau2	Overall_I2
salmonella	carb	China	7	12.65	0.081	0.017	49.23	1.97	0.012	51.22
salmonella	carb	Vietnam	0	0	.	0	.	.	0.012	51.22
salmonella	carb	Acute infect-	4	1.53	0.82	0	0.01	1	0.012	51.22
salmonella	carb	Paediatric m	1	0	1	0	0	1	0.012	51.22
salmonella	carb	Paediatric s-	1	0.55	0.456	0	0	1	0.012	51.22
salmonella	carb	2011	1	0	0.977	0	0	1	0.012	51.22
salmonella	carb	2016	1	0	1	0	0	1	0.012	51.22
salmonella	carb	2020	4	11.37	0.023	0.017	68.02	3.13	0.012	51.22
salmonella	azithromycin	China	0	0	.	0	.	.	0	0
salmonella	azithromycin	Vietnam	0	0	.	0	.	.	0	0
salmonella	azithromycin	Acute	1	0.04	0.835	0	0	1	0	0
salmonella	azithromycin	2016	0	0	.	0	.	.	0	0
salmonella	azithromycin	2020	0	0	.	0	.	.	0	0

S1 Figure 23 Proportion of Acinetobacter spp. isolates susceptibile to Gentamicin by subgroup

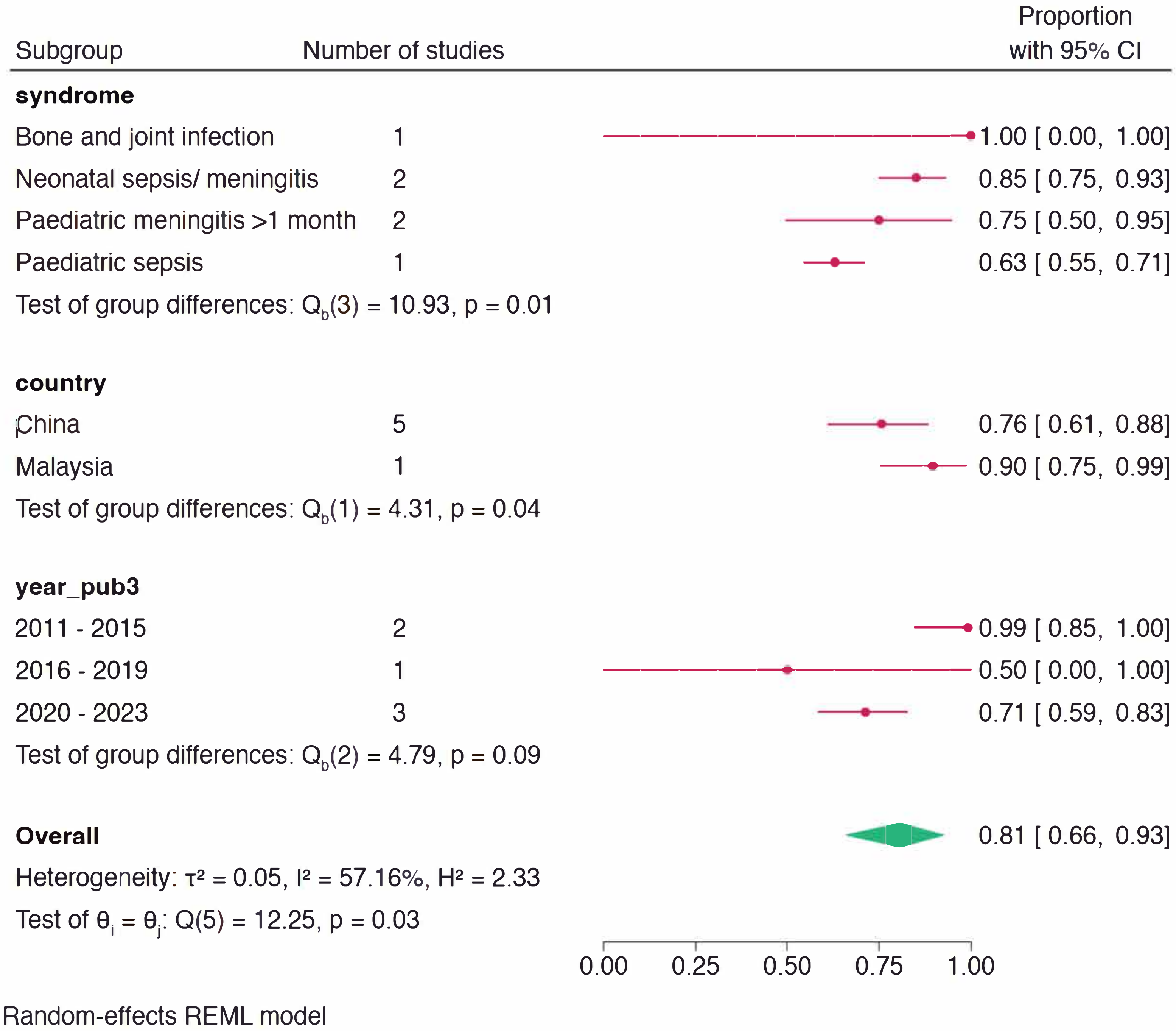

S1 Figure 24 Proportion of Acinetobacter spp. isolates susceptibile to Carbapenems by subgroup

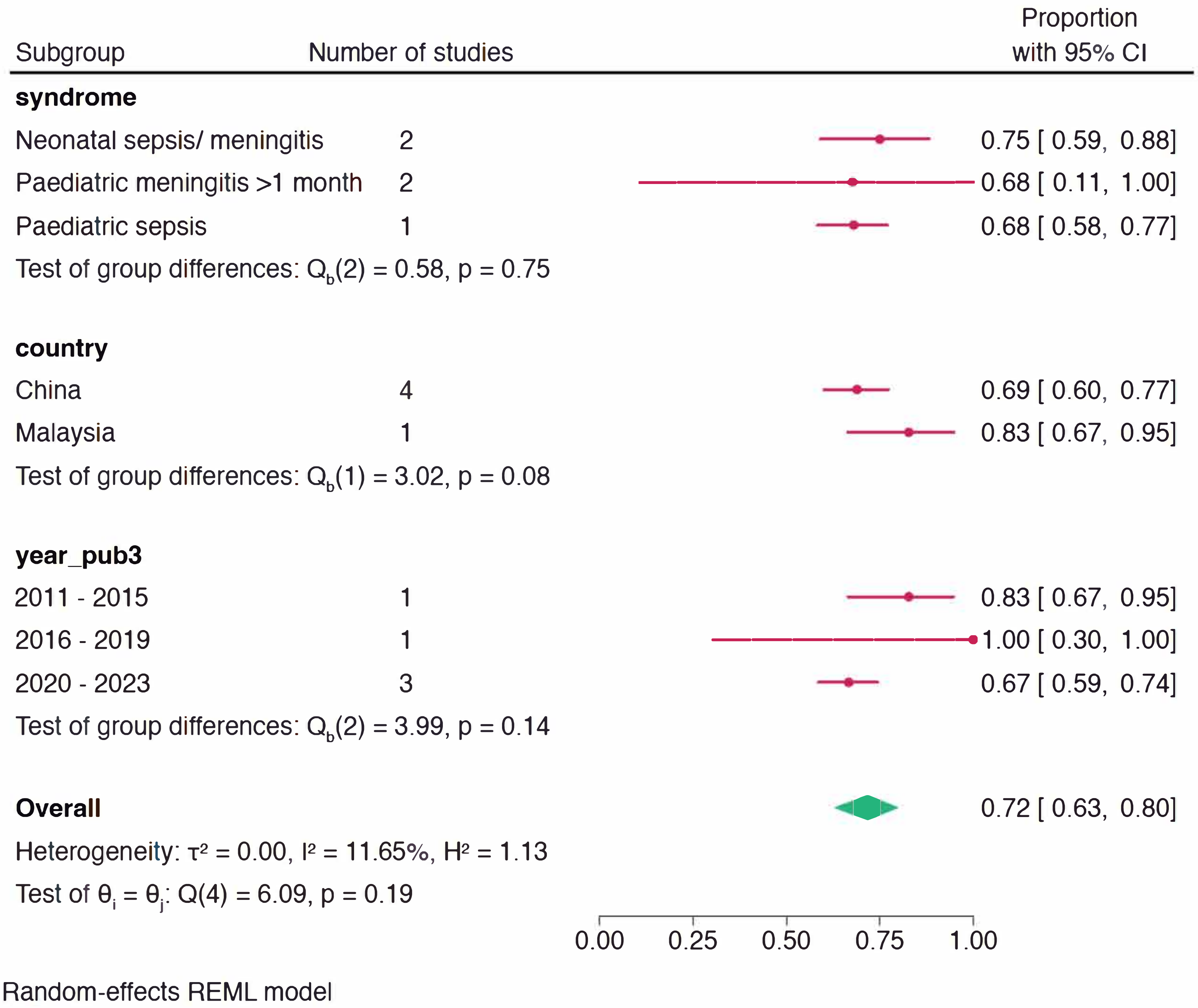

S1 Figure 25

Org	Ab	subgroup	df	q	pvalue	tau	i2	h2	Overall_Tau2	Overall_I2
acinetobacte	gent	China	4	5.33	0.255	0.026	37.48	1.6	0.05	57.16
acinetobacte	gent	Malaysia	0	0	.	0	.	.	0.05	57.16
acinetobacte	gent	Bone and joi	0	0	.	0	.	.	0.05	57.16
acinetobacte	gent	Neonatal sep	1	0.83	0.361	0	0	1	0.05	57.16
acinetobacte	gent	Paediatric m	1	0.48	0.486	0	0	1	0.05	57.16
acinetobacte	gent	Paediatric s-	0	0	.	0	.	.	0.05	57.16
acinetobacte	gent	2011	1	0.01	0.915	0	0	1	0.05	57.16
acinetobacte	gent	2016	0	0	.	0	.	.	0.05	57.16
acinetobacte	gent	2020	2	4.74	0.094	0.03	56.62	2.31	0.05	57.16
acinetobacte	carb	China	3	3.07	0.38	0	0	1	0.005	11.65
acinetobacte	carb	Malaysia	0	0	.	0	.	.	0.005	11.65
acinetobacte	carb	Neonatal sep	1	1.88	0.17	0.027	46.77	1.88	0.005	11.65
acinetobacte	carb	Paediatric m	1	2.22	0.136	0.296	54.89	2.22	0.005	11.65
acinetobacte	carb	Paediatric s-	0	0	.	0	.	.	0.005	11.65
acinetobacte	carb	2011	0	0	.	0	.	.	0.005	11.65
acinetobacte	carb	2016	0	0	.	0	.	.	0.005	11.65
acinetobacte	carb	2020	2	2.1	0.35	0	0	1	0.005	11.65

S1 Figure 26 Proportion of P. aeruginosa isolates susceptibile to Gentamicin by subgroup

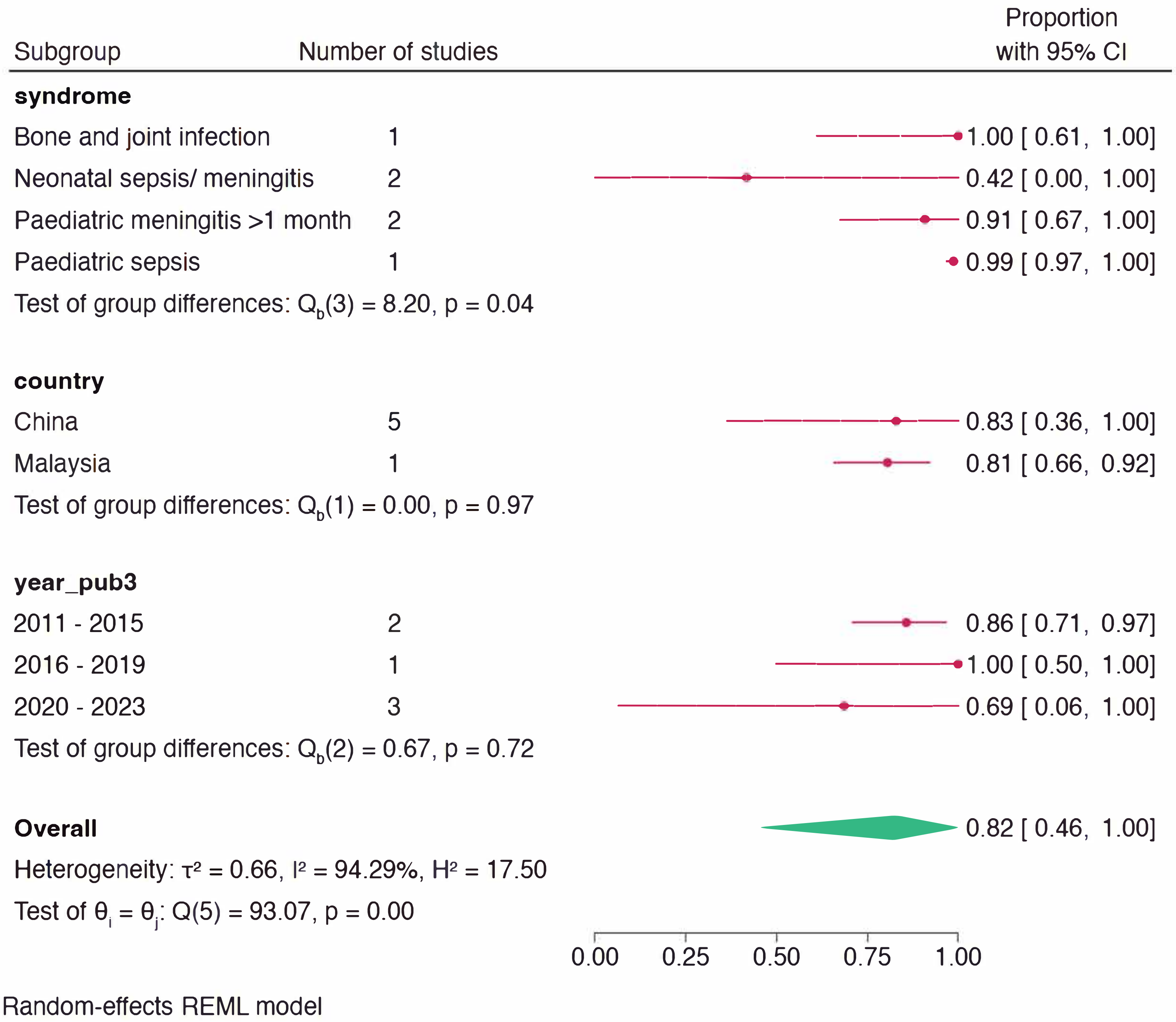

S1 Figure 27 Proportion of P. aeruginosa isolates susceptibile to Gentamicin by subgroup

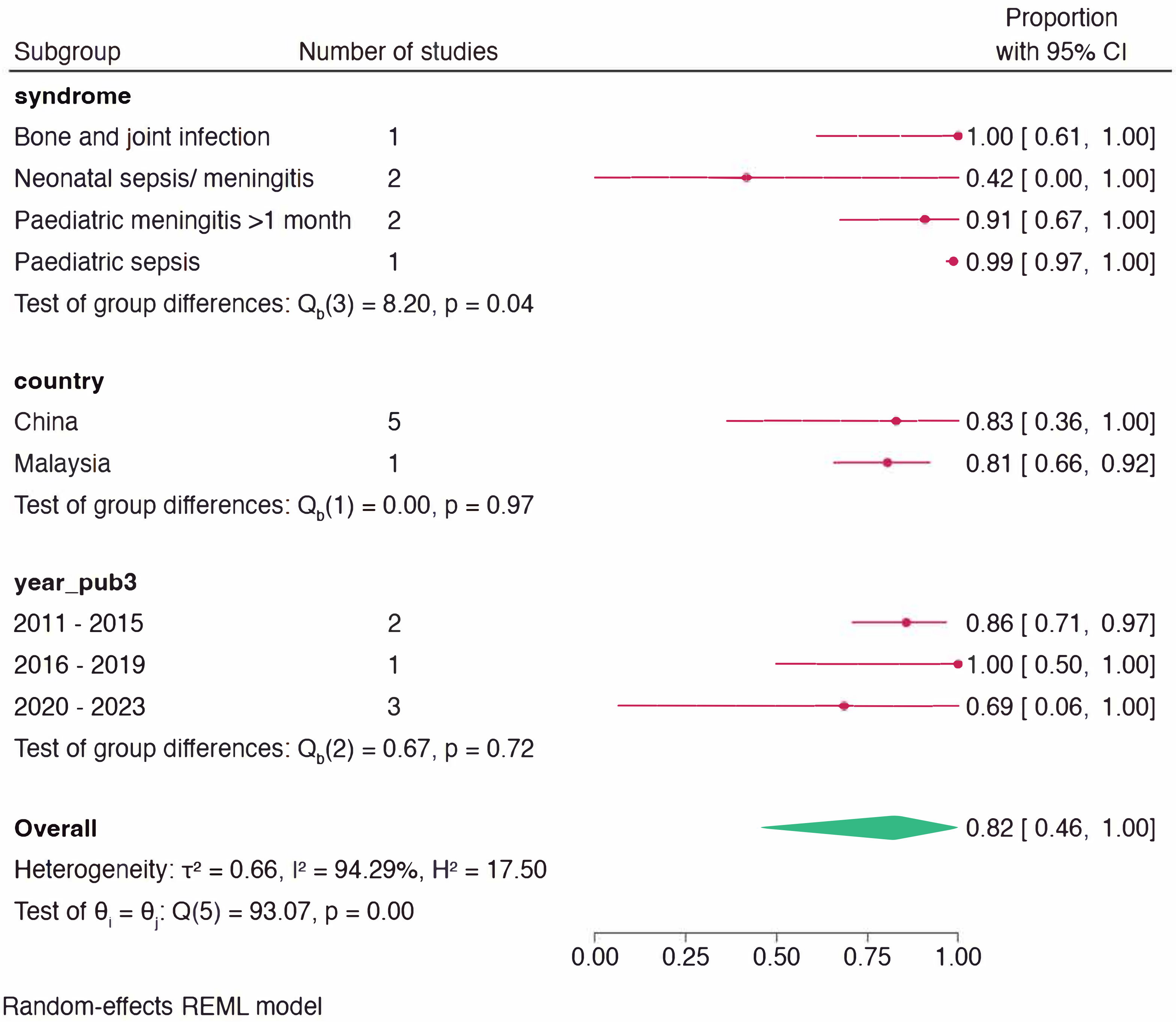

S1 Figure 28

**Org**	**Ab**	subgroup	df	q	pvalue	tau	i2	h2	Overall_Tau2	Overall_I2
pseudomona	gent	China	4	84.64	0	0.848	93.59	15.61	0.659	94.29
pseudomona	gent	Malaysia	0	0	.	0	.	.	0.659	94.29
pseudomona	gent	Bone and joi	0	0	.	0	.	.	0.659	94.29
pseudomona	gent	Neonatal sep	1	29.56	0	1.257	96.62	29.56	0.659	94.29
pseudomona	gent	Paediatric m	1	0.33	0.565	0	0	1	0.659	94.29
pseudomona	gent	Paediatric s-	0	0	.	0	.	.	0.659	94.29
pseudomona	gent	2011	1	0.88	0.347	0	0	1	0.659	94.29
pseudomona	gent	2016	0	0	.	0	.	.	0.659	94.29
pseudomona	gent	2020	2	84.59	0	1.38	97.43	38.87	0.659	94.29
pseudomona	carb	China	3	9.05	0.029	0.105	65.51	2.9	0.066	61.66
pseudomona	carb	Malaysia	0	0	.	0	.	.	0.066	61.66
pseudomona	carb	Neonatal sep	1	6.24	0.012	0.231	83.98	6.24	0.066	61.66
pseudomona	carb	Paediatric m	1	0.33	0.565	0	0	1	0.066	61.66
pseudomona	carb	Paediatric s-	0	0	.	0	.	.	0.066	61.66
pseudomona	carb	2011	0	0	.	0	.	.	0.066	61.66
pseudomona	carb	2016	0	0	.	0	.	.	0.066	61.66
pseudomona	carb	2020	2	8.8	0.012	0.13	76.05	4.17	0.066	61.66
[illegible]	[illegible]	[illegible]	[illegible]	[illegible]	[illegible]	[illegible]	[illegible]	[illegible]	[illegible]	[illegible]

S1 Figure 29 Proportion of H. influenzae isolates susceptibile to Ampicillin by subgroup

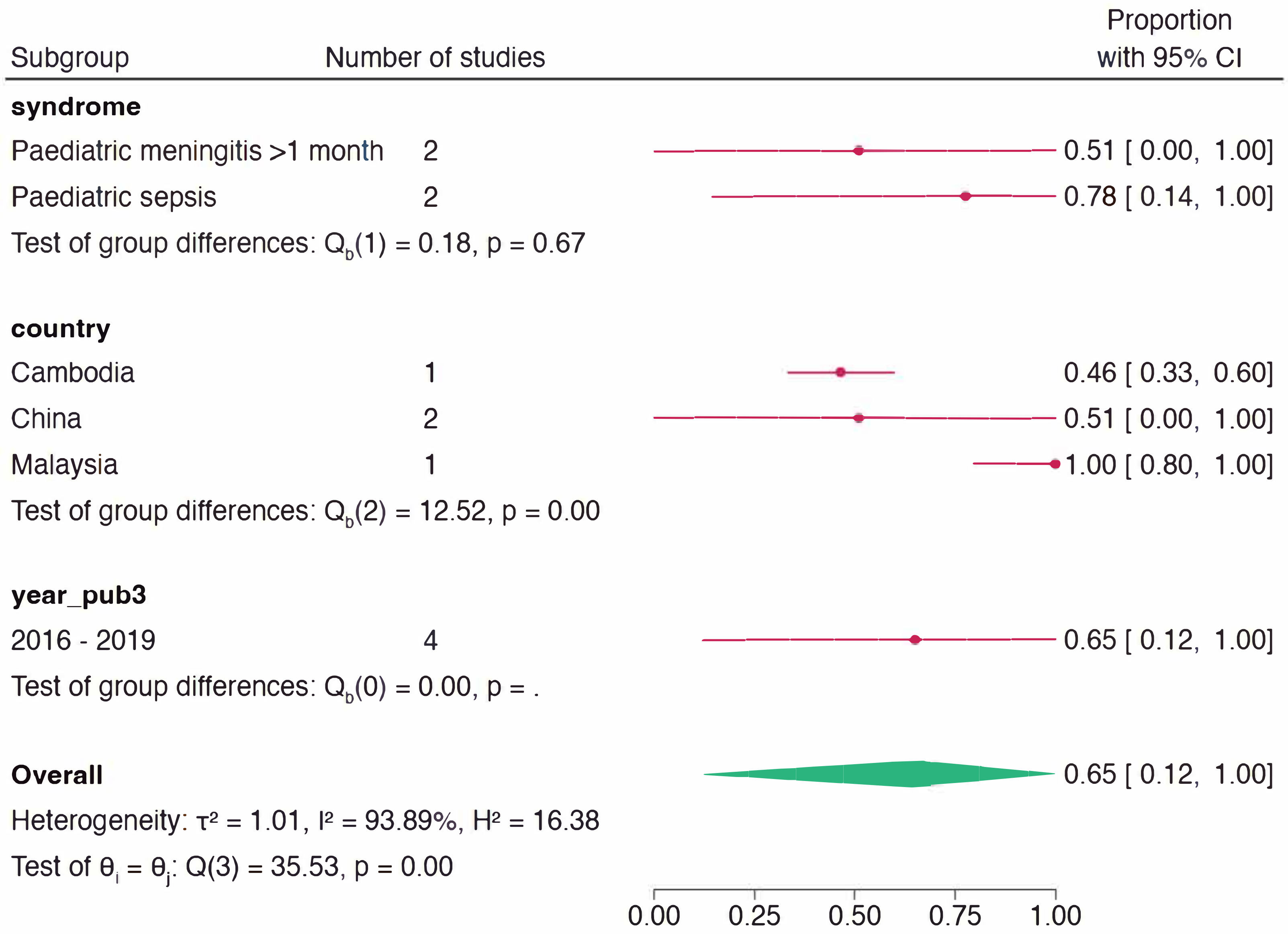

Random-effects REML model

S1 Figure 30 Proportion of H. influenzae isolates susceptibile to 3rd Generation Cephalosporins by subgroup

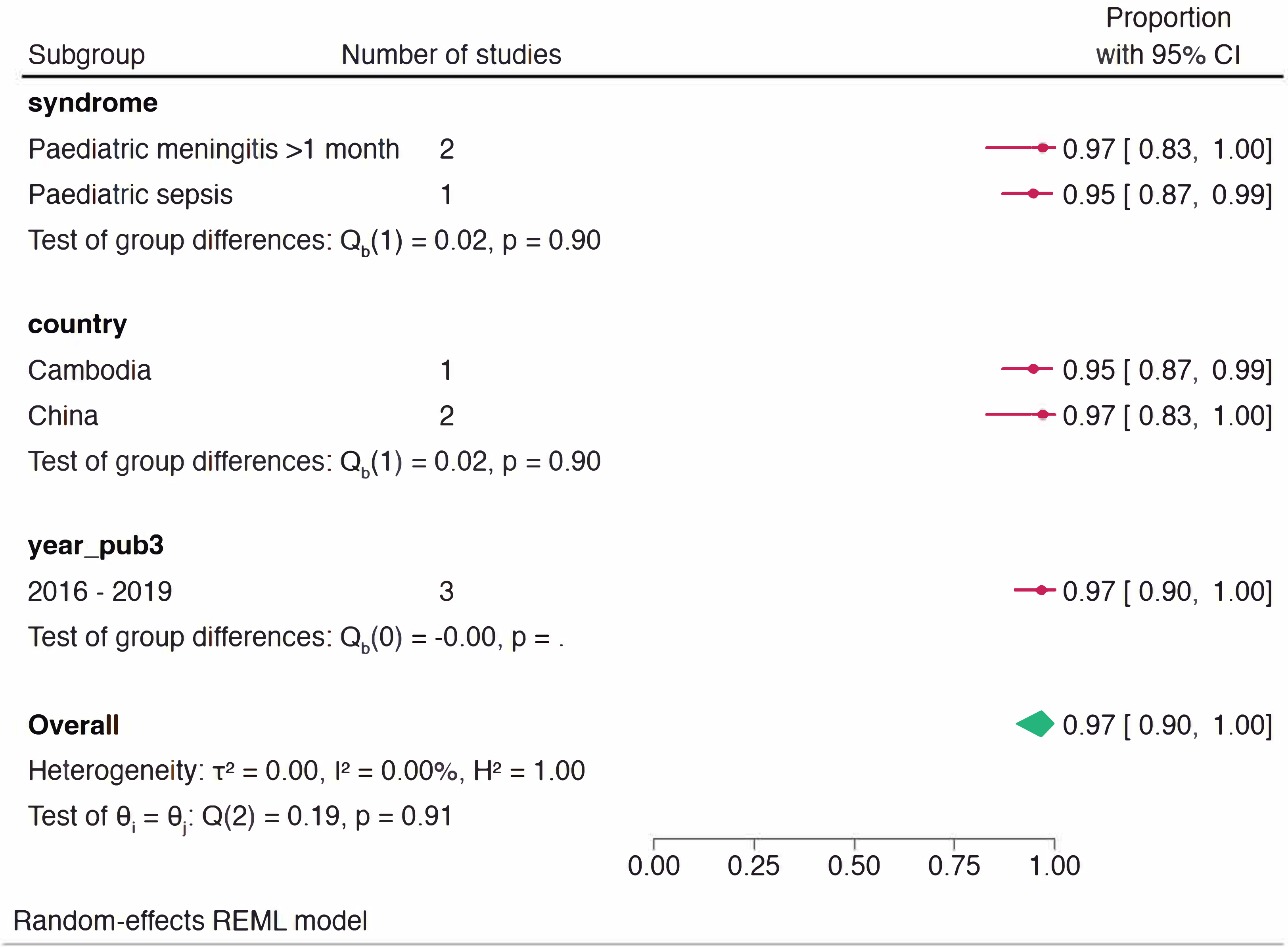

S1 Figure 31

**Org**	**Ab**	subgroup	df	q	pvalue	tau	i2	h2	Overall_Tau2	Overall_I2
H. influenzae	Ampicillin	Cambodia	0	0	.	0	.	.	1.009	93.89
H. influenzae	Ampicillin	China	1	15.79	0	2.066	93.67	15.79	1.009	93.89
H. influenzae	Ampicillin	Malaysia	0	0	.	0	.	.	1.009	93.89
H. influenzae	Ampicillin	meningitis	1	15.79	0	2.066	93.67	15.79	1.009	93.89
H. influenzae	Ampicillin	sepsis	1	12.51	0	0.779	92.01	12.51	1.009	93.89
H. influenzae	Ampicillin	2016	3	35.53	0	1.009	93.89	16.38	1.009	93.89
H. influenzae	3gc	Cambodia	0	0	.	0	.	.	0	0
H. influenzae	3gc	China	1	0.17	0.679	0	0	1	0	0
H. influenzae	3gc	meningitis	1	0.17	0.679	0	0	1	0	0
H. influenzae	3gc	sepsis	0	0	.	0	.	.	0	0
H. influenzae	3gc	2016	2	0.19	0.91	0	0	1	0	0
[illegible]	[illegible]	[illegible]	[illegible]	[illegible]	[illegible]	[illegible]	[illegible]	[illegible]	[illegible]	[illegible]

S1 Figure 32 Proportion of S. pneumoniae isolates susceptibile to ampicillin by subgroup

Subgroup	Number of studies	Proportion with 95% CI
**syndrome**		
Paediatric meningitis >1 month	11	0.26 [0.11, 0.44]
Paediatric sepsis	12	0.83 [0.71, 0.93]
Test of group differences: $Q_b(1) = 24.76$, $p = 0.00$		
**country**		
Cambodia	1	0.49 [0.41, 0.57]
China	19	0.54 [0.34, 0.73]
Malaysia	3	0.90 [0.71, 1.00]
Test of group differences: $Q_b(2) = 13.00$, $p = 0.00$		
**year_pub3**		
2011 - 2015	2	0.48 [0.00, 1.00]
2016 - 2019	16	0.59 [0.39, 0.77]
2020 - 2023	5	0.61 [0.27, 0.90]
Test of group differences: $Q_b(2) = 0.04$, $p = 0.98$		
**Overall**		0.59 [0.41, 0.75]
Heterogeneity: $\tau^2 = 0.63$, $I^2 = 98.46\%$, $H^2 = 64.88$		
Test of $\theta_i = \theta_j$: $Q(22) = 1052.53$, $p = 0.00$		

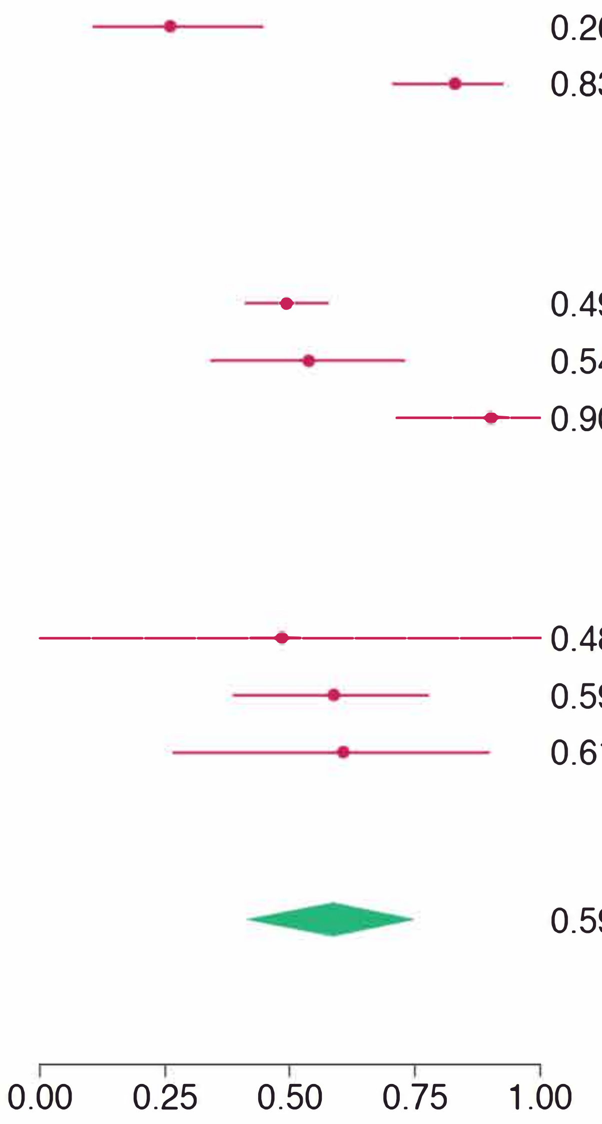

Random-effects REML model

S1 Figure 33 Proportion of S. pneumoniae isolates susceptibile to chloramphenicol by subgroup

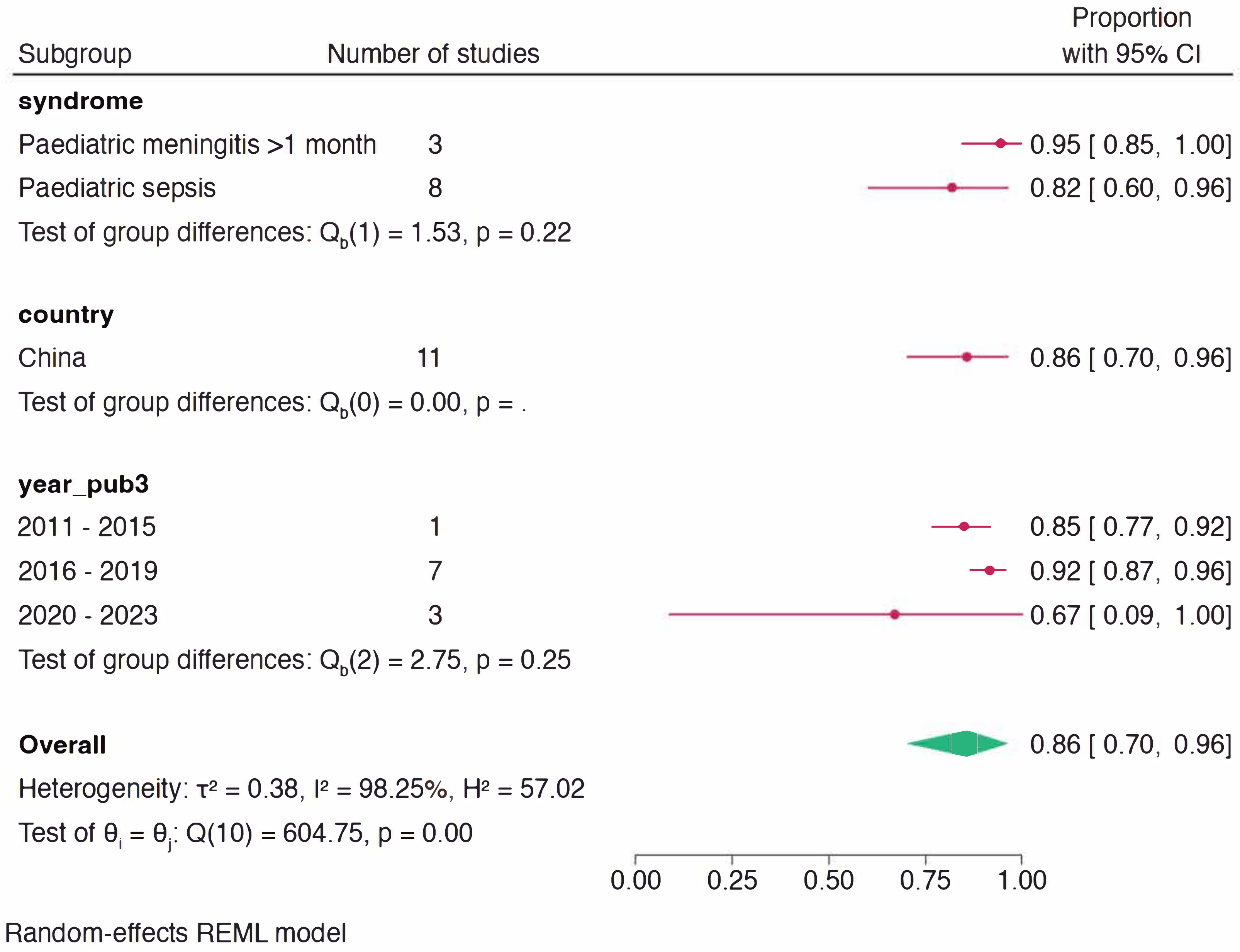

S1 Figure 34 Proportion of S. pneumoniae isolates susceptibile to 3rd generation cephalosporins by subgroup

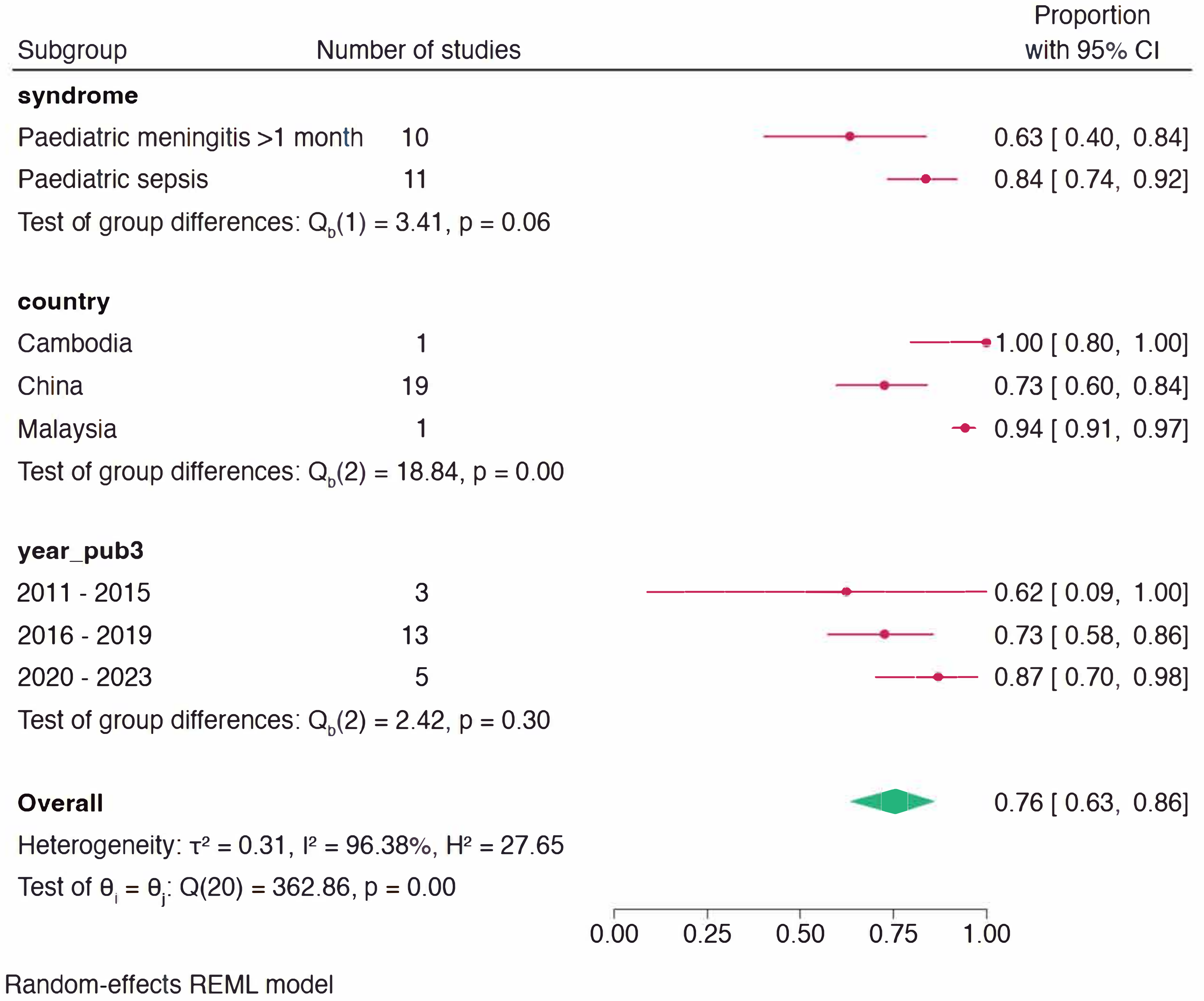

S1 Figure 35 Proportion of S. pneumoniae isolates susceptibile to carbapenems by subgroup

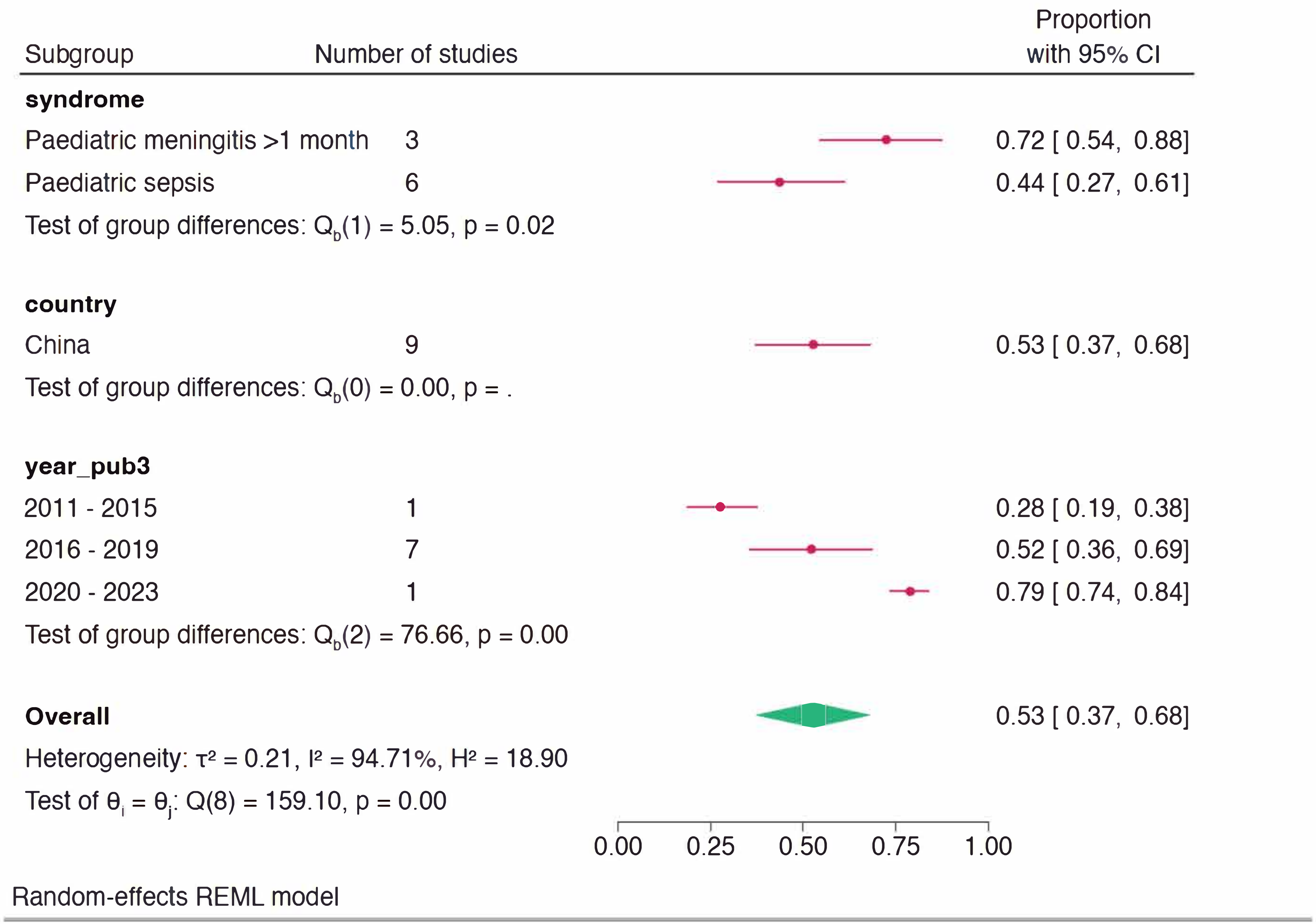

S1 Figure 36

**Org**	**Ab**	subgroup	df	q	pvalue	tau	i2	h2	Overall_Tau2	Overall_I2
s. pneumoni	penicillin	Cambodia	0	0	.	0	.	.	0.629	98.46
s. pneumoni	penicillin	China	18	907.47	0	0.679	98.53	68.12	0.629	98.46
s. pneumoni	penicillin	Malaysia	2	12.07	0.002	0.124	80.67	5.17	0.629	98.46
s. pneumoni	penicillin	meningitis	10	88.96	0	0.322	91.11	11.24	0.629	98.46
s. pneumoni	penicillin	sepsis	11	423.2	0	0.252	97.63	42.22	0.629	98.46
s. pneumoni	penicillin	2011	1	48.68	0	2.813	97.95	48.68	0.629	98.46
s. pneumoni	penicillin	2016	15	502.09	0	0.585	97.26	36.45	0.629	98.46
s. pneumoni	penicillin	2020	4	463.38	0	0.616	99.3	143.01	0.629	98.46
s. pneumoni	3gc	Cambodia	0	0	.	0	.	.	0.311	96.38
s. pneumoni	3gc	China	18	347.33	0	0.304	96.05	25.33	0.311	96.38
s. pneumoni	3gc	Malaysia	0	0	.	0	.	.	0.311	96.38
s. pneumoni	3gc	meningitis	9	95.51	0	0.434	91.32	11.52	0.311	96.38
s. pneumoni	3gc	sepsis	10	186.5	0	0.152	95.56	22.54	0.311	96.38
s. pneumoni	3gc	2011	2	18.36	0	0.87	93.09	14.47	0.311	96.38
s. pneumoni	3gc	2016	12	170.57	0	0.276	94.09	16.93	0.311	96.38
s. pneumoni	3gc	2020	4	49.82	0	0.199	96.72	30.49	0.311	96.38
s. pneumoni	carb	China	8	159.1	0	0.209	94.71	18.9	0.209	94.71
s. pneumoni	carb	meningitis	2	7.5	0.023	0.077	73.19	3.73	0.209	94.71
s. pneumoni	carb	sepsis	5	135.04	0	0.179	95.27	21.13	0.209	94.71
s. pneumoni	carb	2011	0	0	.	0	.	.	0.209	94.71
s. pneumoni	carb	2016	6	59.54	0	0.181	92.36	13.08	0.209	94.71
s. pneumoni	carb	2020	0	0	.	0	.	.	0.209	94.71
s. pneumoni	chlor	China	10	604.75	0	0.379	98.25	57.02	0.379	98.25
s. pneumoni	chlor	meningitis	2	5.23	0.073	0.048	63.44	2.74	0.379	98.25
s. pneumoni	chlor	sepsis	7	593.49	0	0.475	98.91	91.91	0.379	98.25
s. pneumoni	chlor	2011	0	0	.	0	.	.	0.379	98.25
s. pneumoni	chlor	2016	6	17.08	0.009	0.026	63.46	2.74	0.379	98.25
s. pneumoni	chlor	2020	2	579.04	0	1.334	99.8	504.55	0.379	98.25

S1 Figure 37 Proportion of S. aureus isolates susceptibile to Ampicillin by subgroup

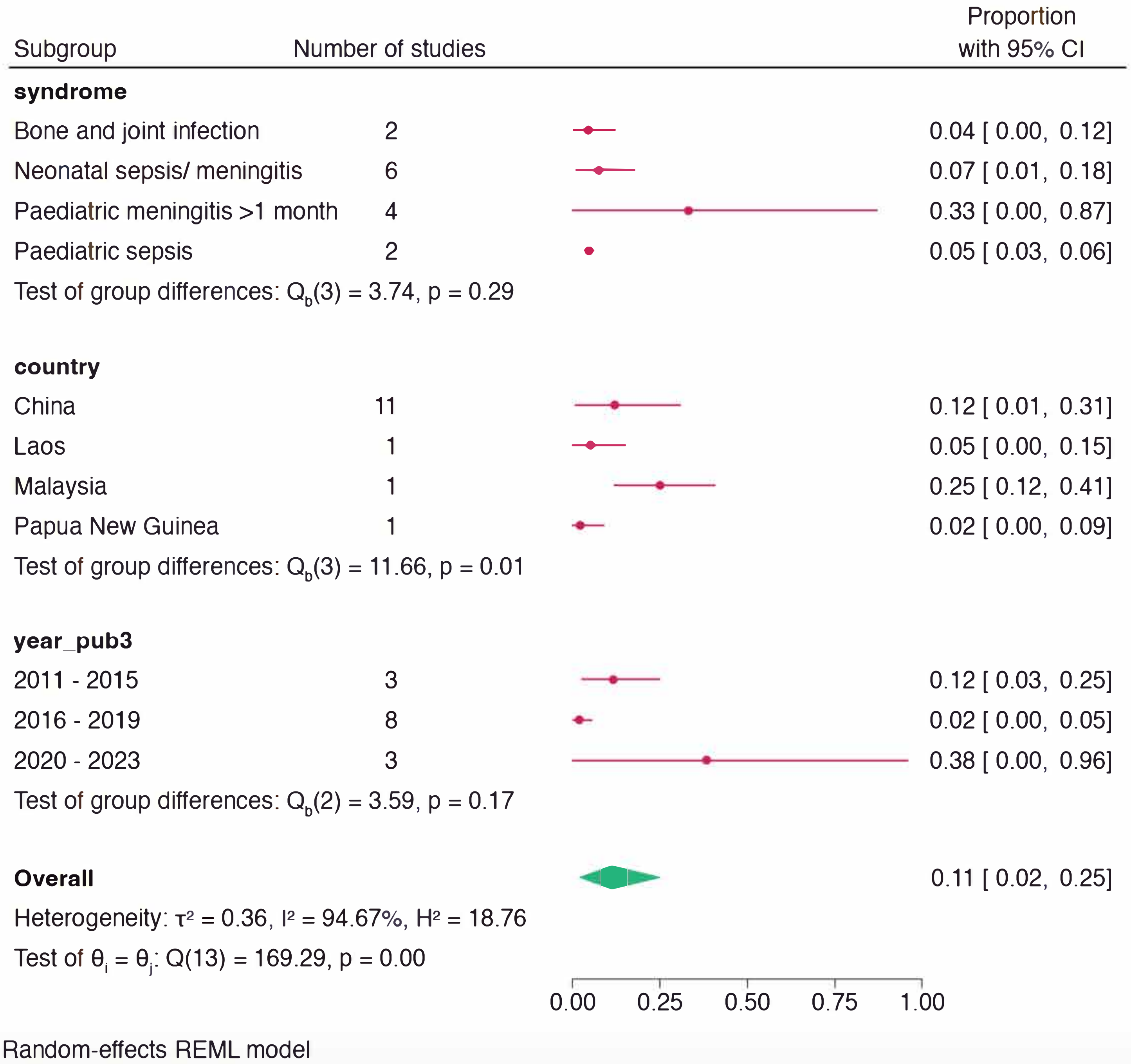

S1 Figure 38 Proportion of S. aureus isolates susceptibile to Flucloxacillin by subgroup

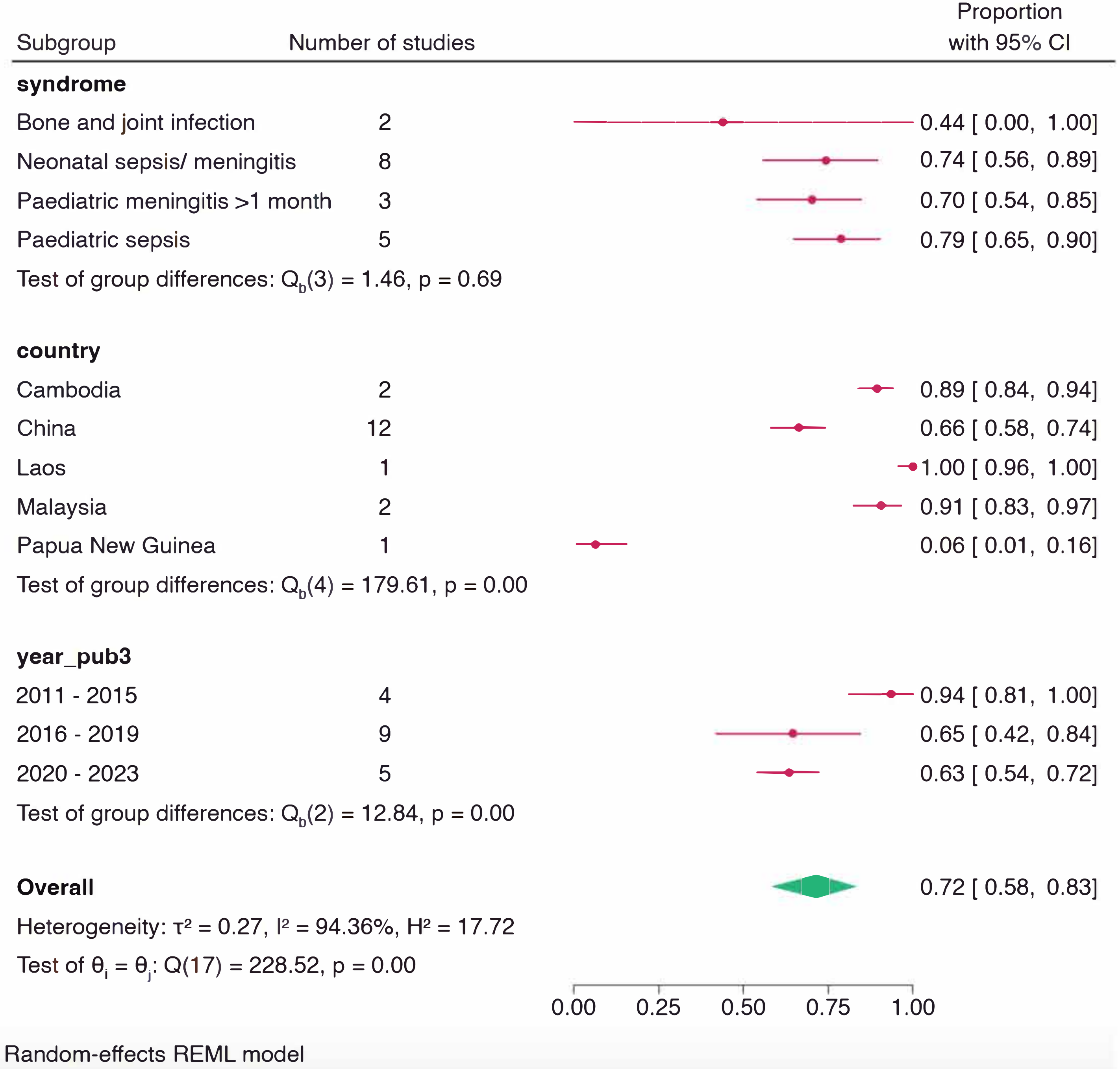

S1 Figure 39 Proportion of S. aureus isolates susceptibile to Vancomycin by subgroup

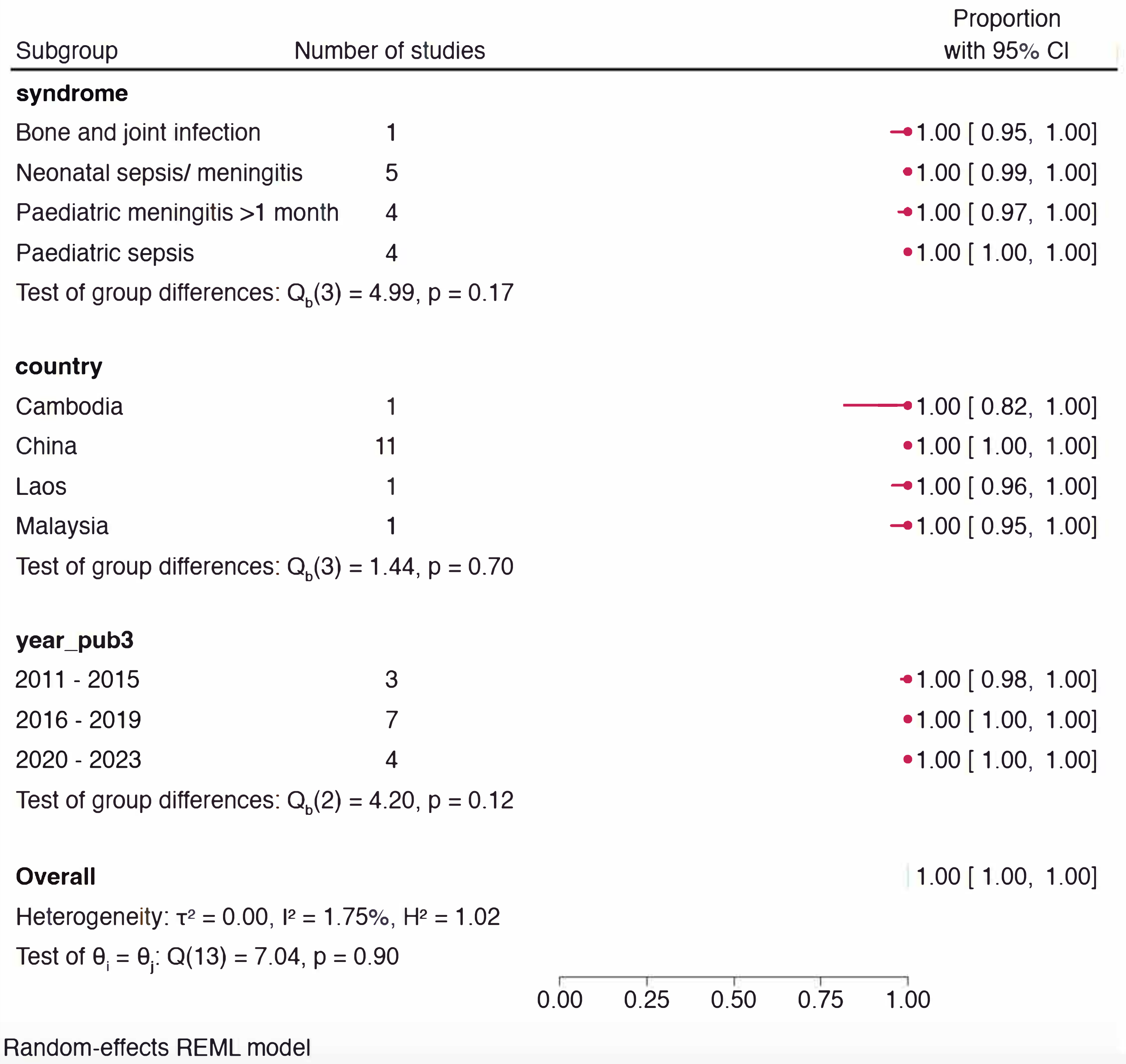

SI Figure 40

Org	Ab	subgroup	df	q	pvalue	tau	i2	h2	Overall_Tau2	Overall_I2
s. aureus	penicillin	China	10	157.22	0	0.448	95.27	21.13	0.36	94.67
s. aureus	penicillin	Laos	0	0	.	0	.	.	0.36	94.67
s. aureus	penicillin	Malaysia	0	0	.	0	.	.	0.36	94.67
s. aureus	penicillin	New Gu~a	0	0	.	0	.	.	0.36	94.67
s. aureus	penicillin	Bone and joi	1	1.47	0.225	0.011	31.96	1.47	0.36	94.67
s. aureus	penicillin	Neonatal sep	5	16.82	0.005	0.084	69.14	3.24	0.36	94.67
s. aureus	penicillin	Paediatric m	3	46.22	0	0.989	90.75	10.81	0.36	94.67
s. aureus	penicillin	Paediatric s-	1	0.62	0.43	0	0	1	0.36	94.67
s. aureus	penicillin	2011	2	6.55	0.038	0.061	69.61	3.29	0.36	94.67
s. aureus	penicillin	2016	7	6.52	0.481	0.001	2.17	1.02	0.36	94.67
s. aureus	penicillin	2020	2	150.94	0	1.393	98.82	84.82	0.36	94.67
s. aureus	flucloxacillin	Cambodia	1	0.27	0.601	0	0	1	0.27	94.36
s. aureus	flucloxacillin	China	11	24.3	0.012	0.042	68.26	3.15	0.27	94.36
s. aureus	flucloxacillin	Laos	0	0	.	0	.	.	0.27	94.36
s. aureus	flucloxacillin	Malaysia	1	0.2	0.651	0	0	1	0.27	94.36
s. aureus	flucloxacillin	New Gu~a	0	0	.	0	.	.	0.27	94.36
s. aureus	flucloxacillin	Bone and joi	1	69.13	0	1.625	98.55	69.13	0.27	94.36
s. aureus	flucloxacillin	Neonatal sep	7	66.77	0	0.246	87.97	8.31	0.27	94.36
s. aureus	flucloxacillin	Paediatric m	2	0.33	0.847	0	0	1	0.27	94.36
s. aureus	flucloxacillin	Paediatric s-	4	58.62	0	0.091	92.09	12.64	0.27	94.36
s. aureus	flucloxacillin	2011	3	10	0.019	0.08	69.11	3.24	0.27	94.36
s. aureus	flucloxacillin	2016	8	151.04	0	0.359	93.16	14.61	0.27	94.36
s. aureus	flucloxacillin	2020	4	9.24	0.055	0.026	63.74	2.76	0.27	94.36
s. aureus	vancomycin	Cambodia	0	0	.	0	.	.	0	1.75
s. aureus	vancomycin	China	10	5.6	0.848	0	0	1	0	1.75
s. aureus	vancomycin	Laos	0	0	.	0	.	.	0	1.75
s. aureus	vancomycin	Malaysia	0	0	.	0	.	.	0	1.75
s. aureus	vancomycin	Bone and joi	0	0	.	0	.	.	0	1.75
s. aureus	vancomycin	Neonatal sep	4	0.31	0.989	0	0	1	0	1.75
s. aureus	vancomycin	Paediatric m	3	0.48	0.922	0	0	1	0	1.75
s. aureus	vancomycin	Paediatric s-	3	1.26	0.739	0	0.02	1	0	1.75
s. aureus	vancomycin	2011	2	0	1	0	0	1	0	1.75
s. aureus	vancomycin	2016	6	1.57	0.955	0	0	1	0	1.75
s. aureus	vancomycin	2020	3	1.27	0.736	0	0	1	0	1.75

S1 Figure 41 Proportion of Group B Streptococcus isolates susceptibile to Ampicillin by subgroup

Subgroup	Number of studies	Proportion with 95% CI
**syndrome**		
Neonatal sepsis/ meningitis	5	1.00 [1.00, 1.00]
Paediatric meningitis >1 month	5	1.00 [0.95, 1.00]
Paediatric sepsis	2	1.00 [1.00, 1.00]
Test of group differences: $Q_b(2) = 3.76$, $p = 0.15$		
**country**		
China	11	1.00 [1.00, 1.00]
Malaysia	1	1.00 [0.85, 1.00]
Test of group differences: $Q_b(1) = 0.29$, $p = 0.59$		
**year_pub3**		
2011 - 2015	1	1.00 [0.96, 1.00]
2016 - 2019	8	1.00 [1.00, 1.00]
2020 - 2023	3	1.00 [1.00, 1.00]
Test of group differences: $Q_b(2) = 0.73$, $p = 0.69$		
**Overall**		1.00 [1.00, 1.00]

Heterogeneity: $\tau^2 = 0.00$, $I^2 = 0.00\%$, $H^2 = 1.00$

Test of $\theta_i = \theta_j$: $Q(11) = 5.66$, $p = 0.90$

0.00 0.25 0.50 0.75 1.00

Random-effects REML model

S1 Figure 42 Proportion of Group B Streptococcus isolates susceptibile to 3rd Generation Cephalosporins by subgroup

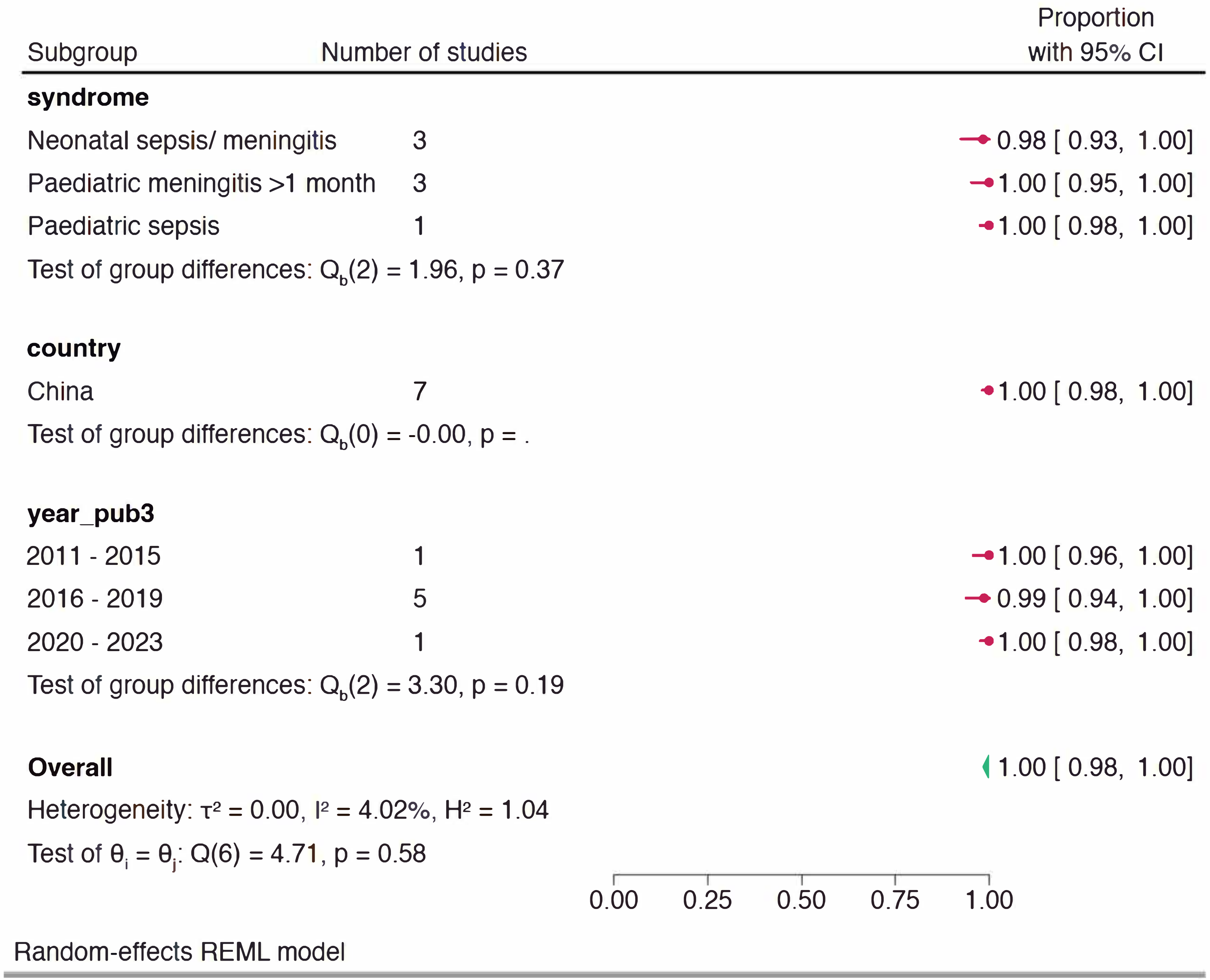

S1 Figure 43 Proportion of Group B Streptococcus isolates susceptibile to Carbapenems by subgroup

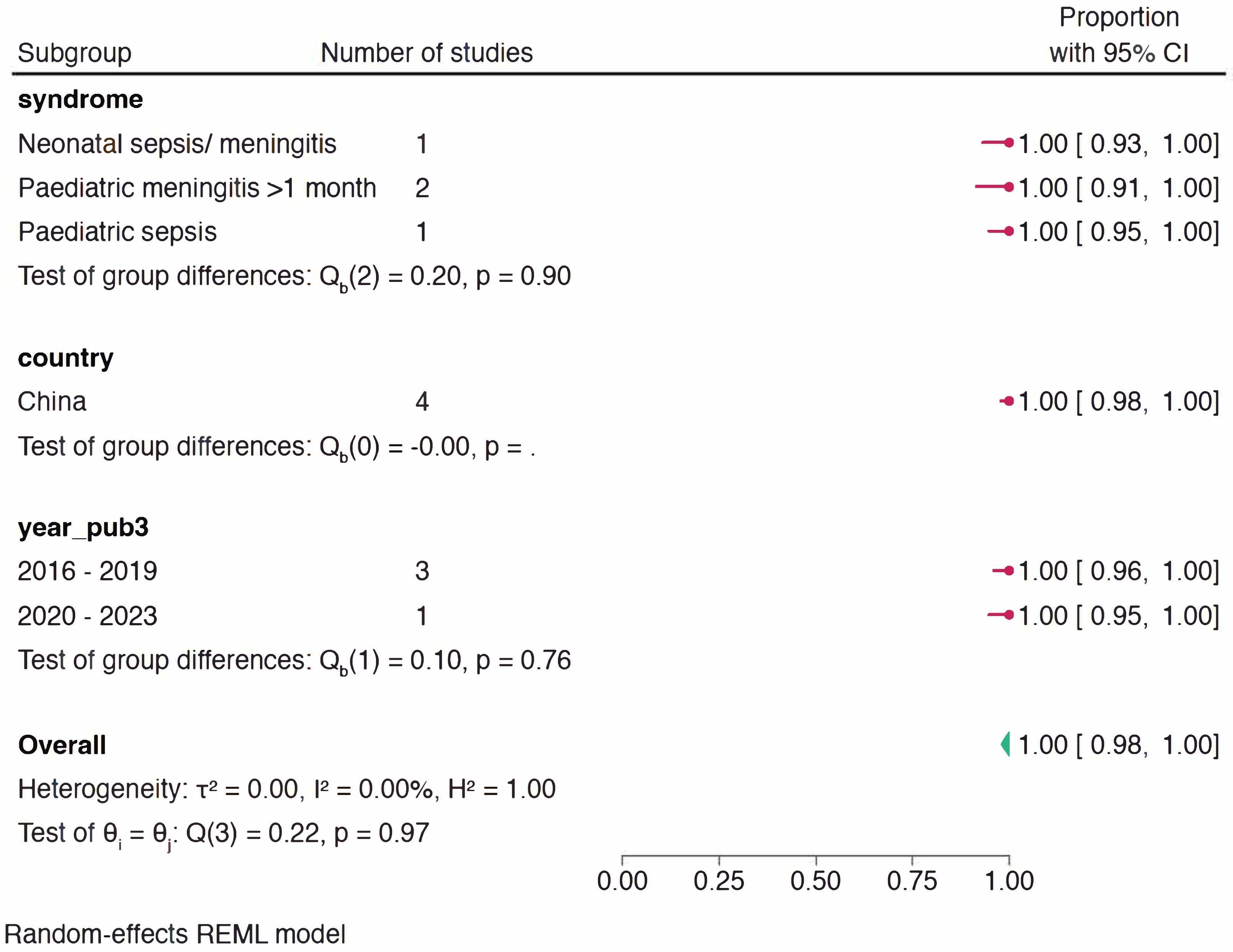

S1 Figure 44 Proportion of Group B Streptococcus isolates susceptibile to Vancomycin by subgroup

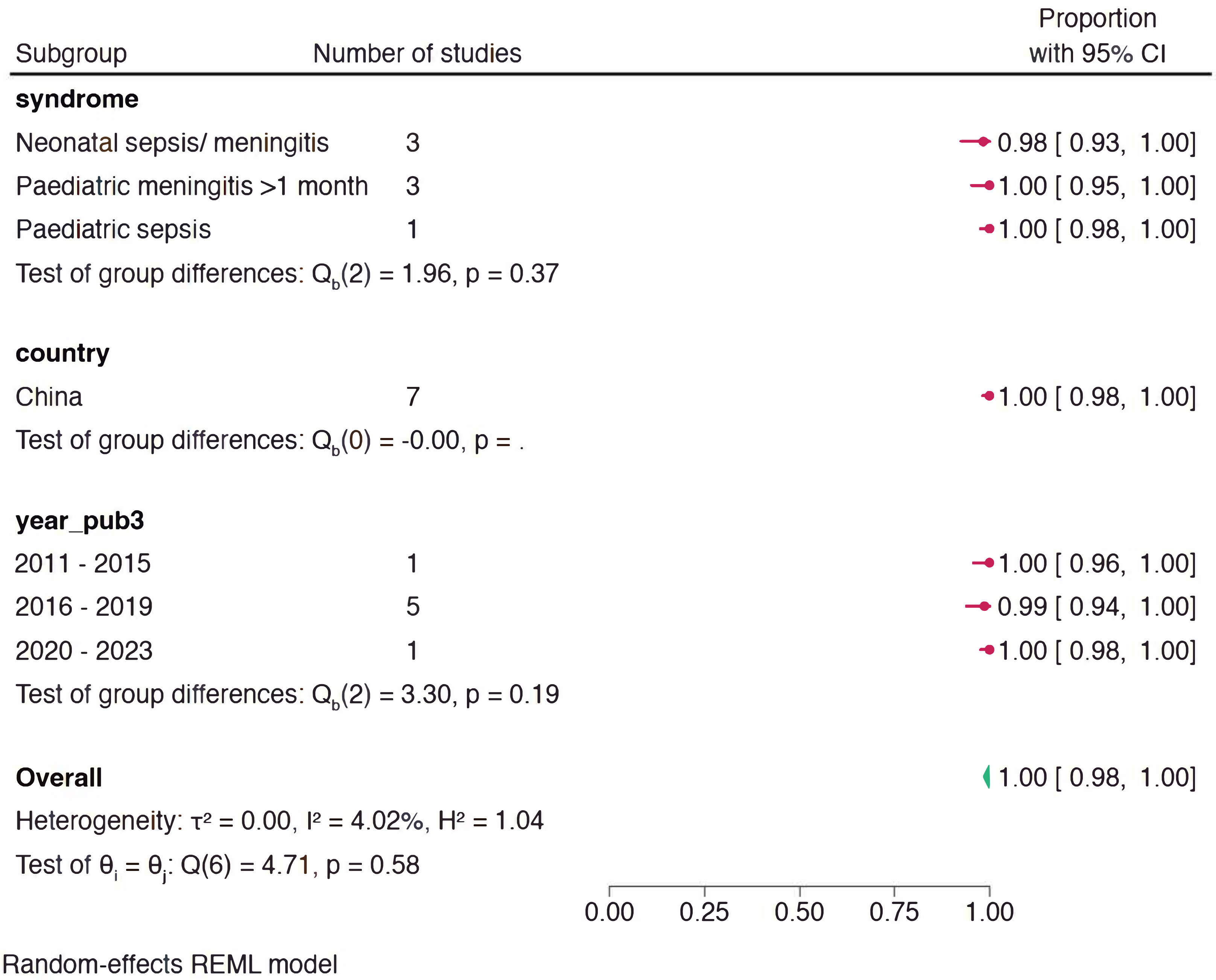

S1 Figure 45

Org	Ab	subgroup	df	q	pvalue	tau	i2	h2	Overall_Tau2	Overall_I2
GBS	Ampicillin	China	10	5.36	0.866	0	0	1	0	0
GBS	Ampicillin	Malaysia	0	0	.	0	.	.	0	0
GBS	Ampicillin	Neonatal sep	4	0.64	0.958	0	0	1	0	0
GBS	Ampicillin	Paediatric m	4	0.73	0.947	0	0	1	0	0
GBS	Ampicillin	Paediatric s-	1	0.52	0.472	0	0	1	0	0
GBS	Ampicillin	2011	0	0	.	0	.	.	0	0
GBS	Ampicillin	2016	7	1.19	0.991	0	0	1	0	0
GBS	Ampicillin	2020	2	3.73	0.155	0	0	1	0	0
GBS	3gc	China	6	4.71	0.581	0.002	4.02	1.04	0.002	4.02
GBS	3gc	Neonatal sep	2	2.62	0.27	0.014	28.26	1.39	0.002	4.02
GBS	3gc	Paediatric m	2	0.05	0.977	0	0	1	0.002	4.02
GBS	3gc	Paediatric s-	0	0	.	0	.	.	0.002	4.02
GBS	3gc	2011	0	0	.	0	.	.	0.002	4.02
GBS	3gc	2016	4	1.41	0.842	0	0	1	0.002	4.02
GBS	3gc	2020	0	0	.	0	.	.	0.002	4.02
GBS	carb	China	3	0.22	0.975	0	0	1	0	0
GBS	carb	Neonatal sep	0	0	.	0	.	.	0	0
GBS	carb	Paediatric m	1	0.01	0.904	0	0	1	0	0
GBS	carb	Paediatric s-	0	0	.	0	.	.	0	0
GBS	carb	2016	2	0.12	0.941	0	0	1	0	0
GBS	carb	2020	0	0	.	0	.	.	0	0
GBS	vancomycin	China	6	4.71	0.581	0.002	4.02	1.04	0.002	4.02
GBS	vancomycin	Neonatal sep	2	2.62	0.27	0.014	28.26	1.39	0.002	4.02
GBS	vancomycin	Paediatric m	2	0.05	0.977	0	0	1	0.002	4.02
GBS	vancomycin	Paediatric s-	0	0	.	0	.	.	0.002	4.02
GBS	vancomycin	2011	0	0	.	0	.	.	0.002	4.02
GBS	vancomycin	2016	4	1.41	0.842	0	0	1	0.002	4.02
GBS	vancomycin	2020	0	0	.	0	.	.	0.002	4.02

S1 Figure 46 Meta-regression by Year of Publication and Bubble Plots

Organism	Antibiotic	co-efficient	95% CI	p-value
E. coli	**Ampicillin**	**0.07**	**(0.02 to 0.13)**	**0.013**
E. coli	Gentamicin	0.01	(-0.03 to 0.04)	0.730
E. coli	3GC	-0.06	(-0.12 to 0)	*0.069*
E. coli	**Carbapenem**	**-0.04**	**(-0.07 to -0.02)**	**0.001**
Klebsiella spp.	Gentamicin	0.02	(-0.06 to 0.1)	0.664
Kleb	3GC	-0.08	(-0.17 to 0.02)	0.128
***Klebsiella* spp.**	**Carbapenem**	**-0.10**	**(-0.21 to 0)**	***0.051***
S. aureus	Ampicillin	0.05	(-0.06 to 0.17)	0.373
S. aureus	**Flucloxacillin**	**-0.09**	**(-0.16 to -0.01)**	**0.025**
S. aureus	Vancomycin	0.02	(-0.01 to 0.04)	0.122
S. pneumoniae	Ampicillin	0.03	(-0.13 to 0.2)	0.683
S. pneumoniae	3GC	0.07	(-0.03 to 0.18)	0.180
S. pneumoniae	Carbapenem	0.11	(-0.03 to 0.25)	0.126
S. pneumoniae	Chloramphenicol	-0.09	(-0.27 to 0.09)	0.320
Salmonella spp.	Ampicillin	0.00	(-0.07 to 0.08)	0.902
Salmonella spp.	3GC	-0.02	(-0.09 to 0.05)	0.626
Salmonella spp.	Carbapenem	-0.02	(-0.06 to 0.02)	0.274
Salmonella spp.	Naldixic acid	-0.01	(-0.18 to 0.16)	0.891
Salmonella spp.	Ciprofloxacin	0.03	(-0.07 to 0.14)	0.517
Salmonella spp.	Co-trimoxazole	0.02	(-0.08 to 0.12)	0.702
P. aeruginosa	Gentamicin	-0.06	(-0.24 to 0.11)	0.477
P. aeruginosa	Carbapenem	-0.04	(-0.13 to 0.06)	0.447
Acinetobacter spp.	Gentamicin	-0.04	(-0.11 to 0.02)	0.190
***Acinetobacter* spp.**	**Carbapenem**	**-0.05**	**(-0.09 to 0)**	***0.055***
H. influenzae	3GC	0.09	(-0.44 to 0.62)	0.729
H. influenzae	Ampicillin	-0.20	(-1.7 to 1.3)	0.792
S. agalactiae	Ampicillin	0.02	(-0.02 to 0.06)	0.421
S. agalactiae	3GC	0.01	(-0.08 to 0.09)	0.840
S. agalactiae	carb	0.02	(-0.13 to 0.17)	0.807
S. agalactiae	Vancomycin	0.04	(-0.04 to 0.11)	0.346

S1 Figure 47 Bubble Plots

***E. coli vs* Ampicillin**

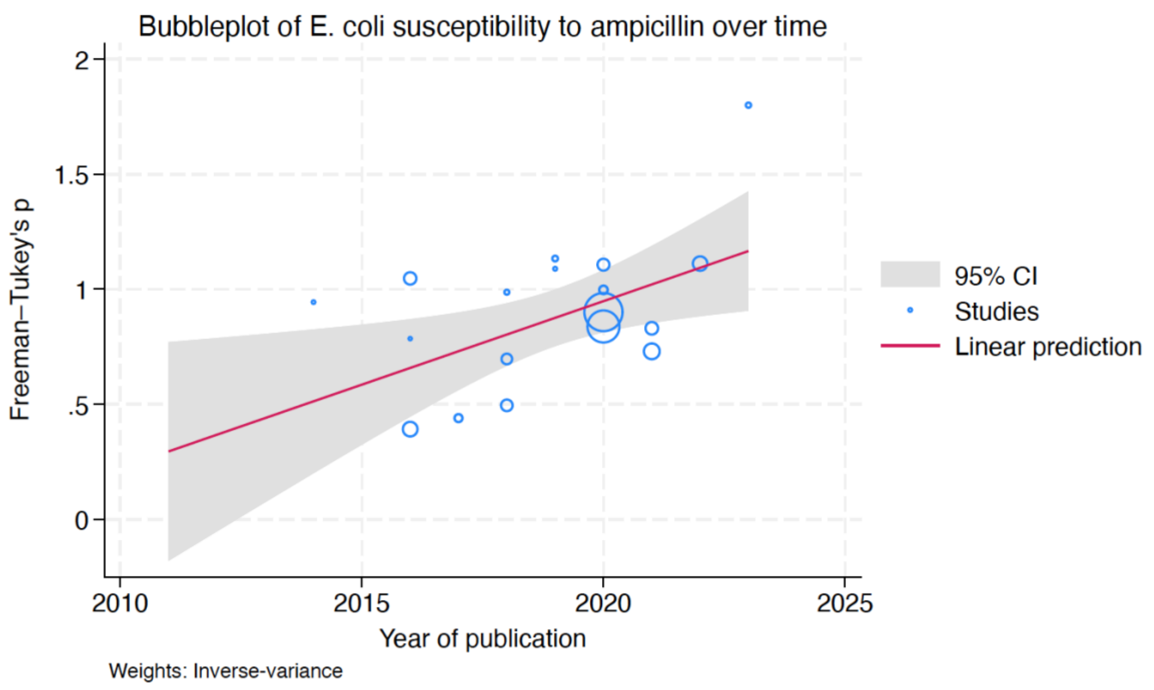

***E. coli vs* Carbapenem**

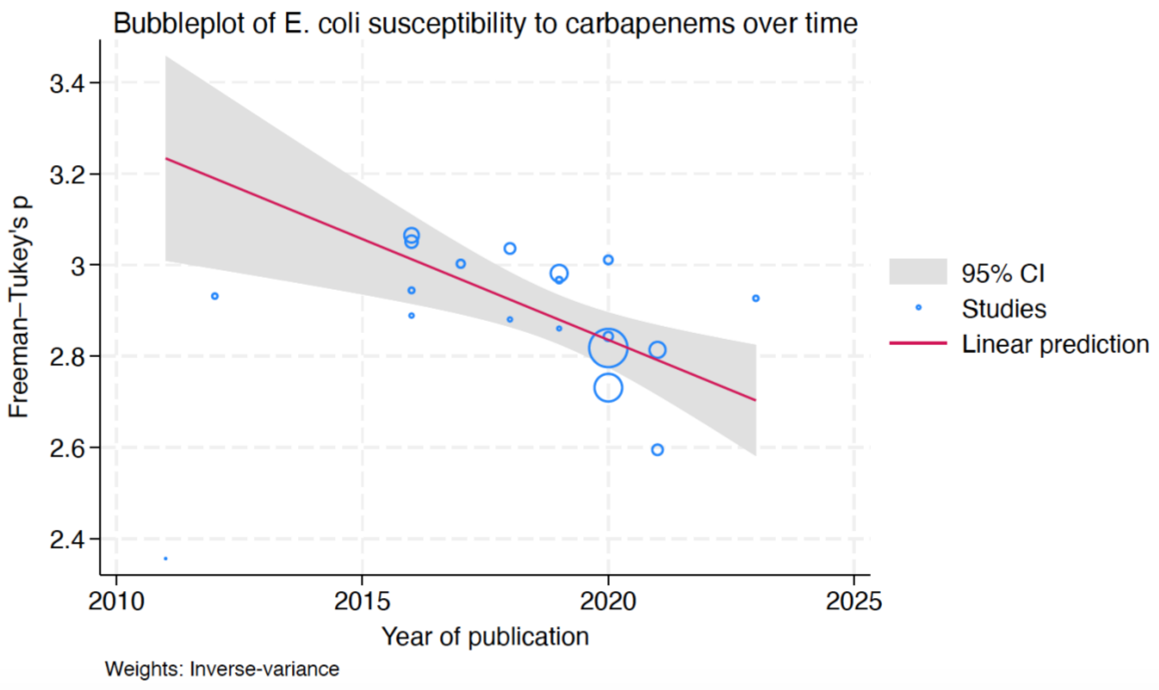

Klebsiella spp. *vs* Carbapenem

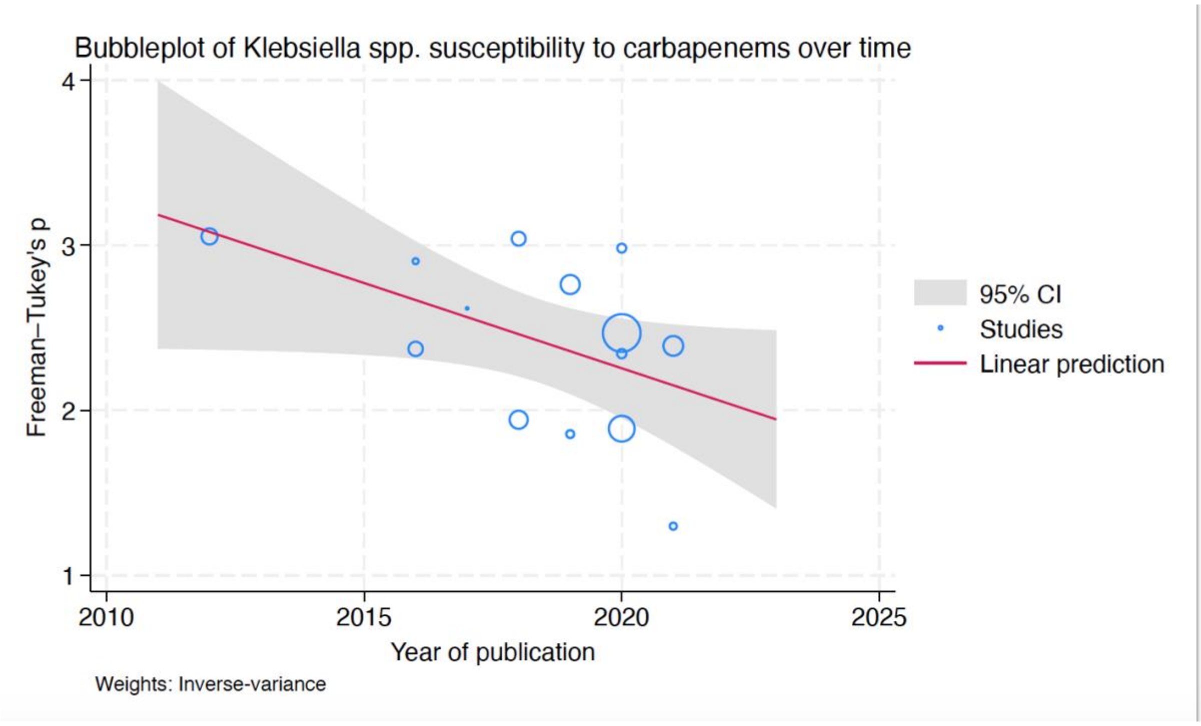

S. aureus vs Flucloxacillin

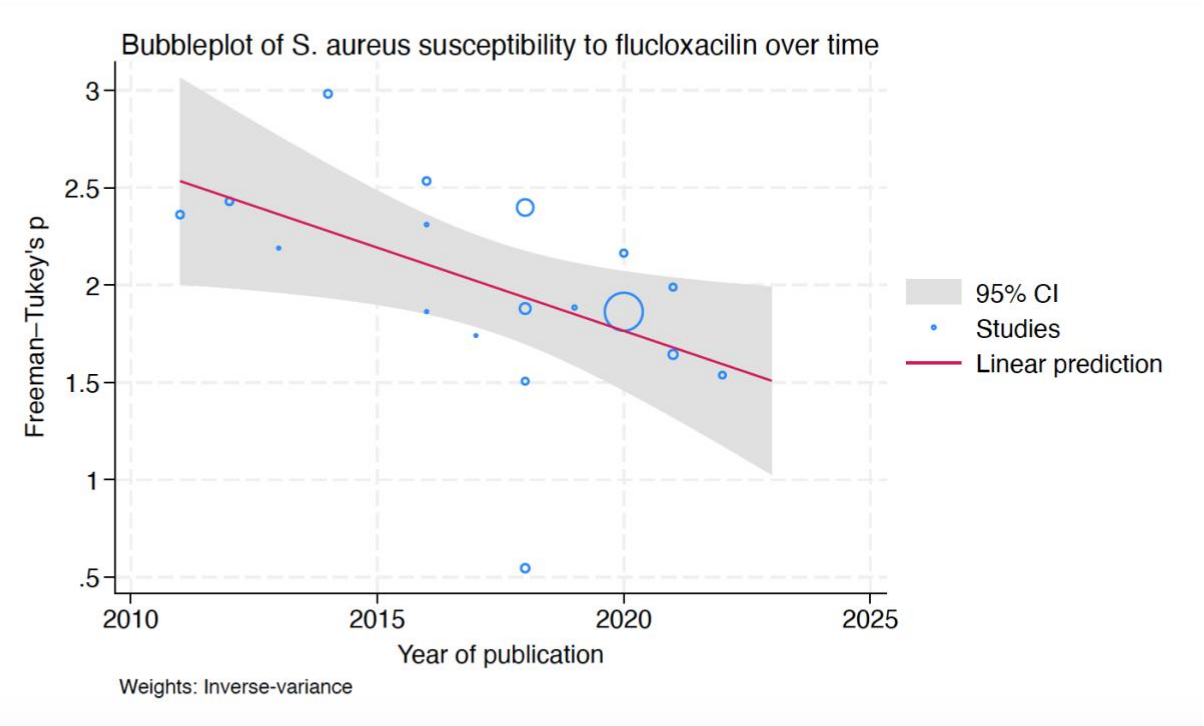

***Acinetobacter* spp. vs Carbapenem**

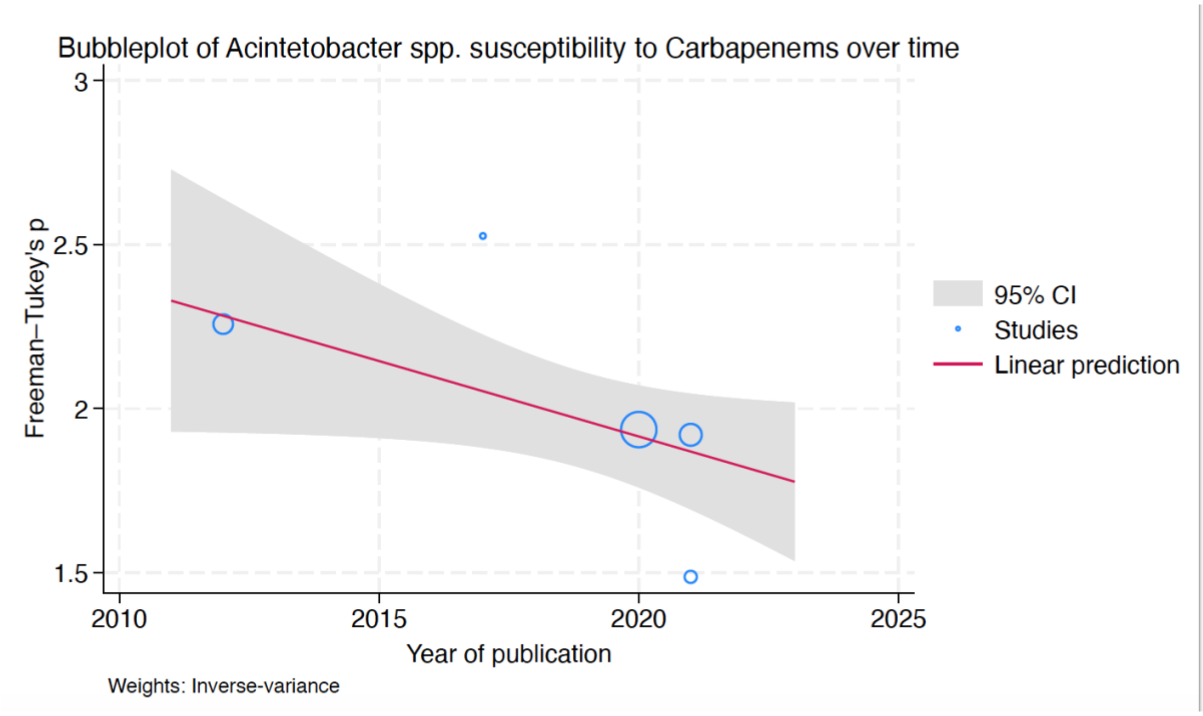

***E. coli vs* Ampicillin**

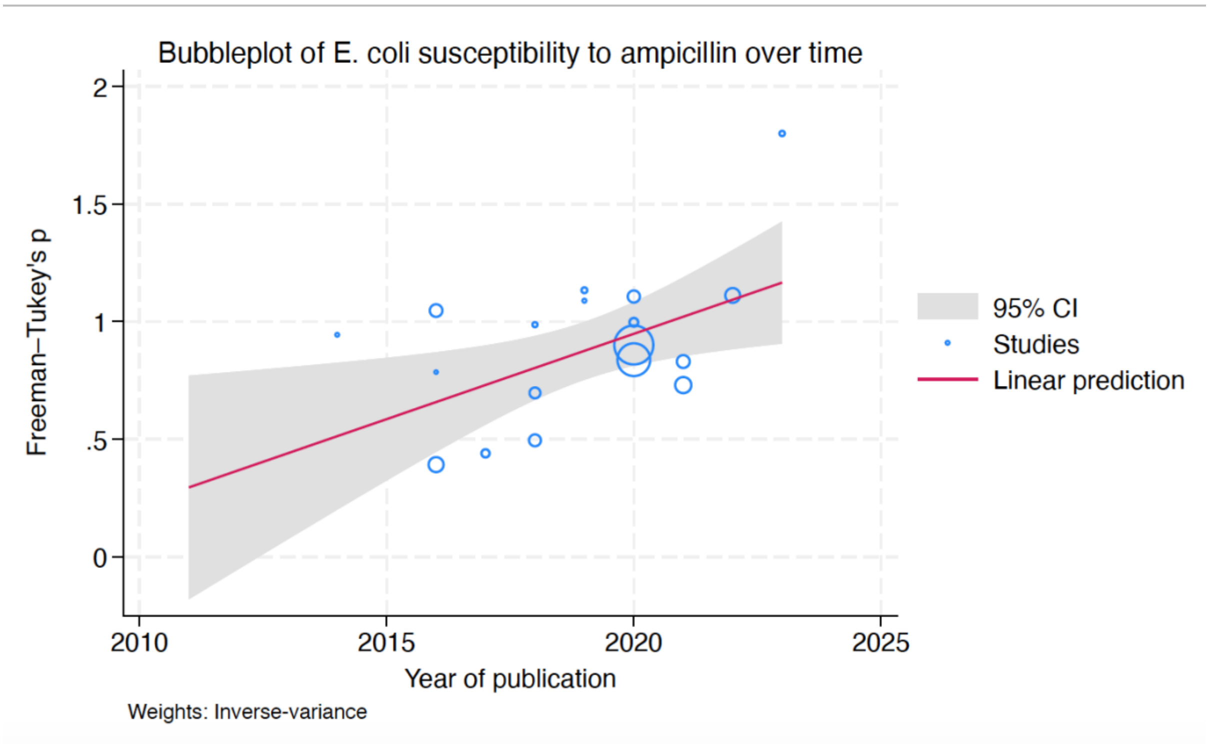

S1 Figure 48 Egger's regression for small-study effects

Organism	Antibiotic	p-value
E. coli	Ampicillin	0.1857
E. coli	Gentamicin	0.8481
E. coli	3GC	0.5386
E. coli	Carbapenem	0.7145
Klebsiella spp.	Gentamicin	0.1539
Klebsiella spp.	3GC	0.9772
Klebsiella spp.	Carbapenem	0.975
S. aureus	Ampicillin	0.9866
S. aureus	Flucloxacillin	0.9907
S. aureus	Vancomycin	**0.008**
S. pneumoniae	Ampicillin	**0.0064**
S. pneumoniae	3GC	0.3004
S. pneumoniae	Carbapenem	0.7453
S. pneumoniae	Chloramphenicol	0.3807
Salmonella spp.	Ampicillin	0.6048
Salmonella spp.	3GC	0.1993
Salmonella spp.	Carbapenem	0.9938
Salmonella spp.	Naldixic acid	0.4023
Salmonella spp.	Ciprofloxacin	0.8495
Salmonella spp.	Co-trimoxazole	0.4998
Salmonella spp.	Azithromycin	.
P. aeruginosa	Gentamicin	0.879
P. aeruginosa	Carbapenem	0.8421
Acinetobacter spp.	Gentamicin	0.9498
Acinetobacter spp.	Carbapenem	0.8142
H. influenzae	3GC	0.9316
H. influenzae	Ampicillin	0.1893
H. influenzae	Chloramphenicol	.
H. influenzae	Carbapenem	.
S. agalactiae	Ampicillin	**0.0506**
S. agalactiae	3GC	0.1711
S. agalactiae	Carbapenem	0.6401
S. agalactiae	Vancomycin	0.1711

S1 Figure 49 Funnel Plots

S. aureus

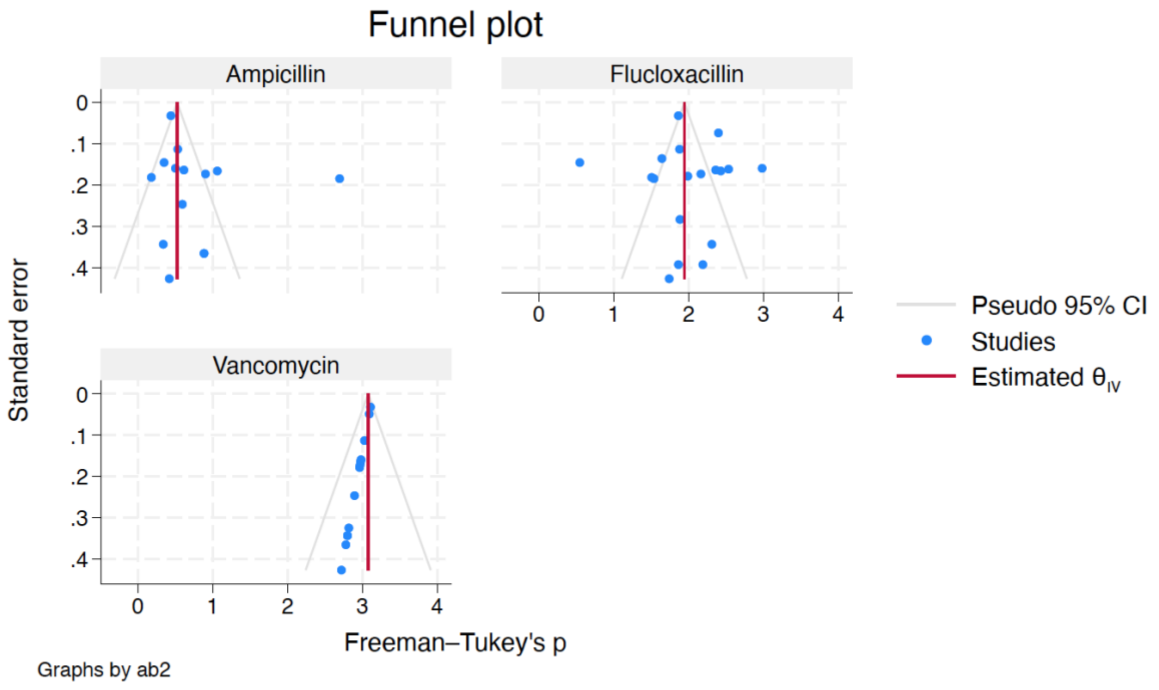

S. agalactiae

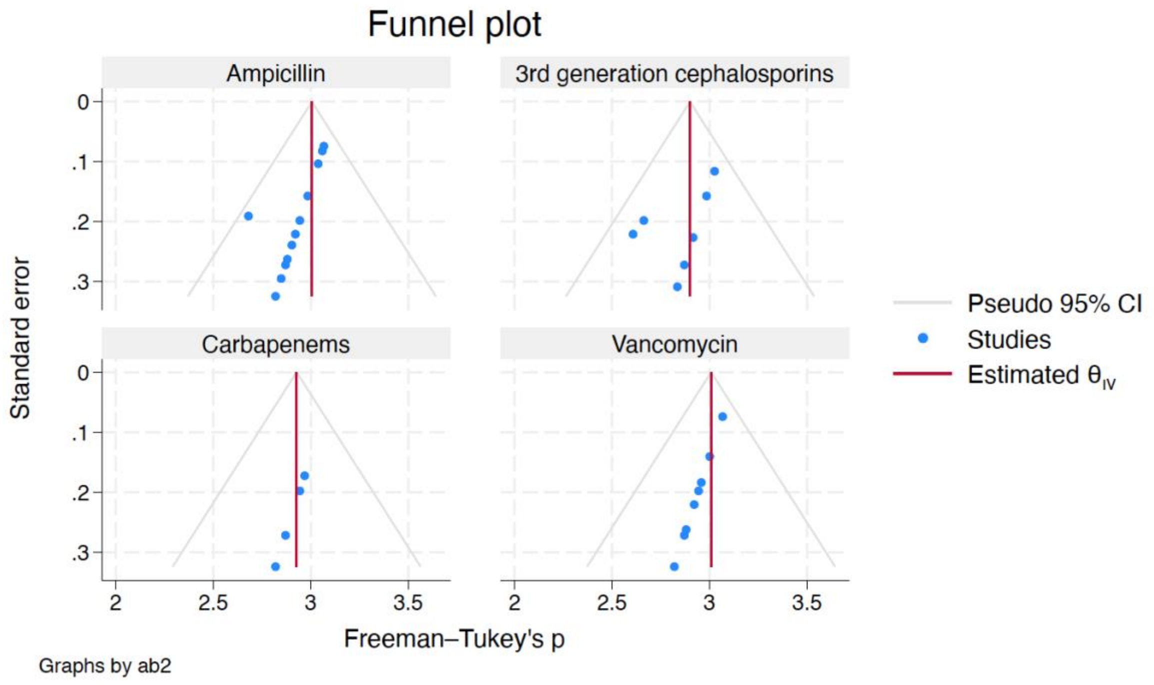

S. pneumoniae

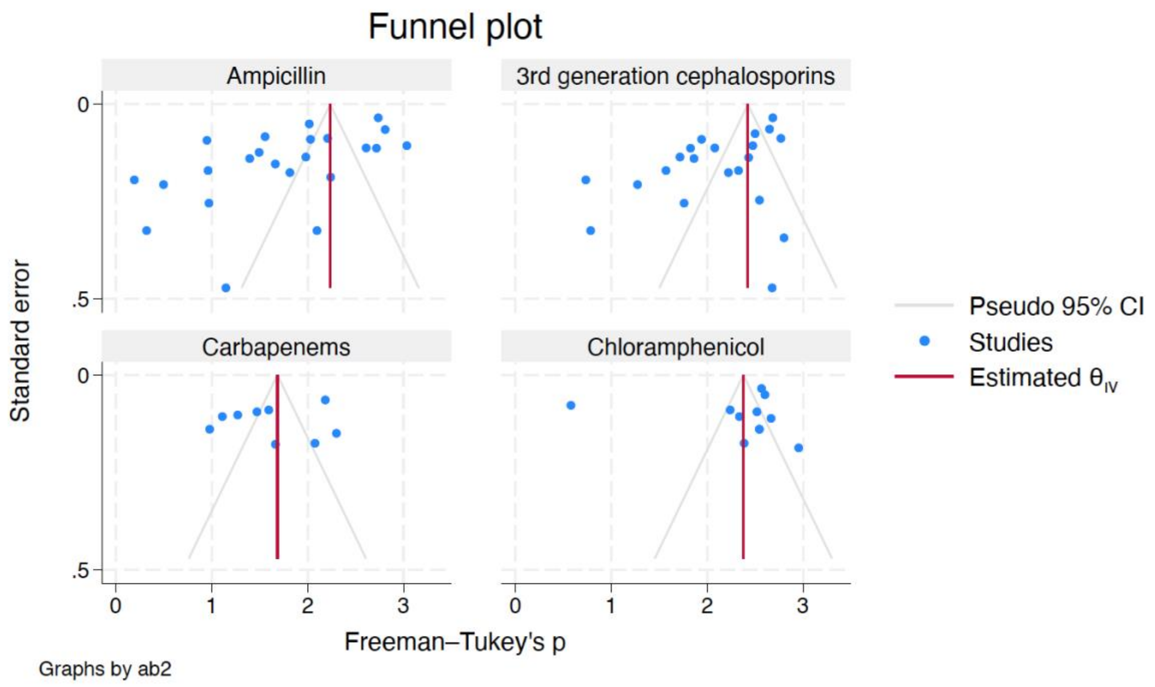

Supplement: Supplementary Data 1 [file mmc1.pdf]
